# National and subnational burden and attributable risk factors of osteoarthritis, rheumatoid arthritis, and low back pain in Iran: 1990–2019 findings of the Global Burden of Disease (GBD) Study

**DOI:** 10.1371/journal.pone.0344038

**Published:** 2026-07-06

**Authors:** Mahnaz Sanjari, Hossein Yarmohammadi, Sahar Saeedi Moghaddam, Mohammad Amin Habibi, Aida Fallahzadeh, Amir Hossein Behnoush, Shakiba Yousefi, Ali Sheikhy, Sima Noorali, Zahra Esfahani, Narges Ebrahimi, Noushin Fahimfar, Afshin Ostovar, Bagher Larijani

**Affiliations:** 1 Osteoporosis Research Center, Endocrinology and Metabolism Clinical Sciences Institute, Tehran University of Medical Sciences, Tehran, Iran; 2 Medical Students Research Committee, Shahed University, Tehran, Iran; 3 Non-Communicable Diseases Research Center, Endocrinology and Metabolism Population Sciences Institute, Tehran University of Medical Sciences, Tehran, Iran; 4 Kiel Institute for the World Economy, Kiel, Germany; 5 Clinical Research Development Center, Qom University of Medical Sciences, Qom, Iran; 6 Endocrinology and Metabolism Research Center, Endocrinology and Metabolism Clinical Sciences Institute, Tehran University of Medical Sciences, Tehran, Iran; Mashhad University of Medical Sciences, IRAN, ISLAMIC REPUBLIC OF

## Abstract

**Background:**

The prevalence of chronic diseases has increased concurrent with the increase in life expectancy and musculoskeletal disorders (MSDs) are among them. They have a high burden all over the world and are among the leading causes of years lived with disability (YLDs). MSDs are generally divided into six categories, and osteoarthritis (OA), rheumatoid arthritis (RA), and low back pain (LBP) are among most of the most prevalent MSDs. In this study, we aimed to assess the burden of these three disorders in Iran.

**Methods:**

Data were retrieved from the global burden of disease (GBD) study 2019 to investigate the epidemiological features of the burden of RA, OA, and LBP and the attributed burden to their risk factors in Iran and its provinces.

**Results:**

The total number of OA, RA, and LBP increased from 1990 to 2019. Although the age-standardized incidence rate (ASIR) of LBP decreased by −8.4%, the ASIR of OA and RA increased by 7.3% and 7.6% (reached 445.0 (95% uncertainty interval 395.9 to 501.9) and 5.0 (4.5 to 5.6) per 100,000 population in 2019, respectively). The age-standardized prevalence rate (ASPR) of OA and RA increased by 7.0% and 9.3%, respectively (5588.2 (5041.2 to 6228.6) and 109.1 (97.8 to 120.9) in 2019) while, the ASPR of LBP decreased by 9.5% (8,486.5 (7,533.8 to 9,528.3) in 2019). Females were more prone to establish OA and RA, but males were more predisposed to developing LBP. The highest OA incidence rate was presented in 55–59 years with a decreasing pattern over 59 years, 35–39 years with a decreasing pattern for older patients for RA, and 75–79 years with an increasing LBP incidence observed as age increased.

**Conclusion:**

LBP, OA, and RA are still significant health issues, and the number of patients is increasing, which imposes much burden. The increase in the prevalence of MSDs in Iran alarmingly highlights the need for immediate policies designed to reduce their burden. Therefore, epidemiological information helps make policies and reallocate sources that can improve healthcare services.

## 1. Introduction

Over previous decades, life expectancy has increased all over the world and as most chronic diseases such as musculoskeletal disorders (MSDs) have a higher incidence in older populations, their prevalence has increased. Moreover, with medical advancements, the prevalence of chronic diseases that were previously fatal has increased. Subsequently, more health efforts and expenditures are needed to maximize healthy life expectancy [1,2].

The global burden of disease (GBD) study provides epidemiological estimates on the disability and mortality of diseases and injuries to improve healthcare [3]. According to the GBD study, MSDs are categorized into six groups; osteoarthritis (OA), rheumatoid arthritis (RA), low back pain (LBP), neck pain, gout, and other musculoskeletal disorders [4]. Globally, 5.6% of total disability-adjusted life years (DALYs) and 15.9% of the years lived with disability (YLDs) in 2017 belonged to MSDs [2,5]. During 1990–2017, the number of incidence cases for MSDs had increased by 58%, including 52% for LBP and 102% for OA [6], and two-thirds of rehabilitation services allot to musculoskeletal conditions in adults [7]. A recent study in Iran showed a significant increase in the burden of MSDs and had the most increased percentage of MSD DALYs across the world. MSDs were the second and third causes of YLDs and DALYs in 2017, and LBP was the most common cause of disability [8].

Although age is a major risk factor, MSDs are not just appeared in the elderly; LBP, musculoskeletal injury, and inflammatory arthritis are also common in children, adolescents, and the middle-aged population [9]. Based on the GBD 2019 estimates, most risk factors for MSDs, such as old age, female sex, or genetic susceptibility, are not modifiable. However, others such as high body-mass index (BMI), high sugar-sweetened beverages (SSBs) intake, and occupational and ergonomic factors are amenable [10]. Some risk factors are attributable to the increased prevalence of MSDs. The aging population and decreased fertility rate in Iran contribute to the increasing MSDs burden [11]. Insufficient physical activity is also another major health concern and have a significant role in musculoskeletal morbidity [12,13].

Musculoskeletal health is essential for activity and well-being. A strong relationship between physical activity and chronic diseases such as cardiovascular conditions, osteoporosis, cancer, and diabetes in older people is present. Therefore, MSDs are an important component of multi-morbidity [14]. MSDs impress the social, community, and occupational dimensions of life and can lead to chronic pain, physical activity limitation, and increased risk of mental health disorders [15]. In addition to personal effects and direct costs for prevention, detection, treatment, and rehabilitation, MSDs impose an indirect cost by restricting usual activity and work productivity [16].

In Iran, the recent demographic changes showed a marked increase in life expectancy, elderly population, and decreased fertility rate, so MSDs are suspected to increase [17]. However, there need to be more studies about the national and subnational burden of MSDs. Estimating epidemiological metrics is a significant step for the clinician and political agenda to implement new plans for the prevention and management of diseases. In this study, we reported the national and subnational burden and risk factors of OA, RA, and LBP in Iran by age, sex, and socio-demographic index (SDI), and we also evaluated the trend of these MSDs during 1990–2019.

## 2. Methods

### 2.1. Overview

The GBD 2019 represents the global, regional, national, and subnational burden of 369 diseases and attributed burden to 87 risk factors for 204 countries in terms of prevalence, incidence, deaths, YLDs, DALYs, and years of life lost (YLLs) [18]. MSDs are categorized into six groups including osteoarthritis, rheumatoid arthritis, low back pain, neck pain, gout, and other musculoskeletal disorders (such as ankylosing spondylitis, lupus erythematosus, psoriasis arthritis, fibromyalgia, etc.) [18]. In this study, we described the fatal and nonfatal burden of OA, RA, and LBP in Iran and 31 provinces from 1990−2019 using the GBD 2019 findings based on age groups, and sex and we also grouped provinces according to the sociodemographic index (SDI). The International Statistical Classification of Diseases (ICD-10) code of investigated diseases is shown in S1 Table. The STROBE checklist is also available as supplementary material.

No individual data were reported in this paper and the information is based on aggregated pre‐existing online data. All the data included in this study are publicly available on the GBD 2019 Compare tool (https://vizhub.healthdata.org/gbd-compare/). Therefore, the need for ethical approval was not necessary in this study. This study was conducted in compliance with the Guidelines for Accurate and Transparent Health Estimates Reporting (GATHER) statement. Also, as there is no individual data reported in this study, participant consent was not needed.

### 2.2. Case definition

RA is an autoimmune disease that is confirmed based on the 1987 criteria by the American College of Rheumatology (ACR 1987) which four criteria are needed for a diagnosis. Criteria include: 1) morning stiffness at least one hour, 2) arthritis in three or more joints observed by physician, 3) arthritis of hand joints, 4) symmetric arthritis, 5) rheumatoid nodules, 6) the presence of rheumatoid factor, and 7) radiographic erosions on x-ray of hands [19].

OA is defined based on Kellgren-Lawrence grade 2–4 and pain one month out of the last 12 months. Grade 2 consists of osteophyte in the hip or knee and pain for at least one month out of the last 12. Grade 3–4 consists of osteophyte and joint space narrowing and deformity present at grade 4 [20].

LBP is defined as back pain with or without pain referred into one or two lower limbs that last for at least one day and low back is the area on the posterior between the lower margin of the 12th ribs and lower gluteal folds [21].

### 2.3. Data processing and disease model

Fatal burden metrics including total mortality and YLLs were calculated for RA. The cause of death was estimated by the ensemble model (CODEm) [22,23] and then the consistency between all causes of distribution and the sum of cause-specific estimates were computed by the cause of death correction (CODCorrect) [24]. YLL metric is an indicator of years of life lost due to premature mortality. YLLs were computed by summing the number of deaths that is multiplied by the number of years of life remaining at each age group [18,25]. Non-fatal burden metrics include YLDs and DALYs. YLD is described as years lived in less-than-ideal health. Multiplying the average of disability weight was used to calculate YLDs and DALYs were calculated as the sum of YLDs and YLLs. DisMod-MR 2.1 was the main tool in the estimation of the disability [18,26]. All rates were reported as age-standardized to the GBD world standard population to enable comparability over time and between locations. The 95% uncertainty intervals (UIs) were derived from 1,000 draws from the posterior distribution and defined as the 2.5th and 97.5th percentiles.

### 2.4. Attributed burden to risk factors

For determining the specific risk factor, the theoretical minimum risk exposure level (TMREL) was used for the counterfactual distribution of exposure for such specific risk factors without any change on the other risk factors. Also, it is applicable for the combination of risk factors to assess the attributable burden and population attributable fractions (PAFs). In order to assess the combined effects of risk factors, interpose the effect of risk factors on each other should be taken into account. The mediated matrix established in GBD 2017 [27] was applied to adjust the overestimation of PAFs and the attributable burden in the case of risk combination [28].

### 2.5. Socio-Demographic Index

SDI is a measure that identifies the spectrum of development according to income in capita, educational level, and fertility rate [29]. It ranges from 0 (less developed) to 1 (most developed).

### 2.6. Decomposition analysis

Three factors played a role in changing the incidence of these selected diseases from 1990 to 2019. The absolute number of the incident, changing age group structure, and population growth are factors that influenced the incidence of OA, RA, and LBP. Several steps were performed to investigate the involvement of each factor. 1) The age structure and incidence of selected diseases in 1990 were considered for the population of 2019; 2) The incidence of each disease according to the age groups of 1990 was used for the population and age arrangement of 2019; 3) The alteration of incident cases between steps 1 and 2 is considered to be related to the alteration of age structure during 1990–2019. Eventually, the variance between the incidence rate of step two and the true incidence of 2019 is considered for the alteration of the incidence rate [30]. All illustrations were done by R package V4.2.0 based on the extracted Excel datasets from the GBD Results Tool.

## 3. Results

### 3.1. Rheumatoid arthritis (RA)

**Fig 1:**
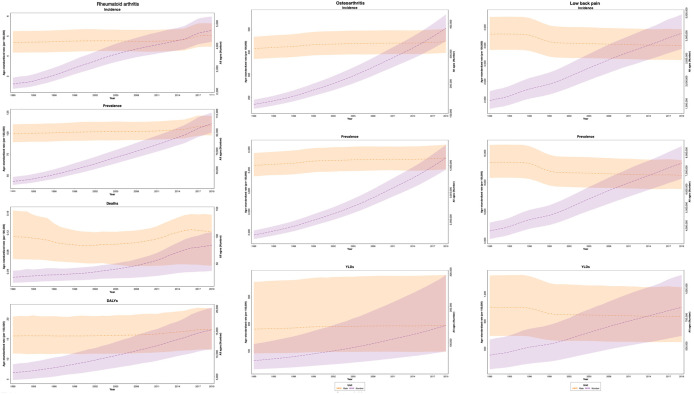
Time trend in the age-standardized rate and all ages number of epidemiological burden measures due RA (left), OA (middle) and LBP (right) from 1990 to 2019.

**Fig 2:**
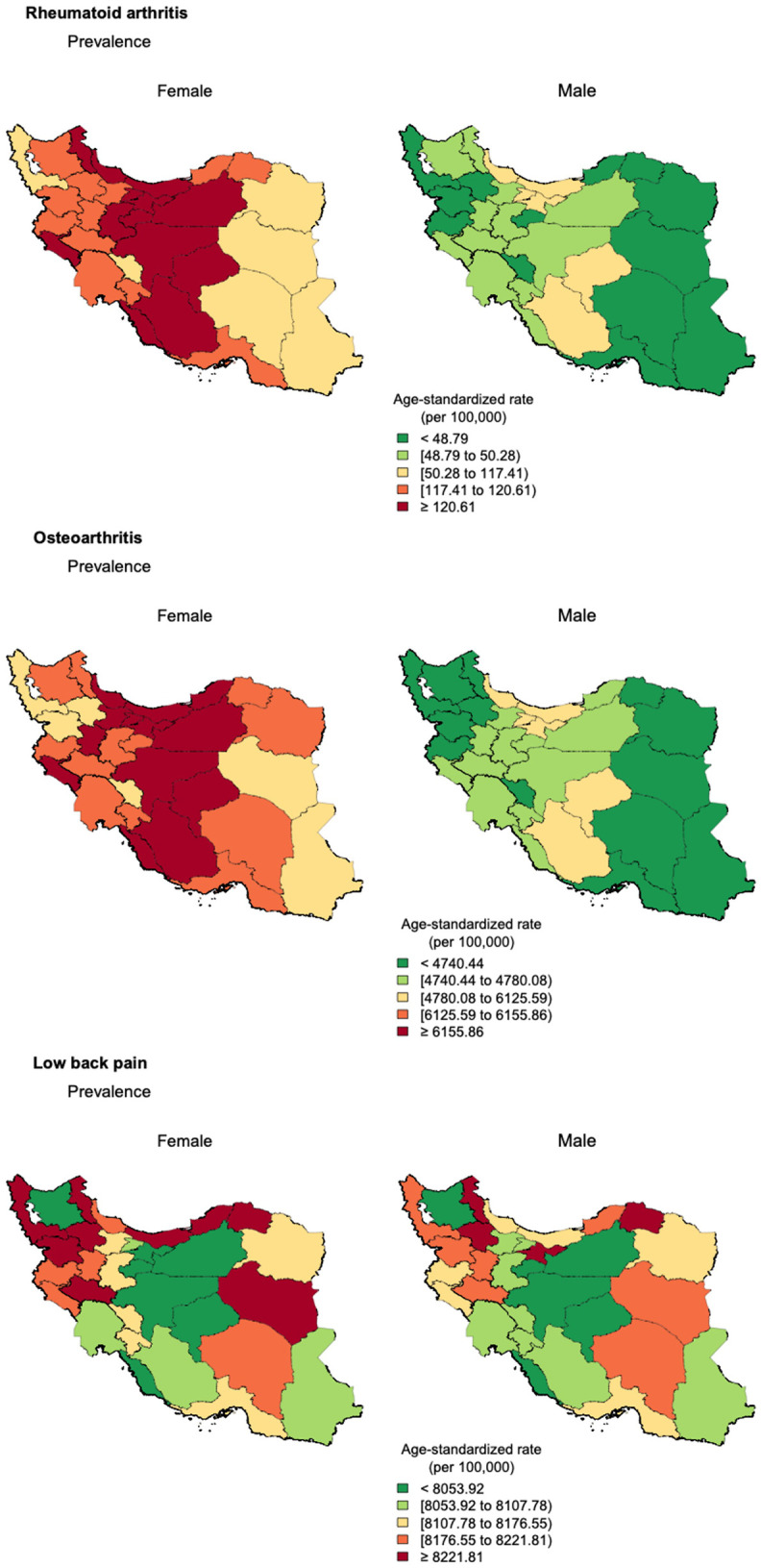
The subnational age-standardized rates of epidemiological burden measures of RA (top), OA (middle), and LBP (bottom) in 1990 and 2019, both sexes. (Contains information from OpenStreetMap and OpenStreetMap Foundation, which is made available under the Open Database License, https://www.openstreetmap.org/copyright).

The number of RA new cases increased from 2,314 (95% UI 2,071–2,597) in 1990–4,684 (4,128–5,303) in 2019. The age-standardized incidence rate (ASIR) of RA has increased by 7.6% from 4.7 (4.2 to 5.2) in 1990 to 5.0 (4.5 to 5.6) per 100,000 population in 2019 with a higher increase in females (8.0%) than males (6.6%). In 2019, the number of RA cases were 99,141 (88,570–110,731) and was higher in females compared to males (female: 76,897, male: 22,245). The age-standardized prevalence rate (ASPR) was 99.8 (89.6 to 110.9) and 109.1 (97.8 to 120.9) in 1990 and 2019, respectively which shows an increase of 9.3% (female: 8.5%, male: 7.0%). The age-standardized YLDs rate (ASYR) has increased by 9.1% from 13.4 (9.0 to 18.0) to 14.6 (9.9 to 19.8) with a higher rate in change among female (8.5%) than male (6.3%). The age-standardized rate of death was 0.1 (0.1 to 0.2) in 2019 which is enlarged by 9.5% and the rate of death was more increased in male (34.1%) than female (5%). The age-standardized rate of YLLs was 2.4 (1.4 to 3.6) in 1990 and 2.7 (1.1 to 3.6) in 2019 which interfaced 14% increase during 1990−2019. The rate of age-standardized YLLs in 1990 and 2019 was higher in female than male (3.7 and 4.0 vs 1.1 and 1.5, respectively), but the percentage of change was higher in male than female (31.7% vs 7.6%) (Table 1, Figs 1a and 2a). The lowest and highest increase in ASIR, ASPR, and ASYR during 1990−2019 were reported in Tehran (1.0%, −0.1%, 0.1%) and Ilam (13.9%, 17.4%, 16.7%) provinces, respectively (S2 Table).

**Table 1:** National burden of RA, OA, and LBP in 1990 and 2019, with percentage change by sex.

Cause	Measure	Age, Metric	Year						% Change (1990–2019)		
			1990			2019			Both	Female	Male
			Both	Female	Male	Both	Female	Male			
**Rheumatoid arthritis**	Incidence	All ages (number)	2,314 (2,071 to 2,597)	1,748 (1,560 to 1,963)	566 (500 to 641)	4,684 (4,128 to 5,303)	3,485 (3,064 to 3,932)	1,199 (1,044 to 1,369)	102.4 (87.5 to 117.2)	99.3 (84.4 to 114.8)	112.0 (97.7 to 126.5)
		Age-standardized rate (per 100,000)	4.7 (4.2 to 5.2)	7.0 (6.3 to 7.9)	2.4 (2.1 to 2.7)	5.0 (4.5 to 5.6)	7.6 (6.7 to 8.5)	2.6 (2.3 to 2.9)	7.6 (5.4 to 9.7)	8.0 (4.9 to 10.9)	6.6 (4.9 to 8.3)
	Prevalence	All ages (number)	37,946 (33,742 to 42,725)	29,117 (25,839 to 32,682)	8,828 (7,732 to 10,075)	99,141 (88,570 to 110,731)	76,897 (68,478 to 85,642)	22,245 (19,530 to 25,319)	161.3 (152.6 to 169.8)	164.1 (153.9 to 174.4)	152 (144.3 to 159.8)
		Age-standardized rate (per 100,000)	99.8 (89.6 to 110.9)	156.3 (140.4 to 173.7)	46.4 (41.0 to 52.7)	109.1 (97.8 to 120.9)	169.5 (152.0 to 187.8)	49.7 (43.9 to 56.2)	9.3 (7.0 to 11.8)	8.5 (5.4 to 11.8)	7.0 (5.2 to 8.7)
	Deaths	All ages (number)	25 (14 to 37)	19 (9 to 30)	6 (4 to 8)	83 (36 to 107)	61 (15 to 85)	22 (18 to 27)	238.6 (45.4 to 458)	225.4 (−13.1 to 553.6)	281.3 (176.4 to 484.7)
		Age-standardized rate (per 100,000)	0.1 (0.1 to 0.2)	0.2 (0.1 to 0.3)	0 (0 to 0.1)	0.1 (0.1 to 0.2)	0.2 (0 to 0.3)	0.1 (0.1 to 0.1)	9.5 (−54.6 to 88.6)	5 (−73.1 to 123.1)	34.1 (−0.5 to 97)
	DALYs	All ages (number)	5,863 (4,191 to 7,734)	4,448 (3,152 to 5,869)	1,415 (992 to 1,874)	15,366 (10,877 to 20,209)	11,709 (8,311 to 15,536)	3,657 (2,627 to 4,833)	162.1 (135.3 to 185.5)	163.2 (128.7 to 193.4)	158.4 (140.4 to 178.7)
		Age-standardized rate (per 100,000)	15.8 (11.4 to 20.6)	24.4 (17.3 to 32.3)	7.6 (5.4 to 10)	17.3 (12.4 to 22.7)	26.4 (18.7 to 34.7)	8.3 (6 to 10.9)	9.8 (−3 to 20.5)	8.4 (−7.9 to 22)	10 (2.6 to 19.1)
	YLLs	All ages (number)	723 (405 to 1133)	544 (229–934)	179 (121–237)	2,058 (872 to 2,704)	1,490 (346 to 2,113)	567 (474 to 679)	184.7 (29.2 to 364.2)	173.9 (−22.1 to 443.4)	217.5 (126.5 to 412.8)
		Age-standardized rate (per 100,000)	2.4 (1.4 to 3.6)	3.7 (1.7 to 6.1)	1.1 (0.8 to 1.4)	2.7 (1.1 to 3.6)	4 (0.9 to 5.6)	1.5 (1.2 to 1.8)	14 (−50.9 to 88)	7.6 (−71.6 to 116)	31.7 (−4.8 to 101.8)
	YLDs	All ages (number)	5,140 (3,463 to 6,920)	3,904 (2,658 to 5,217)	1,236 (835 to 1,692)	13,308 (9,072 to 18,115)	10,219 (6,970 to 13,787)	3,090 (2,058 to 4,238)	158.9 (144.6 to 173.6)	161.8 (143.9 to 180.3)	149.9 (135.3 to 165)
		Age-standardized rate (per 100,000)	13.4 (9.0 to 18.0)	20.7 (14.0 to 27.8)	6.4 (4.4 to 8.9)	14.6 (9.9 to 19.8)	22.4 (15.2 to 30.2)	6.9 (4.6 to 9.4)	9.1 (4.2 to 14.3)	8.5 (2.6 to 15.1)	6.3 (1.0 to 12.0)
**Osteoarthritis**	Incidence	All ages (number)	129,719 (114,779 to 146,476)	69,896 (61,539 to 79,587)	59,823 (52,839 to 67,238)	385,983 (342,697 to 434,999)	217,254 (191,899 to 246,712)	168,729 (149,457 to 189,008)	197.6 (192.6 to 202.6)	210.8 (206.5 to 215.9)	182.0 (174.7 to 189.2)
		Age-standardized rate (per 100,000)	414.6 (369.2 to 466.7)	466.2 (411.6 to 529.4)	367.0 (327.5 to 411.7)	445.0 (395.9 to 501.9)	502.3 (444.0 to 570.1)	388.5 (346.6 to 435.0)	7.3 (6.4 to 8.4)	7.7 (6.5 to 9.2)	5.9 (4.6 to 7.0)
	Prevalence	All ages (number)	1,427,709 (1,276,694 to 1,606,168)	769,319 (684,581 to 871,511)	658,391 (586,468 to 736,831)	4,282,092 (3,844,615 to 4,781,751)	2,431,715 (2,173,730 to 2,731,630)	1,850,377 (1,662,706 to 2,060,557)	199.9 (196.7 to 204.0)	216.1 (211.6 to 220.8)	181.0 (176.7 to 186)
		Age-standardized rate (per 100,000)	5224.1 (4699.5 to 5821.4)	5916.4 (5303.0 to 6656.7)	4575.0 (4118.3 to 5069.3)	5588.2 (5041.2 to 6228.6)	6337.5 (5706.9 to 7098.7)	4836.2 (4354.1 to 5353)	7.0 (6.1 to 8.0)	7.1 (5.9 to 8.5)	5.7 (4.5 to 6.9)
	YLDs	All ages (number)	49,531 (25,023 to 97,062)	26,852 (13,574 to 52,638)	22,679 (11,378 to 44,498)	149,354 (75,490 to 293,270)	85,439 (43,376 to 167,896)	63,915 (31,778 to 126,546)	201.5 (197.8 to 206)	218.2 (213.3 to 223.8)	181.8 (176.9 to 187.2)
		Age-standardized rate (per 100,000)	181.0 (91.8 to 355.0)	206.5 (104.9 to 405.2)	157.0 (78.9 to 310.7)	195.3 (98.7 to 381.0)	223.3 (113.5 to 439.6)	167.1 (83.7 to 332.0)	7.9 (6.9 to 9.1)	8.1 (6.7 to 9.8)	6.5 (5.0 to 7.9)
**Low back pain**	Incidence	All ages (number)	1,564,670 (1,374,347 to 1,779,555)	721,857 (631,592 to 820,838)	842,813 (739,297 to 959,272)	3,050,709 (2,686,650 to 3,487,116)	1,469,294 (1,291,909 to 1,674,364)	1,581,416 (1,388,607 to 1,816,032)	95.0 (85.1 to 104.7)	103.5 (92.6 to 114.6)	87.6 (78.1 to 96.6)
		Age-standardized rate (per 100,000)	3,811.3 (3,375.5 to 4,288.0)	3,604.4 (3,186.8 to 4,052.6)	4,001.2 (3,544.9 to 4,522.4)	3,493.0 (3,092.5 to 3,949.5)	3,422.1 (3,024.5 to 3,862.1)	3,564.5 (3,148.3 to 4,039.6)	−8.4 (−9.2 to - 7.5)	−5.1 (−6.2 to - 3.9)	−10.9 (−12.0 to −9.7)
	Prevalence	All ages (number)	3,701,159 (3,239,976 to 4,181,658)	1,749,108 (1,530,130 to 1,984,434)	1,952,051 (1,711,467 to 2,207,239)	7,415,044 (6,524,554 to 8,393,689)	3,653,273 (3,219,190 to 4,130,287)	3,761,771 (3,310,136 to 4,259,099)	100.3 (91.3 to 108.5)	108.9 (98.4 to 118.3)	92.7 (84.1 to 100.7)
		Age-standardized rate (per 100,000)	9,377.0 (8,310.7 to 10,579.1)	9,089 (8,033.9 to 10,286.4)	9,633.4 (8,515.5 to 10,852.1)	8,486.5 (7,533.8 to 9,528.3)	8,508.6 (7,564.3 to 9,575.3)	8,470 (7,496.4 to 9,535.6)	−9.5 (−10.6 to −8.3)	−6.4 (−7.8 to - 4.8)	−12.1 (−13.6 to −10.4)
	YLDs	All ages (number)	418,229 (292,449 to 558,428)	195,476 (137,740 to 262,881)	222,753 (155,680 to 298,277)	837,779 (583,888 to 1,128,834)	408,084 (285,313 to 547,130)	429,694 (299,265 to 579,099)	100.3 (90.9 to 108.9)	108.8 (98.1 to 118.8)	92.9 (83.8 to 101.3)
		Age-standardized rate (per 100,000)	1051.4 (738.5 to 1404.5)	1007.7 (712.7 to 1340.1)	1091.4 (762.3 to 1464.1)	951.9 (668.9 to 1275.2)	944.1 (665.0 to 1258.7)	960.1 (674.7 to 1289.7)	−9.5 (−10.7 to −8.2)	−6.3 (−7.7 to - 4.7)	−12.0 (−13.7 to −10.4)

Data in parenthesis are 95% uncertainty intervals (UI); DALYs = Disability-Adjusted Life Years; YLLs = Years of Life Lost; YLDs = Years Lived with Disability.

**Fig 3:**
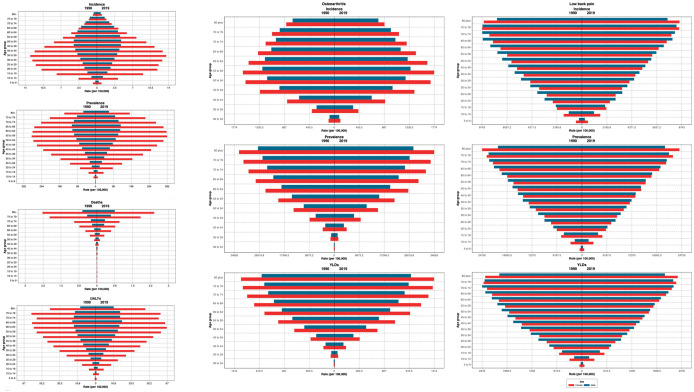
Epidemiological burden measures rate due to the RA (left), OA (middle), and LBP (right) in 1990 and 2019 by sex and age group.

**Fig 4:**
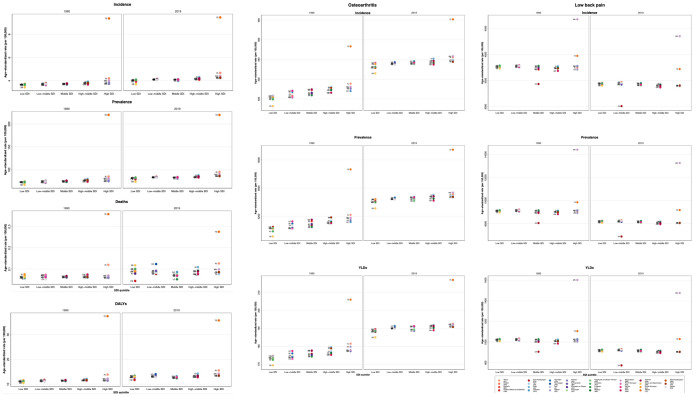
Subnational age-standardized epidemiological burden measures rate due to RA (left), OA (middle) and LBP (right) in 1990 and 2019 based on the socio-demographic index (SDI) quintiles.

Regarding the age groups, the highest incidence rate was observed in 35–39 years’ age group and the pattern was decreasing with older ages. The rate of incidence, prevalence and DALYs were higher in females in all age groups. The highest rate of prevalence and DALYs were in 55–64 age groups (Fig 3a). The ASIR and age-standardized death rate did not show significant differences between SDI quintiles and there was a mild increase in the ASIR of all provinces between 1990 and 2019. Tehran had the highest ASIR and age-standardized death rate in both 1990 and 2019 (Fig 4a).

Decomposition analysis of new cases at national level showed an overall 102.4% increase from 1990 to 2019, in which 12.9%, 45.5%, and 44.0% of the changes in new cases were attributable to incidence rate, age structure change, and population growth, respectively. This pattern was observed in both sexes (Table 2).

**Table 2:** Decomposition analysis of RA and OA new cases between 1990 and 2019 at the national level by sex.

Cause	Sex	New cases		Expected new cases in 2019		% 1990–2019 new cases change cause			% 1990 - 2019 new cases overall change
		1990	2019	Population growth	Population growth + Aging	Population growth	Age structure change	Incidence rate change	
**Rheumatoid arthritis**	Both	2,314	4,684	3,332	4,385	44.0%	45.5%	12.9%	102.4%
	Female	1,748	3,485	2,534	3,253	44.9%	41.1%	13.3%	99.3%
	Male	566	1,199	809	1,129	43.1%	56.5%	12.5%	112.0%
**Osteoarthritis**	Both	129,719	385,983	186,796	358,948	44.0%	132.7%	20.8%	197.6%
	Female	69,896	217,254	101,312	201,207	44.9%	142.9%	23.0%	210.8%
	Male	59,823	168,729	85,604	159,004	43.1%	122.7%	16.3%	182.0%

**Fig 5:**
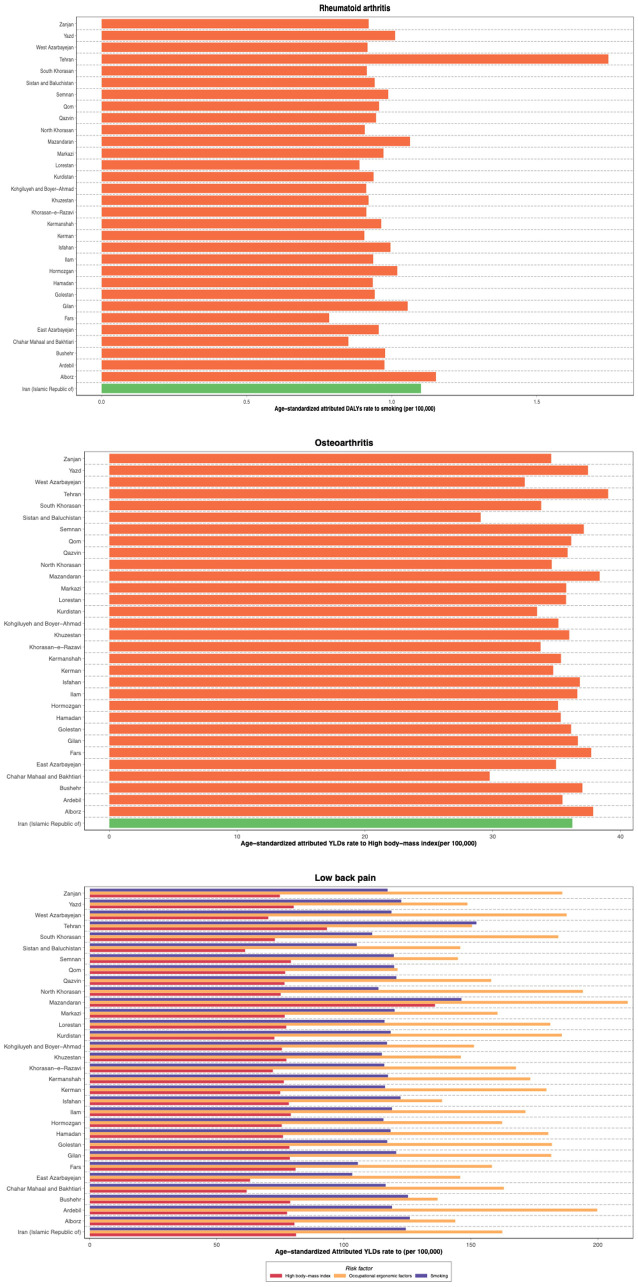
Age-standardized epidemiological burden measures rate due to RA (top), OA (middle), and LBP (bottom) attributed to risk factors in 2019.

The age-standardized DALYs rate attributed to smoking have decreased 4.1% between 1990 and 2019. In 2019, Tehran had the highest and Fars had the lowest age-standardized DALYs rate attributed to smoking. In most of the provinces the attributed rate was lower that national rate (Table 3, Fig 5a).

**Table 3:** National all-ages (number) and age-standardized rate (per 100,000) burden due to RA, OA, and LBP attributed to risk factors by sex.

Cause	Risk factor	Age (metric)	Measure	Year						% Change (1990–2019)		
				1990			2019					
				Both	Female	Male	Both	Female	Male	Both	Female	Male
**Rheumatoid arthritis**	Smoking	All ages (number)	Deaths	2 (1 to 3)	1 (0 to 1)	1 (0 to 2)	5 (1 to 10)	2 (0 to 4)	4 (1 to 6)	199.4 (95.6 to 324.1)	166.9 (−26.7 to 522.7)	216.1 (114.1 to 397.9)
		Age-standardized rate (per 100,000)		0.01 (0 to 0.01)	0 (0 to 0.01)	0.01 (0 to 0.02)	0.01 (0 to 0)	0 (0 to 0.01)	0.01 (0 to 0.02)	9.4 (−28.2 to 53.6)	−7.2 (−74.2 to 115.5)	17.7 (−18 to 79.3)
		All ages (number)	DALYs	384 (115 to 723)	130 (32 to 275)	254 (78 to 471)	997 (279 to 1882)	346 (75 to 725)	651 (192 to 1177)	159.2 (108.3 to 208.2)	166.2 (81.9 to 285.8)	155.7 (108.4 to 196.1)
		Age-standardized rate (per 100,000)		1.15 (0.35 to 2.17)	0.8 (0.2 to 1.71)	1.47 (0.45 to 2.72)	1.1 (0.31 to 2.1)	0.76 (0.17 to 1.58)	1.43 (0.43 to 2.59)	−4.1 (−22.8 to 14.1)	−4.7 (−35.3 to 37.8)	−2.6 (−20.3 to 13.2)
		All ages (number)	YLLs	51 (15 to 96)	17 (4 to 44)	34 (10 to 65)	146 (42 to 271)	44 (8 to 102)	102 (32 to 173)	186.6 (87.1 to 315.1)	163 (−25.1 to 513.3)	198.1 (99.6 to 384.1)
		Age-standardized rate (per 100,000)		0.17 (0.05 to 0.32)	0.12 (0.03 to 0.29)	0.22 (0.06 to 0.4)	0.18 (0.05 to 0.3)	0.11 (0.02 to 0.25)	0.25 (0.08 to 0.43)	7.8 (−29.8 to 54.4)	−5.8 (−73.5 to 117.2)	15.9 (−21 to 83.6)
		All ages (number)	YLDs	334 (100–633)	113 (27 to 244)	220 (65 –412)	851 (219–1648)	302 (66 –622)	549 (160 to 1021)	155 (105.6 to 201.1)	166.6 (86 to 281.7)	149.1 (100.1 to 184.6)
		Age-standardized rate (per 100,000)		0.98 (0.29 to 1.86)	0.69 (0.16 to 1.45)	1.26 (0.37 to 2.34)	0.92 (0.24 to 1.8)	0.66 (0.14 to 1.34)	1.18 (0.34 to 2.21)	−6.1 (−24 to 11.2)	−4.5 (−33.6 to 35.5)	−5.7 (−23.9 to 7.9)
**Osteoarthritis**	High body- mass index	All ages (number)	YLDs	6,197 (2,248 to 13,669)	3,794 (1,327 to 8,749)	2,403 (778 to 5,513)	28,105 (11,410 to 62,108)	16,444 (6,289 to 37,874)	11,661 (4,306 to 25,408)	353.5 (295.2 to 461)	333.4 (285.3 to 429.2)	385.3 (296 to 586.7)
		Age-standardized rate (per 100,000)		21.7 (7.9 to 48.4)	28 (9.8 to 64.5)	15.9 (5.2 to 36.8)	36.2 (14.9 to 79.9)	42.5 (16.3 to 97.4)	29.9 (11 to 64.9)	67 (45.7 to 107.6)	51.8 (34.6 to 86.1)	87.8 (54.4 to 167.8)
**Low back pain**	High body- mass index	All ages (number)	YLDs	19,329 (9,336 to 33,751)	10,890 (5,525 to 18,245)	8,439 (3,636 to 15,421)	73,084 (40,187 to 118,045)	38,220 (21,398 to 60,997)	34,864 (18,424 to 57,738)	278.1 (226 to 386.6)	251.0 (206.1 to 334.2)	313.1 (238.5 to 491.0)
		Age-standardized (rate per 100,000)		54.0 (27.0 to 94.0)	63.8 (32.7 to 108.5)	45.4 (20.4 to 82.0)	81.2 (45.0 to 131.7)	86.3 (48.8 to 136.4)	76.1 (40.5 to 124.9)	49.4 (30.8 to 86.6)	35.2 (19.7 to 64.3)	67.6 (39.4 to 138.0)
	Occupational ergonomic factors	All ages (number)		79,635 (53,251 to 108,679)	10,918 (6,863 to 16,148)	68,717 (45,089 to 94,660)	158,433 (106,920 to 216,036)	32,050 (21,062 to 45,176)	126,383 (84,816 to 173,153)	98.9 (75.9 to 124.7)	193.6 (120.5 to 298.9)	83.9 (61.6 to 108.7)
		Age-standardized (rate per 100,000)		196.5 (132.7 to 268.3)	55.5 (34.8 to 82.4)	329.6 (222.3 to 448.5)	162.4 (110.1 to 222.0)	65.4 (43.4 to 92.0)	257.3 (174.6 to 353.1)	−17.4 (−26.3 to −7.7)	17.8 (−12.3 to 62.1)	−21.9 (−30.5 to −11.7)
	Smoking	All ages (number)		52,723 (32,465 to 75,156)	7,661 (4,541 to 11,659)	45,062 (27,568 to 64,346)	111,823 (69,869 to 160,221)	18,016 (10,984 to 26,810)	93,808 (58,311 to 135,662)	112.1 (95.7 to 132.8)	135.2 (79.3 to 212.0)	108.2 (91.4 to 128.3)
		Age-standardized (rate per 100,000)		159.0 (98.0 to 227.9)	48.8 (28.7 to 74.1)	262.4 (161.1 to 375.4)	124.4 (78.4 to 177.7)	41.1 (25.2 to 60.9)	206.9 (129.6 to 296.7)	−21.8 (−27.8 to −14.9)	−15.8 (−35.5 to 10.5)	−21.1 (−26.9 to −14.5)

Data in parenthesis are 95% uncertainty intervals (UI); DALYs = Disability-Adjusted Life Years; YLLs = Years of Life Lost; YLDs = Years Lived with Disability.

### 3.2. Osteoarthritis (OA)

The OA new cases increased from 129,719 (114,779–146,476) in 1990–385,983 (342,697–434,999) in 2019 and the percentage of increase was higher in females compared to males (210.8% and 182.0%, respectively). In 2019, the absolute number of OA cases in Iran were 4,282,092 (3,844,615–4,781,751) and were higher in females compared to males (female: 2,431,715, male: 1,850,377). The ASIR of OA has increased by 7.3% from 414.6 (369.2 to 466.7) in 1990 to 445.0 (395.9 to 501.9) in 2019 per 100,000 population and the increase was higher in females (7.7%) compared to males (5.9%). The ASPR in 2019, was 5588.2 (5041.2 to 6228.6) and increased by 7.1% and 5.7% in females and males, respectively. The number of OA YLDs in 2019 were 149,354 (75,490–293,270) and had a 201.5% increase between 1990 and 2019. The ASYR had a 7.9% increase during this period (Table 1, Figs 1b and 2b).

At the subnational level, the lowest and highest increase in the ASIR, ASPR, and ASYR were reported for Tehran (6.0%, 4.8%, 5.3%) and Ilam (8.9%, 8.8%, 9.7%), respectively. The highest increase for ASYR was also reported for Lorestan (9.7%) (S3 Table).

With regard to the age groups, the highest and lowest incidence rate was seen in 55–59 years and 30–34 years old, respectively. The incidence rate showed a decreasing pattern by increasing in age after 55–59 years. Meanwhile, the highest rate of prevalence and YLDs was in 80 years and older. Females had higher rates of incidence, prevalence, and YLDs in all age groups compared to males (Fig 3b).

The ASIR and ASYR were higher in provinces with higher SDI and increased in all SDIs between 1990 and 2019. The increase was more prominent in low SDI provinces such as Sistan and Baluchistan and Kurdistan. Tehran had the highest ASIR and ASYR among all provinces both in 1990 and 2019 (Fig 4b).

Decomposition analysis of new cases at the national level showed an overall 197.6% increase from 1990 to 2019, in which 20.8%, 132.7%, and 44.0% of the changes in new cases were due to incidence rate, age structure change, and population growth, respectively. This pattern was observed in both sexes (Table 2).

The ASYR attributed to high BMI has increased from 21.7 (7.9 to 48.4) in 1990 to 36.2 (14.9 to 79.9) in 2019 by 67.0% which has been more in males (87.8%) than females (51.8%). The highest ASYR attributed to high BMI was in Tehran and Mazandaran and the lowest was observed in Sistan and Baluchistan and Chahar Mahaal and Bakhtiari (Table 3, Fig 5b).

### 3.3. Low back pain (LBP)

The number of LBP new cases increased from 1,564,670 (1,374,347–1,779,555) in 1990–3,050,709 (2,686,650–3,487,116) in 2019. However, the ASIR decreased by −8.4% during this period with a higher decrease in males (10.9%) compared to females (5.1%). In 2019, the number of LBP cases was 7,415,044 (6,524,554–8,393,689) and was higher in males compared to females (females: 3,653,273; males: 3,761,771). The ASPR and ASYR have decreased by 9.5% and 9.5%, respectively during 1990–2019 (Table 1, Figs 1c and 2c).

At the subnational level, the ASIR, ASPR, and ASYR decreased in all provinces between 1990 and 2019. The highest decreases of ASIR, ASPR, and ASYR were in East Azarbayejan (−12.4%, −14.5%, and −14.6%, respectively), and the lowest decreases were in Tehran (ASIR: −6.4%, ASPR: −6.9%, and ASYR: −6.9%, respectively) (S4 Table).

Regarding the age groups, the highest and lowest incidence, prevalence, and YLDs rate were seen in 75–79 years and 5–9 years, respectively. These rates showed an increasing pattern by increasing in age. Males had higher rates of incidence, prevalence, and YLDs rates in almost all age groups compared to females except for 5–14 years and also 80-plus age groups (Fig 3c). The ASIR and ASYR did not show significant differences between SDI quintiles and there was a decrease in the ASIR and ASYR rates of all provinces between 1990 and 2019. Mazandaran and Tehran had the highest, and East Azarbayejan had the lowest ASIR and ASYR in both 1990 and 2019 (Fig 4c).

Decomposition analysis of new cases at the national level showed an overall 95% increase from 1990 to 2019, in which −18.8%, 69.8%, and 44.0% of the changes in new cases were due to incidence rate, age structure change, and population growth, respectively. This pattern was observed in both sexes.

The ASYR attributed to high BMI has increased by 49.4%, while it has decreased for smoking (−21.8%) and occupational ergonomic factors (−17.4%) between 1990 and 2019. In 2019, Mazandaran had the highest and Qom had the lowest ASYRs attributed to occupational ergonomic factors. Tehran and Mazandaran had the highest ASYRs attributed to smoking. Also, Mazandaran had the highest ASYRs attributed to high BMI (Table 3, Fig 5c).

## 4. Discussion

Communicable diseases, maternal diseases, neonatal diseases, and malnutrition conditions are shifting toward non-communicable diseases (NCDs) and have attracted much attention in recent decades. The NCDs comprised 43.9% of all-caused global DALYs in 1990, whereas they steadily increased during past decades and reached 61.4% in 2016 [31]. Due to the life expectancy and rising average age of people worldwide, people are more prone to developing NCDs like MSDs [32]. Moreover, obesity and smoking are well-known risk factors for the development of MSD [33,34], and due to the increasing pattern of obesity and smoking in Iranian individuals, an increase in the incidence of MSDs is expected [35,36].

Mounting evidence points toward the high prevalence and burdensome nature of MSDs around the world, and all MSDs comprise 17.12% of all YLDs, and 5.896% of all caused DALY [37]. To the best of our knowledge, this is the first epidemiological study that reported the absolute number, rate, trend, and attributed risk factors of OA, RA, and LBP in Iran at the subnational level. Our study was designed to inform the national and subnational levels of health parameters: prevalence, incidence, death, DALYs, YLLs, and YLDs by all ages number, age-standardized rates, and by the age groups and sex for the selected disorders of MSDs.

OA is a common joint disorder, contributing significantly to disability and activity limitations. Its prevalence is steadily rising due to the aging global population. Risk factors for OA include female gender, older age, genetic susceptibility, obesity, and joint injury [38]. Our study shows that the incidence of OA increased from 1990 to 2019, with a higher rise in females than in males. The age-standardized incidence rate (ASIR) of OA increased by 7.3%, with females showing a higher increase (7.1%) compared to males (5.7%). YLDs for OA rose by 201% between 1990 and 2019. Females had higher rates of incidence, prevalence, and YLDs in all age groups, indicating female gender as a risk factor. Previous studies confirm a higher OA burden in females [39,40], which is likely multifactorial, involving hormonal, genetic, and structural factors [41].

Age group analysis revealed that individuals aged 55–59 had the highest incidence of OA, while those aged 30–34 had the lowest. Although higher age correlates with greater incidence, a decline was observed in those over 59. The highest prevalence and YLDs were seen in patients over 80, confirming aging as a significant risk factor. A higher incidence in females aged 40–60 and a decreasing trend after 60 aligns with other studies [42,43]. On a subnational scale, Tehran and Ilam provinces showed the lowest and highest increases in age-standardized incidence rate (ASIR), age-standardized prevalence rate (ASPR), and age-standardized YLDs, respectively, while Lorestan had the highest increase in age-standardized years lived with disability (ASYR). These findings can inform health policy and resource allocation to better manage OA’s burden.

According to the SDI, ASIR and ASYR increased across all SDIs from 1990 to 2019, with higher SDI provinces showing higher rates. Tehran, as a high-SDI province, had the highest ASIR and ASYR in both 1990 and 2019. However, the increase was more pronounced in low-SDI provinces, highlighting the economic impact on the OA burden. Obesity or high BMI is a well-established risk factor for OA. Our study found that the ASYR attributed to high BMI increased by 67.0% from 1990 to 2019, with a higher increase in males than females.

OA remains a significant global public health issue. MSDs account for 17.11% (14.86 to 19.78) of all YLDs, with OA being the third most common cause of YLDs among MSDs, following low back pain (7.41% (6.16 to 8.74)) and neck pain (2.56% (1.98 to 3.32)). OA contributed 2.19% (1.29 to 4.07) of all YLDs in 2019 [37]. Our findings align with the GBD 2017 study, which showed an increasing trend in the OA burden globally [44]. Fu et al. focused on hip OA, noting rising incidence and YLDs worldwide [45]. Liu et al. documented a significant increase in the burden of knee and hip OA in the USA and China [46]. A GBD 2019 study on hand OA, the most common form, found an 82.07% increase in incidence from 1990 to 2019, though age-standardized incidence and years lived with disability (ASIR and ASYR) rates declined. This decrease may reflect improved management of OA [43].

RA is the most common inflammatory joint disease, leading to significant morbidity, disability, and socioeconomic burden [47]. Advances in understanding RA’s pathophysiology have improved patient prognosis. Risk factors for RA are divided into host factors (e.g., smoking, diet, infections, lifestyle, socioeconomic factors, airborne agents) and intrinsic factors (e.g., genetics, epigenetics, hormones, reproductive, neuropsychiatric, and neuroendocrine factors) [48]. RA is a major global health issue, with higher prevalence in North America, Europe, and the Eastern Mediterranean regions [49]. The GBD 2017 study reported a prevalence of 20 million, an incidence of 1.2 million, and DALYs of 3.4 million for RA, compared to 4.8 million DALYs in GBD 2010, despite similar prevalence figures [50,51]. The GBD 2017 also showed an increase in age-standardized incidence and prevalence rates (ASIR and ASPR) from 1990 to 2017, while age-standardized DALYs declined [51]. The National and Subnational Burden of Diseases, Injuries, and Risk Factors (NASBOD) study on the Iranian population documented that the rate of RA death increased from 1990 to 2015 (99.46 to 247.81 per 100,000 from 1990 to 2015, respectively) [52]. Our study aimed to examine the epidemiological data for RA in Iran and at the subnational level. The absolute number of RA patients increased more than 29-fold by 2019 compared to 1990, with a 12.9% rise in incidence rate. Additionally, the ASIR of RA increased by 7.6% in 2019 compared to 1990, confirming the upward trend observed in previous studies.

Hormonal and sex-related factors are recognized as risk factors contributing to the sex imbalance in RA, with female predisposition linked to the inflammatory effects of estrogen [53]. Our findings showed that females are more susceptible to developing RA than males. The age-standardized prevalence rate (ASPR) and age-standardized years lived with disability (ASYR) both increased by 9.3% and 9.1%, respectively, with higher rates in females. The highest incidence rate was observed in those aged 35–39, with a decline in older age groups. Incidence, prevalence, and DALYs were higher in females across all age groups, with the highest prevalence and DALYs in the 55–64 age group. The GBD 2017 study also reported higher age-standardized DALYs, ASIR, and ASPR in females compared to males, with the highest prevalence in females aged 70–74 and males aged 75–79 [51].

The subnational analysis revealed that Tehran and Ilam provinces had the lowest and highest increases in ASIR, ASPR, and ASYR from 1990 to 2019, respectively. According to the SDI, there were no significant differences in ASIR and age-standardized death rates across SDI quintiles, with a mild increase in ASIR across all provinces between 1990 and 2019. Tehran had the highest ASIR and age-standardized death rate in both 1990 and 2019.

Several airborne noxious agents have been shown to play a role in the development of RA, and smoking has long been considered a risk factor for the establishment of RA [54]. It was estimated that smoking is considered a 20–25% risk for RA [55]. The age-standardized DALYs rate attributed to smoking has decreased by 4.1% between 1990 and 2019. In 2019, Tehran had the highest and Fars had the lowest age-standardized DALYs rate attributed to smoking. Prevention of risk factors is an appropriate approach for the management of diseases. Therefore, controlling smoking and tobacco is associated with the success of RA management [56,57]. Prior studies also showed a 28.4% and 34.4% decrease in the ASPR of daily smoking from 1990 in males and females, respectively [58].

As another musculoskeletal disorder, LBP is a frequent complaint of people around the world in every age group, both sexes, and in developed and developing countries. Almost everyone experiences at least one episode of LBP during their lifetime, which can significantly impact their quality of life [59]. LBP imposes a high burden on patients, healthcare providers, and governments, with a high prevalence. In 2017, LBP was the leading cause of YLDs among chronic diseases [60]. LBP accounted for 7.4% of global YLDs and 2.5% of DALYs [37]. GBD 2019 reported a slight decline in the age-standardized prevalence rate (ASPR), incidence rate (ASIR), and years lived with disability rate (ASYR) for LBP over recent decades, though the absolute numbers of prevalent cases, incidents, and YLDs have significantly increased, likely due to global lifestyle changes [61]. LBP remains the leading cause of YLDs worldwide, with rising burdens observed in countries such as Zambia, Mali, and Canada [61].

Our study showed a 95% increase in the absolute number of LBP cases from 1990 to 2019. While LBP rates were higher in males, the age-standardized incidence rate (ASIR) decreased more in males than in females. Decomposition analysis revealed that, despite the increase in LBP cases, the ASIR declined in both sexes. This decline may reflect improvements in workplace ergonomics, as occupational health policies and industrial safety regulations have evolved in Iran over the past decades. Additionally, greater public awareness about proper posture, physical activity, and back health may have encouraged earlier management of back discomfort, preventing progression to chronic LBP [62]. Males had higher rates of incidence, prevalence, and YLDs in most age groups, except for those aged 5–14 and over 80 years. The 2017 and 2019 GBD studies found females to be more susceptible to LBP, likely due to menopausal changes and sex hormones [61,63]. Besides, the ASPR and ASYR have decreased by 9.5% and 9.5%, respectively, from 1990 to 2019.

Regarding the age groups, the highest incidence, prevalence, and YLDs rate were observed in 75–79 years, which showed an increasing pattern as well as an increase in age. GBD 2019 study also revealed that the incidence, prevalence, and YLDs rate are growing by age, with the greatest being in patients aged between 80 and 84 years. Additionally, the absolute number of prevalent, incident, and YLDs were summiting 45–54 years for both sexes [61]. Some factors were recognized that are responsible for the age pattern of patients. Disc degeneration, a major risk factor for LBP, is strongly correlated with aging [64,65]. Subnational analysis showed a decrease in ASIR, ASPR, and ASYR across all provinces, with the greatest reduction in East Azarbayjan and the smallest in Tehran. Several recognized risk factors for LBP include abdominal obesity, smoking, female gender, and physical workload [66]. Between 1990 and 2019, ASYR attributed to high BMI increased, while those linked to smoking and occupational ergonomic factors decreased. In 2019, Mazandaran had the highest and Qom the lowest ASYR attributed to occupational ergonomic factors, while Tehran and Mazandaran had the highest ASYR attributed to smoking.

### 4.1. Limitations and suggestions

This study faced some drawbacks. Like other GBD articles, data availability and uncertainty of data are major challenges. The GBD data relies on modeling and estimation based on primary data, which can introduce uncertainty. Additionally, due to the lack of strong registration systems at the subnational level, accurate and certain data may not be available for every location. While the modeling approach aims to provide the most accurate estimates, the absence of robust primary data at local levels limits the reliability of the findings in some regions. More primary data is needed to improve the accuracy of the epidemiological estimates and to better inform healthcare policies. Future research could focus on improving data collection methods at the subnational level to address these gaps.

Another limitation is that the study primarily focused on OA, RA, and LBP, and further investigation into the distribution of their subtypes could provide more detailed data, which would aid governments and health institutes in making more informed decisions. Finally, we did not apply advanced data mining or predictive modeling approaches, such as clustering or classification and regression trees, which could provide additional insights into patterns or future projections of disease burden. These methods were not implemented because our study was based on finalized GBD estimates, which follow a standardized modeling framework to ensure comparability across locations and over time. Future research using primary data sources or raw GBD inputs could apply these methods to complement GBD findings and explore predictive scenarios.

In terms of public health policy, several actions could be taken to reduce the burden of these MSDs. First, given the significant role of obesity in the development of OA, RA, and LBP, public health interventions targeting BMI reduction are crucial. Programs promoting weight management, healthier diets, and increased physical activity could have a significant impact on the reduction of these conditions. Second, smoking has been identified as a key risk factor, and public health initiatives focusing on smoking cessation are vital. These could include awareness campaigns, stricter tobacco control measures, and smoking cessation programs. Lastly, to improve the accuracy of future research, it is essential to enhance data collection at the subnational level. Strengthening health data registration systems would ensure more reliable and region-specific data, which would be invaluable for better health policy planning and resource allocation.

## 5. Conclusion

MSDs are among the disorders with a high burden in Iran, showing different geographical distribution. Our study found an increase in the total number of LBP, RA, and OA cases, with a rise in the incidence of OA and RA, but not LBP. The sex distribution of RA and OA shows a female predisposition, while males are more prone to developing LBP. The ASIR, ASPR, and age-standardized YLDs of OA and RA have increased, while those of LBP have decreased. Increasing awareness of the epidemiological data of RA, OA, and LBP, along with their geographical distribution, will provide better insights for governments and health institutes to formulate more appropriate health policies, with a particular focus on addressing the rising burden of RA and OA and strategies for reducing LBP prevalence.

## Supporting information

S1 Table List of International Classification of Diseases (ICD) codes mapped to the Global Burden of Disease cause list. (PDF)

S2 Table Age-standardized rate of incidence, prevalence, death, disability-adjusted life years (DALYs), years lived with disability (YLDs), and years of life lost (YLLs) of RA in 1990 and 2019. (PDF)

S3 Table All ages number and age-standardized rate of incidence, prevalence, and YLDs of OA in 1990 and 2019. (PDF)

S4 Table All ages number and age-standardized rate of incidence, prevalence, and YLDs of LBP in 1990 and 2019. (PDF)
