# Supplementary material for: National and subnational burden and attributable risk factors of osteoarthritis, rheumatoid arthritis, and low back pain in Iran: 1990–2019 findings of the Global Burden of Disease (GBD) Study
Source: PLoS One. 2026 Jul 6;21(7):e0344038. doi: 10.1371/journal.pone.0344038 (PMC13336167; doi:10.1371/journal.pone.0344038)
Supplement: S1 Table — (PDF) [file pone.0344038.s001.pdf]

S1 Table. List of International Classification of Diseases (ICD) codes mapped to the Global Burden of Disease cause list

Cause	Hospital/claim analysis	Death analysis
Rheumatoid arthritis	M05-M05.9, M08-M09.8	M05-M06.9, M08.0-M08.8
Osteoarthritis	M16-M18.9	
Osteoarthritis hip	M16-M16.9	
Osteoarthritis knee	M17-M17.9	
Osteoarthritis hand	M18-M18.9	
Osteoarthritis other		
Low back pain	G54.4, M47.015-M47.019, M47.15-M47.18, M47.25-M47.28, M47.815-M47.818, M47.896-M47.899, M48.05-M48.08, M48.16-M48.19, M48.25-M48.27, M48.35-M48.38, M48.45-M48.48, M48.55-M48.58, M49.85-M49.88, M51.05-M51.07, M51.15-M51.17, M51.25-M51.27, M51.35-M51.37, M51.45-M51.47, M51.85-M51.87, M53.3, M53.85-M53.88, M54.05-M54.09, M54.15-M54.18, M54.3-M54.5, M99.03-M99.04, M99.13-M99.14, M99.23-M99.24, M99.33-M99.34, M99.43-M99.44, M99.53-M99.54, M99.63-M99.64, M99.73-M99.74, M99.83-M99.84	
