# Supplementary material for: National and subnational burden and attributable risk factors of osteoarthritis, rheumatoid arthritis, and low back pain in Iran: 1990–2019 findings of the Global Burden of Disease (GBD) Study
Source: PLoS One. 2026 Jul 6;21(7):e0344038. doi: 10.1371/journal.pone.0344038 (PMC13336167; doi:10.1371/journal.pone.0344038)
Supplement: S2 Table — (PDF) [file pone.0344038.s002.pdf]

S2 Table. Age-standardized rate of incidence, prevalence, death, disability-adjusted life years (DALYs), years lived with disability (YLDs), and years of life lost (YLLs) of RA in 1990 and 2019

Province	Measure	Age-standardized rate (per 100,000)						% Change (1990 to 2019)		
		1990			2019					
		Both	Female	Male	Both	Female	Male	Both	Female	Male
Alborz	Incidence	4.21 (3.72 to 4.73)	6.02 (5.33 to 6.79)	2.57 (2.26 to 2.91)	4.67 (4.16 to 5.29)	6.65 (5.91 to 7.55)	2.76 (2.44 to 3.12)	11.1 (6.3 to 16.2)	10.5 (4.3 to 17.2)	7.5 (2.1 to 13.2)
	Prevalence	85.33 (75.79 to 95.63)	125.58 (111.16 to 140.78)	49.88 (43.78 to 56.59)	95.3 (84.51 to 107.4)	138.55 (122.74 to 156.28)	53.52 (47.06 to 60.69)	11.7 (6.9 to 17)	10.3 (4.3 to 16.8)	7.3 (2 to 13.4)
	Deaths	0.12 (0.06 to 0.2)	0.17 (0.07 to 0.32)	0.06 (0.04 to 0.09)	0.13 (0.06 to 0.18)	0.18 (0.04 to 0.29)	0.08 (0.05 to 0.11)	5.4 (-41.9 to 89.2)	4.6 (-63.9 to 137.6)	20.1 (-26.7 to 111.8)
	DALYs	13.84 (9.73 to 18.25)	20.08 (13.87 to 26.93)	8.26 (5.78 to 11.06)	15.52 (11.05 to 20.62)	22.2 (15.41 to 30.01)	9.09 (6.48 to 12.17)	12.2 (-4.5 to 30.3)	10.6 (-10.5 to 35)	10.1 (-6.3 to 30.4)
	YLLs	2.4 (1.31 to 3.81)	3.49 (1.34 to 6.27)	1.37 (0.82 to 1.98)	2.71 (1.2 to 3.79)	3.76 (0.88 to 5.77)	1.73 (1.23 to 2.39)	13 (-36 to 103.2)	7.9 (-61.7 to 142.8)	26.7 (-23.5 to 130.1)
	YLDs	11.44 (7.56 to 15.68)	16.59 (10.9 to 22.81)	6.89 (4.52 to 9.76)	12.81 (8.64 to 17.81)	18.44 (12.3 to 25.51)	7.36 (4.8 to 10.42)	12 (-2.4 to 27.6)	11.1 (-6.6 to 31.4)	6.8 (-10.5 to 26.3)
Ardebil	Incidence	3.8 (3.35 to 4.29)	5.26 (4.63 to 5.95)	2.35 (2.07 to 2.68)	4.16 (3.68 to 4.7)	5.89 (5.2 to 6.69)	2.55 (2.24 to 2.88)	9.6 (4.9 to 14.5)	12 (5.9 to 18.8)	8.5 (3.4 to 14.6)
	Prevalence	76.17 (67.41 to 85.52)	108.93 (96.19 to 122.99)	45.27 (39.9 to 51.56)	85.06 (75.52 to 95.73)	121.9 (108.18 to 137.95)	49.13 (43.28 to 55.51)	11.7 (6.9 to 16.8)	11.9 (5.6 to 18.7)	8.5 (2.9 to 15.1)
	Deaths	0.07 (0.04 to 0.13)	0.12 (0.05 to 0.25)	0.03 (0.02 to 0.05)	0.09 (0.05 to 0.12)	0.11 (0.05 to 0.16)	0.06 (0.05 to 0.09)	23.8 (-46 to 134.2)	-1.9 (-68.8 to 150.2)	90.7 (17.2 to 249.9)
	DALYs	11.79 (8.29 to 15.71)	16.91 (11.62 to 22.86)	7.03 (4.82 to 9.64)	13.3 (9.46 to 17.71)	18.57 (13.01 to 25.22)	8.13 (5.7 to 10.84)	12.8 (-4.9 to 30.2)	9.9 (-14 to 34.1)	15.6 (-2.6 to 37.7)
	YLLs	1.5 (0.88 to 2.93)	2.43 (1.11 to 5.61)	0.71 (0.45 to 1.06)	1.87 (1.1 to 2.39)	2.39 (1 to 3.29)	1.32 (0.96 to 1.79)	24.5 (-46.4 to 128.1)	-1.8 (-68 to 137.5)	86.7 (13.2 to 247.1)
	YLDs	10.29 (6.86 to 14.06)	14.47 (9.6 to 20.15)	6.33 (4.2 to 8.98)	11.43 (7.61 to 16.01)	16.18 (10.73 to 22.92)	6.81 (4.42 to 9.59)	11.1 (-2.5 to 26.7)	11.8 (-6.9 to 34.5)	7.6 (-9.3 to 27.8)

Province	Measure	Age-standardized rate (per 100,000)						% Change (1990 to 2019)		
		1990			2019					
		Both	Female	Male	Both	Female	Male	Both	Female	Male
Bushehr	Incidence	3.77 (3.34 to 4.25)	5.21 (4.62 to 5.9)	2.35 (2.07 to 2.67)	4.08 (3.61 to 4.61)	5.85 (5.19 to 6.64)	2.55 (2.23 to 2.91)	8.5 (3.7 to 12.7)	12.2 (6.1 to 18.8)	8.9 (3.7 to 14.8)
	Prevalence	76.07 (67.7 to 85.96)	108.11 (95.36 to 121.82)	45.3 (39.85 to 51.81)	83.52 (74.13 to 94.37)	121.4 (107.53 to 137.07)	49.46 (43.4 to 56.28)	9.8 (4.9 to 14.5)	12.3 (6 to 19.5)	9.2 (3.7 to 14.5)
	Deaths	0.06 (0.03 to 0.17)	0.1 (0.04 to 0.31)	0.02 (0.02 to 0.03)	0.07 (0.04 to 0.1)	0.1 (0.04 to 0.14)	0.05 (0.03 to 0.06)	20.9 (-66.3 to 190.6)	2.5 (-79.3 to 236)	98.9 (28.5 to 254.4)
	DALYs	11.52 (8.01 to 15.77)	16.41 (11.26 to 22.92)	6.82 (4.67 to 9.46)	12.79 (9 to 17.22)	18.31 (12.69 to 25.14)	7.78 (5.57 to 10.46)	11 (-10.1 to 30.4)	11.6 (-17.1 to 37.9)	14.1 (-2.4 to 37)
	YLLs	1.24 (0.59 to 3.44)	2.01 (0.75 to 6.49)	0.5 (0.31 to 0.73)	1.54 (0.89 to 1.96)	2.12 (0.87 to 2.92)	0.95 (0.71 to 1.28)	23.7 (-63.7 to 184.4)	5.3 (-77.7 to 227.4)	91.8 (21.6 to 231.7)
	YLDs	10.28 (7.01 to 14.24)	14.39 (9.8 to 20.46)	6.32 (4.2 to 8.82)	11.25 (7.47 to 15.67)	16.19 (10.64 to 23.08)	6.83 (4.58 to 9.5)	9.5 (-4.4 to 26.5)	12.5 (-6.5 to 35.8)	8.1 (-8.2 to 30.7)
Chahar Mahaal and Bakhtiari	Incidence	3.64 (3.23 to 4.09)	5.03 (4.44 to 5.65)	2.29 (2.02 to 2.62)	3.93 (3.49 to 4.42)	5.49 (4.84 to 6.22)	2.44 (2.14 to 2.77)	8 (3.6 to 12.9)	9.3 (3.7 to 16)	6.4 (1.1 to 12.5)
	Prevalence	72.92 (64.68 to 81.7)	103.94 (91.83 to 116.71)	44.09 (38.7 to 49.97)	79.63 (70.71 to 89.39)	113.29 (100.36 to 127.07)	46.84 (41.23 to 53.04)	9.2 (4.4 to 14.2)	9 (2.9 to 15.5)	6.2 (0.8 to 12.4)
	Deaths	0.06 (0.03 to 0.16)	0.09 (0.04 to 0.3)	0.02 (0.01 to 0.04)	0.04 (0.03 to 0.07)	0.06 (0.04 to 0.11)	0.03 (0.02 to 0.04)	-24.1 (-61.6 to 58.1)	-34.5 (-69.3 to 80.4)	10.6 (-33.8 to 91.1)
	DALYs	11 (7.69 to 15.13)	15.7 (10.77 to 22.01)	6.65 (4.49 to 9.11)	11.69 (8.25 to 15.9)	16.42 (11.52 to 22.48)	7.07 (4.84 to 9.81)	6.2 (-10.6 to 23)	4.6 (-17.2 to 28)	6.3 (-9.2 to 26)
	YLLs	1.15 (0.61 to 3.25)	1.85 (0.76 to 6.17)	0.51 (0.31 to 0.78)	0.91 (0.64 to 1.5)	1.26 (0.78 to 2.46)	0.55 (0.36 to 0.81)	-20.6 (-57.6 to 57.6)	-31.8 (-66 to 77.1)	8.6 (-36 to 95.4)
	YLDs	9.86 (6.79 to 13.82)	13.85 (9.39 to 19.49)	6.15 (4.01 to 8.64)	10.78 (7.23 to 14.98)	15.16 (10.37 to 21.38)	6.52 (4.27 to 9.25)	9.4 (-5.1 to 25.9)	9.5 (-9.4 to 30.9)	6.1 (-9.8 to 24.7)

Province	Measure	Age-standardized rate (per 100,000)						% Change (1990 to 2019)		
		1990			2019					
		Both	Female	Male	Both	Female	Male	Both	Female	Male
East Azarbayejan	Incidence	3.75 (3.33 to 4.23)	5.18 (4.57 to 5.87)	2.35 (2.06 to 2.65)	4.12 (3.64 to 4.65)	5.79 (5.13 to 6.57)	2.54 (2.22 to 2.87)	9.9 (5.7 to 14.3)	11.8 (5.7 to 18.2)	8.1 (2.9 to 13.8)
	Prevalence	75.5 (66.74 to 85.06)	107.41 (94.72 to 121.31)	45.29 (39.88 to 51.31)	84.02 (74.49 to 94.74)	119.88 (105.9 to 135.54)	48.98 (43.02 to 55.46)	11.3 (6.6 to 16.2)	11.6 (5.2 to 18.7)	8.2 (2.8 to 13.5)
	Deaths	0.06 (0.03 to 0.17)	0.1 (0.04 to 0.31)	0.03 (0.02 to 0.04)	0.08 (0.05 to 0.1)	0.1 (0.05 to 0.14)	0.05 (0.04 to 0.08)	27.1 (-61.2 to 182.5)	4.1 (-77.2 to 225.9)	103.6 (27.5 to 290.1)
	DALYs	11.44 (7.93 to 15.69)	16.29 (11.03 to 22.38)	6.87 (4.68 to 9.52)	12.91 (9.13 to 17.45)	18.03 (12.54 to 24.59)	7.9 (5.48 to 10.57)	12.9 (-8.9 to 31.7)	10.7 (-18.3 to 36.8)	15.1 (-3 to 36.5)
	YLLs	1.2 (0.61 to 3.32)	1.91 (0.73 to 6.41)	0.54 (0.36 to 0.78)	1.57 (1.05 to 2.01)	2.02 (1.03 to 2.87)	1.11 (0.79 to 1.55)	31.2 (-58.7 to 178.7)	5.6 (-75.8 to 214.1)	106.1 (30.8 to 279.8)
	YLDs	10.25 (6.92 to 14.19)	14.38 (9.67 to 20.03)	6.33 (4.19 to 8.95)	11.34 (7.5 to 15.83)	16.01 (10.59 to 22.65)	6.79 (4.37 to 9.55)	10.7 (-3.5 to 26.9)	11.4 (-6.6 to 32.8)	7.3 (-9.6 to 27.9)
Fars	Incidence	3.82 (3.39 to 4.32)	5.3 (4.74 to 5.97)	2.39 (2.08 to 2.73)	4.23 (3.75 to 4.74)	5.93 (5.21 to 6.68)	2.6 (2.28 to 2.93)	10.8 (6.3 to 15.2)	11.9 (5.7 to 18.2)	8.8 (3.4 to 14.6)
	Prevalence	77.32 (68.45 to 87.36)	110.17 (97.83 to 123.99)	46.16 (40.48 to 52.72)	86.39 (76.46 to 97.16)	123.35 (108.86 to 139.41)	50.33 (44.33 to 56.84)	11.7 (6.9 to 16.5)	12 (5.7 to 18.5)	9 (3.6 to 15)
	Deaths	0.06 (0.03 to 0.17)	0.09 (0.03 to 0.29)	0.03 (0.02 to 0.04)	0.08 (0.05 to 0.1)	0.1 (0.04 to 0.14)	0.05 (0.04 to 0.07)	29.5 (-63.1 to 192.6)	13.4 (-75.8 to 263.3)	94.5 (20.3 to 256.3)
	DALYs	11.55 (7.93 to 15.77)	16.35 (11.1 to 23.21)	6.98 (4.65 to 9.6)	13.22 (9.37 to 17.96)	18.51 (12.99 to 25.45)	8.06 (5.62 to 11.01)	14.5 (-7.1 to 34.8)	13.2 (-14.4 to 41.4)	15.6 (-2.8 to 37.4)
	YLLs	1.16 (0.59 to 3.35)	1.79 (0.66 to 6.11)	0.55 (0.33 to 0.78)	1.62 (0.99 to 2.13)	2.13 (0.97 to 3.08)	1.12 (0.8 to 1.56)	39.5 (-60.1 to 203.4)	19.1 (-74.3 to 265.8)	105.2 (27.1 to 282.2)
	YLDs	10.39 (7.01 to 14.3)	14.57 (9.9 to 20.53)	6.43 (4.16 to 9.05)	11.6 (7.79 to 16.16)	16.38 (11.07 to 23)	6.94 (4.54 to 9.83)	11.6 (-3 to 27.7)	12.4 (-7.4 to 35.5)	8 (-9.8 to 28.9)

Province	Measure	Age-standardized rate (per 100,000)						% Change (1990 to 2019)		
		1990			2019					
		Both	Female	Male	Both	Female	Male	Both	Female	Male
Gilan	Incidence	3.93 (3.47 to 4.41)	5.41 (4.77 to 6.1)	2.44 (2.15 to 2.77)	4.31 (3.83 to 4.83)	6.02 (5.36 to 6.77)	2.64 (2.32 to 3)	9.7 (5.1 to 14.8)	11.2 (5.1 to 17.6)	8.1 (2.5 to 14)
	Prevalence	79.97 (70.88 to 89.7)	112.66 (99.67 to 126.93)	47.3 (41.61 to 53.91)	88.14 (78.5 to 99.22)	125.12 (110.94 to 141.02)	51.14 (45.11 to 58.06)	10.2 (5.4 to 15.7)	11.1 (4.8 to 17.8)	8.1 (2.4 to 14.1)
	Deaths	0.07 (0.03 to 0.19)	0.1 (0.04 to 0.31)	0.03 (0.02 to 0.04)	0.11 (0.07 to 0.14)	0.14 (0.06 to 0.19)	0.08 (0.06 to 0.11)	62.5 (-54.8 to 275)	42.8 (-72.2 to 354.8)	157 (61.9 to 353.7)
	DALYs	12.1 (8.26 to 16.74)	16.94 (11.41 to 24.08)	7.21 (4.96 to 9.81)	14.24 (10.3 to 18.72)	19.59 (13.89 to 25.99)	8.88 (6.47 to 11.79)	17.6 (-5.6 to 38.9)	15.6 (-14.1 to 42.8)	23.1 (4.2 to 47.5)
	YLLs	1.33 (0.7 to 3.74)	1.96 (0.77 to 6.56)	0.63 (0.41 to 0.89)	2.4 (1.43 to 3.05)	2.95 (1.25 to 4.11)	1.83 (1.34 to 2.44)	79.9 (-48.8 to 286.6)	50.2 (-70.5 to 351)	188.3 (79.9 to 396)
	YLDs	10.77 (7.22 to 14.82)	14.98 (9.95 to 21.13)	6.58 (4.41 to 9.16)	11.84 (7.99 to 16.32)	16.64 (11.14 to 22.94)	7.05 (4.67 to 10.03)	9.9 (-4.2 to 26.4)	11.1 (-7.5 to 34.5)	7.2 (-9.2 to 26.7)
Golestan	Incidence	3.74 (3.32 to 4.22)	5.14 (4.55 to 5.84)	2.34 (2.05 to 2.67)	4.09 (3.64 to 4.63)	5.7 (5.05 to 6.45)	2.51 (2.21 to 2.86)	9.4 (5.1 to 13.9)	11 (5.2 to 16.9)	7.6 (2.2 to 13.1)
	Prevalence	75.3 (66.61 to 84.89)	106.48 (93.85 to 119.39)	45.11 (39.54 to 51.55)	83.62 (74.11 to 94.54)	118.15 (105 to 132.84)	48.58 (42.77 to 55.08)	11.1 (6.4 to 16.2)	11 (4.9 to 17.6)	7.7 (2 to 13.4)
	Deaths	0.06 (0.03 to 0.19)	0.09 (0.03 to 0.34)	0.03 (0.02 to 0.04)	0.09 (0.06 to 0.11)	0.11 (0.06 to 0.16)	0.06 (0.04 to 0.08)	39 (-58.8 to 234.2)	19.6 (-73.5 to 284.3)	107.4 (23.8 to 273.6)
	DALYs	11.47 (7.86 to 15.92)	16.23 (10.91 to 23.37)	6.87 (4.55 to 9.57)	13.19 (9.29 to 17.41)	18.37 (12.83 to 24.4)	7.92 (5.61 to 10.65)	15 (-9.5 to 36.3)	13.2 (-19.3 to 42.6)	15.3 (-1.7 to 37.8)
	YLLs	1.27 (0.62 to 3.8)	2.01 (0.75 to 7.11)	0.56 (0.35 to 0.84)	1.94 (1.23 to 2.57)	2.63 (1.32 to 3.76)	1.22 (0.87 to 1.7)	53.2 (-54.3 to 268.4)	31 (-70.5 to 318.2)	118.6 (29.7 to 282.6)
	YLDs	10.2 (6.86 to 14.14)	14.22 (9.48 to 20.11)	6.31 (4.08 to 8.99)	11.25 (7.51 to 15.49)	15.74 (10.28 to 21.65)	6.7 (4.4 to 9.42)	10.3 (-4.7 to 28.7)	10.7 (-9.2 to 36.5)	6.1 (-10.6 to 27.3)

Province	Measure	Age-standardized rate (per 100,000)						% Change (1990 to 2019)		
		1990			2019					
		Both	Female	Male	Both	Female	Male	Both	Female	Male
Hamadan	Incidence	3.73 (3.31 to 4.24)	5.17 (4.57 to 5.88)	2.35 (2.07 to 2.69)	4.11 (3.65 to 4.63)	5.75 (5.09 to 6.51)	2.53 (2.22 to 2.86)	10 (5.7 to 14.5)	11.1 (5.2 to 17.5)	7.7 (2.5 to 12.9)
	Prevalence	75.54 (66.74 to 85.35)	107.22 (94.48 to 121.76)	45.31 (39.7 to 51.53)	83.63 (74.62 to 94.04)	118.98 (105.79 to 134.14)	48.81 (43 to 55.25)	10.7 (6.2 to 15.4)	11 (4.8 to 17.5)	7.7 (2.3 to 13.1)
	Deaths	0.06 (0.03 to 0.15)	0.1 (0.04 to 0.28)	0.02 (0.02 to 0.04)	0.08 (0.04 to 0.11)	0.13 (0.04 to 0.18)	0.04 (0.03 to 0.06)	36.6 (-59.1 to 193.1)	28.2 (-72.5 to 266.3)	62 (2.2 to 195.6)
	DALYs	11.47 (8.06 to 15.5)	16.31 (11.26 to 22.6)	6.88 (4.75 to 9.46)	13.11 (9.36 to 17.55)	18.62 (13.04 to 25.3)	7.66 (5.27 to 10.48)	14.3 (-7.9 to 35)	14.2 (-17 to 43.4)	11.4 (-6.5 to 31.2)
	YLLs	1.27 (0.73 to 3.05)	2.04 (0.93 to 5.76)	0.55 (0.37 to 0.83)	1.84 (0.89 to 2.45)	2.78 (0.95 to 3.94)	0.89 (0.64 to 1.23)	45.4 (-56 to 200)	36.3 (-71.1 to 266.3)	60.9 (1.5 to 183)
	YLDs	10.21 (6.96 to 14.09)	14.27 (9.71 to 19.83)	6.32 (4.22 to 8.88)	11.27 (7.57 to 15.66)	15.84 (10.66 to 22.33)	6.77 (4.45 to 9.57)	10.4 (-4.8 to 27.1)	11 (-8.5 to 35)	7 (-9.9 to 28)
Hormozgan	Incidence	3.63 (3.22 to 4.11)	5.04 (4.49 to 5.74)	2.28 (2 to 2.6)	4.1 (3.65 to 4.62)	5.79 (5.12 to 6.54)	2.51 (2.23 to 2.84)	13.2 (8.3 to 18.2)	14.8 (8.2 to 21.8)	10.1 (5 to 16.2)
	Prevalence	72.25 (64.56 to 81.62)	104 (92.49 to 117.63)	43.8 (38.56 to 49.86)	83.35 (73.67 to 93.8)	119.54 (105.17 to 134.93)	48.35 (42.74 to 54.55)	15.4 (10.3 to 20.4)	14.9 (8 to 21.8)	10.4 (5.2 to 16.4)
	Deaths	0.07 (0.04 to 0.13)	0.1 (0.04 to 0.23)	0.03 (0.02 to 0.05)	0.12 (0.05 to 0.16)	0.16 (0.03 to 0.25)	0.08 (0.06 to 0.12)	80.9 (-44.1 to 307.8)	58.2 (-77.6 to 418.8)	147.4 (45.3 to 343.4)
	DALYs	11.12 (7.79 to 14.89)	15.91 (10.89 to 21.93)	6.82 (4.74 to 9.35)	14 (9.99 to 18.6)	19.41 (13.17 to 26.43)	8.73 (6.36 to 11.44)	25.9 (2.3 to 50.3)	22 (-9.8 to 55.7)	27.9 (7.3 to 57.1)
	YLLs	1.36 (0.74 to 2.56)	2.1 (0.9 to 4.66)	0.71 (0.44 to 1.05)	2.77 (1.17 to 3.7)	3.5 (0.58 to 5.23)	2.02 (1.43 to 2.92)	103 (-36.4 to 336.5)	66.7 (-77.1 to 415.6)	185 (68.9 to 421.6)
	YLDs	9.76 (6.46 to 13.43)	13.81 (9.07 to 19.29)	6.11 (4.05 to 8.64)	11.23 (7.58 to 15.75)	15.91 (10.8 to 22.61)	6.7 (4.52 to 9.38)	15.1 (-0.4 to 31.6)	15.2 (-4.1 to 39.8)	9.6 (-7.5 to 30.6)

Province	Measure	Age-standardized rate (per 100,000)						% Change (1990 to 2019)		
		1990			2019					
		Both	Female	Male	Both	Female	Male	Both	Female	Male
Ilam	Incidence	3.66 (3.25 to 4.12)	5.09 (4.51 to 5.75)	2.29 (2 to 2.6)	4.17 (3.69 to 4.67)	5.85 (5.2 to 6.6)	2.55 (2.24 to 2.9)	13.9 (9.4 to 18.6)	14.8 (8.7 to 21.5)	11.5 (6.4 to 17.3)
	Prevalence	72.49 (63.97 to 82.07)	105.32 (92.85 to 119.33)	44.05 (38.62 to 50.05)	85.08 (75.43 to 95.54)	121.31 (107.07 to 136.65)	49.34 (43.36 to 55.84)	17.4 (12.3 to 22.5)	15.2 (8 to 22.2)	12 (7 to 17.9)
	Deaths	0.05 (0.03 to 0.16)	0.1 (0.03 to 0.34)	0.02 (0.01 to 0.03)	0.07 (0.04 to 0.1)	0.11 (0.05 to 0.15)	0.04 (0.03 to 0.06)	34.7 (-62 to 230.1)	11.1 (-77.4 to 325.7)	91.2 (10.8 to 298.1)
	DALYs	10.92 (7.44 to 14.71)	15.99 (10.71 to 22.69)	6.6 (4.45 to 8.95)	12.98 (9.35 to 17.34)	18.34 (13.1 to 24.62)	7.69 (5.35 to 10.45)	18.9 (-4.7 to 39.3)	14.7 (-16.5 to 44.9)	16.5 (-0.9 to 37.2)
	YLLs	1.1 (0.52 to 3.27)	1.95 (0.63 to 7.03)	0.45 (0.28 to 0.68)	1.52 (0.92 to 2.02)	2.19 (1.01 to 3.09)	0.86 (0.63 to 1.19)	38.7 (-59.1 to 235)	12.1 (-76 to 311.8)	89.6 (13.3 to 274.8)
	YLDs	9.82 (6.54 to 13.44)	14.04 (9.24 to 19.34)	6.15 (4.03 to 8.43)	11.46 (7.8 to 15.98)	16.15 (11.02 to 22.6)	6.83 (4.5 to 9.62)	16.7 (0.9 to 34.9)	15 (-4.2 to 40.5)	11.1 (-8 to 32)
Isfahan	Incidence	3.85 (3.41 to 4.35)	5.41 (4.78 to 6.12)	2.42 (2.12 to 2.75)	4.25 (3.78 to 4.78)	5.97 (5.28 to 6.72)	2.59 (2.28 to 2.94)	10.4 (5.9 to 14.5)	10.3 (4.2 to 16.3)	7.2 (2.1 to 13)
	Prevalence	78.61 (69.59 to 88.84)	112.67 (99.69 to 127.47)	46.88 (41.12 to 53.18)	86.52 (76.49 to 97.12)	123.86 (109.83 to 139.47)	50.24 (44.2 to 56.9)	10.1 (5.5 to 14.5)	9.9 (3.5 to 16.1)	7.2 (2.1 to 12.9)
	Deaths	0.06 (0.03 to 0.13)	0.08 (0.03 to 0.22)	0.03 (0.02 to 0.04)	0.08 (0.04 to 0.11)	0.12 (0.03 to 0.18)	0.05 (0.03 to 0.08)	46.1 (-60.8 to 234.9)	40.7 (-78.1 to 342.6)	93.4 (10.9 to 280.2)
	DALYs	11.68 (7.95 to 15.91)	16.58 (11.33 to 22.81)	7.06 (4.74 to 9.65)	13.45 (9.55 to 18.01)	19.03 (13.29 to 26.08)	8.02 (5.54 to 10.78)	15.1 (-5.6 to 34.8)	14.8 (-12.5 to 43)	13.7 (-4.1 to 36.5)
	YLLs	1.12 (0.59 to 2.5)	1.67 (0.71 to 4.34)	0.54 (0.35 to 0.8)	1.77 (0.81 to 2.45)	2.5 (0.68 to 3.75)	1.07 (0.75 to 1.54)	58.2 (-56.5 to 247.4)	49.4 (-76.1 to 354)	98 (16.6 to 280.7)
	YLDs	10.56 (7.04 to 14.63)	14.91 (9.91 to 20.71)	6.52 (4.26 to 9.11)	11.67 (7.84 to 16.06)	16.53 (10.94 to 23.03)	6.95 (4.56 to 9.79)	10.5 (-4.3 to 27.1)	10.9 (-8.5 to 35.1)	6.7 (-10.3 to 27.3)

Province	Measure	Age-standardized rate (per 100,000)						% Change (1990 to 2019)		
		1990			2019					
		Both	Female	Male	Both	Female	Male	Both	Female	Male
Kerman	Incidence	3.75 (3.32 to 4.21)	5.16 (4.58 to 5.85)	2.36 (2.07 to 2.68)	3.99 (3.55 to 4.49)	5.6 (4.96 to 6.31)	2.49 (2.19 to 2.83)	6.5 (1.9 to 10.8)	8.5 (2.5 to 14.8)	5.4 (-0.1 to 10.5)
	Prevalence	75.57 (66.88 to 85.11)	107.09 (94.35 to 120.98)	45.62 (40.01 to 51.79)	81.05 (72.01 to 91.77)	115.87 (102.55 to 130.52)	47.93 (42.39 to 54.56)	7.3 (2.4 to 12.1)	8.2 (1.8 to 15.2)	5.1 (-0.5 to 10.6)
	Deaths	0.06 (0.03 to 0.14)	0.09 (0.04 to 0.24)	0.03 (0.02 to 0.04)	0.07 (0.04 to 0.1)	0.1 (0.03 to 0.14)	0.05 (0.03 to 0.07)	20 (-62.1 to 150.4)	7.9 (-76.9 to 209.2)	63.3 (-2.8 to 203.3)
	DALYs	11.39 (7.92 to 15.45)	16.06 (10.99 to 22.3)	6.94 (4.8 to 9.47)	12.39 (8.8 to 16.58)	17.41 (12.34 to 23.93)	7.61 (5.37 to 10.43)	8.8 (-10 to 25.9)	8.4 (-17.4 to 33.2)	9.7 (-6.3 to 28.3)
	YLLs	1.21 (0.66 to 2.78)	1.85 (0.77 to 5.02)	0.59 (0.39 to 0.87)	1.52 (0.81 to 2.09)	2.07 (0.74 to 3.13)	0.97 (0.67 to 1.42)	25.7 (-59.4 to 152.2)	12 (-75.1 to 208.3)	63.6 (-0.5 to 184.3)
	YLDs	10.18 (6.8 to 14.21)	14.21 (9.21 to 20.26)	6.35 (4.24 to 8.94)	10.88 (7.3 to 15.03)	15.33 (10.31 to 21.55)	6.64 (4.43 to 9.38)	6.8 (-7.1 to 21.9)	7.9 (-10.2 to 30.5)	4.6 (-11.4 to 22.4)
Kermanshah	Incidence	3.69 (3.27 to 4.19)	5.12 (4.53 to 5.8)	2.31 (2.02 to 2.64)	4.11 (3.64 to 4.63)	5.76 (5.1 to 6.48)	2.52 (2.21 to 2.85)	11.4 (6.7 to 16.6)	12.5 (6.8 to 19.2)	8.9 (3.2 to 14.2)
	Prevalence	73.9 (65.32 to 83.21)	105.95 (94.2 to 119.49)	44.6 (38.8 to 50.65)	83.98 (74.77 to 94.9)	119.16 (105.84 to 134.82)	48.58 (42.68 to 55.1)	13.6 (8.8 to 18.8)	12.5 (6.3 to 19.3)	8.9 (3.3 to 14.5)
	Deaths	0.06 (0.03 to 0.14)	0.1 (0.04 to 0.28)	0.03 (0.02 to 0.04)	0.09 (0.05 to 0.12)	0.12 (0.04 to 0.18)	0.06 (0.04 to 0.09)	51.8 (-54.8 to 225)	25.6 (-77.5 to 295.8)	112.7 (30.6 to 291.5)
	DALYs	11.26 (7.78 to 15.1)	16.14 (11.23 to 22.43)	6.84 (4.66 to 9.33)	13.32 (9.41 to 17.62)	18.48 (12.91 to 24.8)	8.12 (5.78 to 10.77)	18.4 (-5.1 to 38.2)	14.5 (-17.4 to 42.7)	18.7 (1.2 to 41.4)
	YLLs	1.23 (0.68 to 3.01)	1.99 (0.81 to 6.02)	0.6 (0.4 to 0.85)	2.01 (1.07 to 2.66)	2.6 (0.83 to 3.76)	1.41 (1.01 to 1.96)	63.6 (-51.6 to 243.7)	30.2 (-76.6 to 293.4)	136.4 (47.4 to 333.9)
	YLDs	10.03 (6.68 to 13.84)	14.15 (9.41 to 19.69)	6.24 (4.07 to 8.68)	11.31 (7.63 to 15.38)	15.89 (10.64 to 21.83)	6.71 (4.42 to 9.38)	12.8 (-1.3 to 28.8)	12.3 (-6.4 to 34.8)	7.5 (-9.3 to 26.7)

Province	Measure	Age-standardized rate (per 100,000)						% Change (1990 to 2019)		
		1990			2019					
		Both	Female	Male	Both	Female	Male	Both	Female	Male
Khorasan-e-Razavi	Incidence	3.68 (3.26 to 4.15)	5.06 (4.47 to 5.72)	2.31 (2.02 to 2.61)	4.02 (3.56 to 4.51)	5.6 (4.94 to 6.31)	2.48 (2.18 to 2.82)	9.1 (4.5 to 14)	10.6 (4.2 to 16.8)	7.2 (2.3 to 13.2)
	Prevalence	74 (65.5 to 83.23)	104.78 (92.44 to 118.69)	44.51 (38.93 to 50.34)	81.66 (72.14 to 92.01)	115.71 (102.02 to 130.79)	47.71 (42.01 to 53.94)	10.4 (5.2 to 15.6)	10.4 (3.5 to 16.9)	7.2 (2.2 to 13.2)
	Deaths	0.06 (0.03 to 0.16)	0.09 (0.04 to 0.3)	0.03 (0.02 to 0.04)	0.08 (0.05 to 0.1)	0.1 (0.04 to 0.15)	0.05 (0.04 to 0.07)	28.3 (-59.5 to 188.2)	9.3 (-75.8 to 240.5)	86 (17.1 to 220)
	DALYs	11.23 (7.86 to 15.39)	15.89 (10.91 to 22.21)	6.79 (4.69 to 9.26)	12.66 (9.08 to 17.03)	17.62 (12.51 to 24.02)	7.71 (5.43 to 10.25)	12.7 (-9 to 32)	10.9 (-16.9 to 37.4)	13.6 (-2.9 to 33.7)
	YLLs	1.25 (0.65 to 3.37)	1.97 (0.77 to 6.38)	0.59 (0.39 to 0.87)	1.67 (1.02 to 2.19)	2.22 (0.97 to 3.18)	1.11 (0.82 to 1.5)	34 (-56.4 to 187.2)	12.9 (-74.7 to 240.4)	89.4 (17.6 to 220.3)
	YLDs	9.98 (6.79 to 13.76)	13.92 (9.48 to 19.24)	6.2 (4.16 to 8.77)	10.99 (7.48 to 15.28)	15.4 (10.58 to 21.83)	6.6 (4.33 to 9.17)	10.1 (-4.5 to 26.1)	10.6 (-8.4 to 32.6)	6.5 (-10.4 to 25.1)
Khuzestan	Incidence	3.73 (3.32 to 4.19)	5.16 (4.59 to 5.78)	2.36 (2.06 to 2.69)	4.11 (3.64 to 4.61)	5.71 (5.07 to 6.43)	2.55 (2.24 to 2.89)	10 (5.6 to 14.5)	10.7 (4.4 to 17)	8.1 (2.8 to 13.4)
	Prevalence	75.42 (66.9 to 84.53)	107.03 (94.94 to 119.82)	45.54 (40.09 to 51.87)	83.4 (73.82 to 93.32)	118.28 (104.49 to 133.31)	49.21 (43.45 to 55.7)	10.6 (5.9 to 15.3)	10.5 (4.4 to 17)	8.1 (2.6 to 13.5)
	Deaths	0.06 (0.03 to 0.16)	0.1 (0.04 to 0.28)	0.03 (0.02 to 0.04)	0.08 (0.05 to 0.1)	0.11 (0.05 to 0.16)	0.04 (0.03 to 0.06)	19 (-58.7 to 159.7)	12.2 (-70.2 to 233.4)	57.4 (-1.7 to 192.2)
	DALYs	11.47 (7.85 to 15.4)	16.25 (10.87 to 22.27)	6.91 (4.74 to 9.42)	12.85 (9.2 to 17.06)	18.08 (12.79 to 24.19)	7.71 (5.29 to 10.48)	12.1 (-8.7 to 31.4)	11.3 (-17 to 38.2)	11.6 (-4.9 to 30)
	YLLs	1.28 (0.68 to 3.21)	2 (0.83 to 5.9)	0.57 (0.36 to 0.84)	1.65 (0.98 to 2.2)	2.39 (1.11 to 3.38)	0.91 (0.65 to 1.27)	28.5 (-56.2 to 167.7)	19.4 (-68 to 241.8)	60.1 (-1 to 184.3)
	YLDs	10.18 (6.77 to 14.13)	14.25 (9.39 to 19.73)	6.34 (4.16 to 8.87)	11.2 (7.43 to 15.49)	15.69 (10.48 to 21.85)	6.81 (4.42 to 9.53)	10 (-3.5 to 25.9)	10.1 (-7.3 to 33.5)	7.3 (-10.8 to 25.2)

Province	Measure	Age-standardized rate (per 100,000)						% Change (1990 to 2019)		
		1990			2019					
		Both	Female	Male	Both	Female	Male	Both	Female	Male
Kohgiluyeh and Boyer-Ahmad	Incidence	3.62 (3.18 to 4.08)	5.02 (4.41 to 5.69)	2.29 (2 to 2.59)	4.11 (3.65 to 4.64)	5.76 (5.11 to 6.48)	2.53 (2.23 to 2.88)	13.5 (9.3 to 18.1)	14.7 (9.1 to 20.9)	10.5 (5 to 16)
	Prevalence	72.2 (63.86 to 81.53)	103.78 (91.33 to 117.08)	44.03 (38.47 to 50.01)	83.41 (74.16 to 93.99)	119.38 (105.61 to 134.75)	48.88 (43.22 to 55.16)	15.5 (11 to 20.5)	15 (8.8 to 21.8)	11 (5.5 to 16.9)
	Deaths	0.06 (0.03 to 0.14)	0.09 (0.03 to 0.26)	0.03 (0.02 to 0.04)	0.07 (0.04 to 0.09)	0.1 (0.04 to 0.15)	0.04 (0.03 to 0.06)	13.2 (-60.4 to 168.5)	7.8 (-75.1 to 279.1)	62.1 (-8.3 to 198.1)
	DALYs	10.92 (7.6 to 14.85)	15.64 (10.65 to 21.6)	6.67 (4.48 to 9.15)	12.64 (8.85 to 17)	17.87 (12.42 to 24.35)	7.65 (5.27 to 10.4)	15.7 (-3.3 to 34.7)	14.3 (-11.5 to 39.9)	14.6 (-3.5 to 35.9)
	YLLs	1.16 (0.59 to 2.9)	1.82 (0.65 to 5.47)	0.54 (0.33 to 0.82)	1.38 (0.82 to 1.89)	1.94 (0.86 to 2.85)	0.89 (0.6 to 1.29)	18.6 (-58 to 161.3)	6.7 (-75.3 to 255.7)	63.9 (-5.8 to 200.4)
	YLDs	9.76 (6.43 to 13.4)	13.82 (9.22 to 19.25)	6.13 (3.91 to 8.68)	11.26 (7.5 to 15.56)	15.93 (10.69 to 22.27)	6.76 (4.35 to 9.39)	15.3 (0.3 to 32)	15.3 (-3.9 to 37.3)	10.2 (-7.7 to 31.1)
Kurdistan	Incidence	3.69 (3.28 to 4.15)	5.13 (4.57 to 5.77)	2.27 (2 to 2.58)	4.09 (3.64 to 4.6)	5.78 (5.12 to 6.51)	2.46 (2.17 to 2.78)	10.7 (6.2 to 15.5)	12.7 (6.6 to 18.9)	8.3 (3.1 to 13.9)
	Prevalence	73.6 (65.45 to 82.69)	106.01 (94.05 to 119.83)	43.75 (38.65 to 49.62)	83.04 (74.02 to 93.28)	119.34 (105.72 to 135.04)	47.44 (41.9 to 53.68)	12.8 (7.9 to 17.9)	12.6 (6.4 to 19.3)	8.4 (3.2 to 14.1)
	Deaths	0.06 (0.04 to 0.12)	0.09 (0.04 to 0.22)	0.03 (0.02 to 0.04)	0.1 (0.04 to 0.14)	0.15 (0.02 to 0.24)	0.05 (0.03 to 0.07)	69.6 (-59.2 to 256)	62.9 (-80.8 to 374.2)	60.7 (-3.1 to 184)
	DALYs	11.18 (7.72 to 15.09)	16.09 (10.97 to 22.35)	6.72 (4.67 to 9.22)	13.35 (9.59 to 17.61)	19.17 (13.31 to 25.77)	7.62 (5.34 to 10.3)	19.4 (-5.7 to 41.9)	19.2 (-15 to 51.5)	13.4 (-2.6 to 34)
	YLLs	1.21 (0.75 to 2.38)	1.95 (0.92 to 4.61)	0.6 (0.4 to 0.87)	2.15 (0.77 to 2.98)	3.27 (0.53 to 4.99)	1.03 (0.74 to 1.45)	77.9 (-57.8 to 268.5)	67.5 (-80.2 to 371.2)	70.3 (2.9 to 207)
	YLDs	9.97 (6.6 to 13.68)	14.14 (9.26 to 19.6)	6.12 (4.05 to 8.62)	11.2 (7.6 to 15.24)	15.9 (10.88 to 21.67)	6.59 (4.32 to 9.31)	12.3 (-3.2 to 29.8)	12.5 (-8.2 to 37.8)	7.7 (-8.3 to 27.2)

Province	Measure	Age-standardized rate (per 100,000)						% Change (1990 to 2019)		
		1990			2019					
		Both	Female	Male	Both	Female	Male	Both	Female	Male
Lorestan	Incidence	3.7 (3.28 to 4.18)	5.08 (4.51 to 5.74)	2.34 (2.06 to 2.66)	4.11 (3.63 to 4.64)	5.72 (5.05 to 6.5)	2.56 (2.25 to 2.91)	11.2 (6.3 to 16.1)	12.6 (6 to 19.4)	9.4 (4 to 15.4)
	Prevalence	73.79 (65.73 to 83.05)	105.13 (93.07 to 118.59)	45.04 (39.78 to 51.1)	84.02 (74.46 to 94.45)	118.65 (104.55 to 134.43)	49.34 (43.15 to 56.05)	13.9 (8.8 to 19.2)	12.9 (5.9 to 20)	9.5 (4.2 to 16)
	Deaths	0.06 (0.04 to 0.14)	0.1 (0.04 to 0.27)	0.03 (0.02 to 0.04)	0.05 (0.04 to 0.08)	0.07 (0.04 to 0.12)	0.03 (0.02 to 0.05)	-19.9 (-59.4 to 56.7)	-31.8 (-69.2 to 80)	10.9 (-31.3 to 106.4)
	DALYs	11.3 (7.81 to 15.13)	16.16 (11.08 to 21.85)	6.89 (4.77 to 9.35)	12.41 (8.6 to 16.84)	17.29 (12.12 to 23.75)	7.5 (5.11 to 10.24)	9.8 (-6.9 to 26)	7 (-14.9 to 30.3)	8.9 (-8.3 to 28.6)
	YLLs	1.31 (0.75 to 2.92)	2.14 (0.93 to 5.65)	0.59 (0.38 to 0.9)	1.08 (0.76 to 1.64)	1.5 (0.92 to 2.59)	0.64 (0.44 to 0.92)	-17.2 (-56.6 to 51.8)	-30 (-67.1 to 75)	7.9 (-36.3 to 97.8)
	YLDs	9.99 (6.63 to 13.87)	14.01 (9.26 to 19.52)	6.29 (4.19 to 8.72)	11.33 (7.59 to 15.68)	15.79 (10.57 to 22.04)	6.86 (4.46 to 9.64)	13.3 (-1.7 to 29.5)	12.7 (-5.7 to 34.6)	9 (-9.5 to 30)
Markazi	Incidence	3.82 (3.39 to 4.32)	5.25 (4.65 to 5.93)	2.37 (2.07 to 2.7)	4.15 (3.68 to 4.68)	5.84 (5.18 to 6.62)	2.54 (2.24 to 2.89)	8.8 (4.2 to 13.9)	11.3 (5 to 18.6)	7.2 (1.4 to 12.9)
	Prevalence	77.32 (68.57 to 87.01)	108.77 (95.95 to 122.41)	45.7 (40.4 to 52.05)	84.54 (75.15 to 95.21)	120.84 (107.01 to 136.68)	48.96 (43.11 to 55.8)	9.3 (4.8 to 14.5)	11.1 (4.7 to 18.4)	7.1 (1.4 to 13.1)
	Deaths	0.06 (0.03 to 0.15)	0.1 (0.04 to 0.27)	0.02 (0.01 to 0.03)	0.09 (0.04 to 0.12)	0.13 (0.04 to 0.19)	0.05 (0.04 to 0.07)	48.9 (-59.8 to 226.7)	32.6 (-76.1 to 275.4)	116.4 (36.2 to 272.3)
	DALYs	11.66 (8 to 15.77)	16.46 (11.27 to 22.94)	6.85 (4.58 to 9.45)	13.38 (9.52 to 17.77)	18.87 (13.18 to 25.21)	7.95 (5.65 to 10.68)	14.7 (-8.2 to 35.7)	14.6 (-15.8 to 44.1)	16.1 (-1.7 to 37)
	YLLs	1.25 (0.69 to 3.1)	2.02 (0.9 to 5.79)	0.51 (0.32 to 0.74)	2.02 (0.96 to 2.67)	2.82 (0.85 to 4.02)	1.19 (0.85 to 1.64)	61 (-56.5 to 243.2)	39.9 (-75.6 to 282.7)	132.2 (47.2 to 307.3)
	YLDs	10.4 (7.02 to 14.21)	14.45 (9.8 to 19.85)	6.34 (4.1 to 8.91)	11.36 (7.59 to 15.61)	16.05 (10.71 to 22.08)	6.76 (4.44 to 9.68)	9.2 (-5.3 to 23.7)	11.1 (-6.8 to 32.4)	6.7 (-10.7 to 26.8)

Province	Measure	Age-standardized rate (per 100,000)						% Change (1990 to 2019)		
		1990			2019					
		Both	Female	Male	Both	Female	Male	Both	Female	Male
Mazandaran	Incidence	4 (3.54 to 4.53)	5.51 (4.86 to 6.26)	2.49 (2.18 to 2.82)	4.4 (3.91 to 4.96)	6.15 (5.44 to 6.95)	2.7 (2.38 to 3.06)	10.1 (5.3 to 15.2)	11.6 (5.2 to 18.1)	8.7 (2.5 to 14.6)
	Prevalence	81.35 (72.14 to 91.37)	114.81 (101.35 to 129.75)	48.24 (42.44 to 54.59)	90.3 (79.86 to 101.68)	128.17 (112.74 to 144.3)	52.57 (46.32 to 59.71)	11 (6 to 16.5)	11.6 (5 to 19.1)	9 (3.5 to 14.4)
	Deaths	0.07 (0.04 to 0.14)	0.1 (0.04 to 0.24)	0.04 (0.02 to 0.05)	0.1 (0.06 to 0.13)	0.12 (0.05 to 0.18)	0.07 (0.05 to 0.1)	41.8 (-49 to 180.9)	24.3 (-70.8 to 258.4)	101.4 (22.2 to 254.9)
	DALYs	12.21 (8.57 to 16.44)	17.05 (11.75 to 23.48)	7.4 (5.16 to 10.02)	14.2 (10.07 to 18.92)	19.63 (13.64 to 26.8)	8.78 (6.31 to 12.09)	16.3 (-4.7 to 38.4)	15.1 (-11.3 to 43.2)	18.6 (0 to 42.6)
	YLLs	1.39 (0.82 to 2.91)	2.04 (0.92 to 5.08)	0.72 (0.49 to 1.05)	2.18 (1.26 to 2.87)	2.75 (1.09 to 4.06)	1.6 (1.16 to 2.2)	57.4 (-43.6 to 204.4)	35.1 (-68.1 to 265.1)	121.2 (33.6 to 275.7)
	YLDs	10.82 (7.32 to 14.76)	15.01 (10.05 to 20.8)	6.68 (4.45 to 9.3)	12.02 (8.06 to 16.77)	16.88 (11.35 to 23.81)	7.18 (4.78 to 10.34)	11.1 (-3.8 to 28.8)	12.4 (-7 to 37.1)	7.6 (-9.3 to 30)
North Khorasan	Incidence	3.64 (3.24 to 4.1)	5.01 (4.45 to 5.65)	2.26 (1.99 to 2.56)	4.08 (3.62 to 4.61)	5.73 (5.08 to 6.47)	2.47 (2.18 to 2.81)	12.1 (7.7 to 16.9)	14.4 (8.5 to 21)	9.3 (3.8 to 14.5)
	Prevalence	72.85 (64.8 to 81.83)	103.31 (91.8 to 115.99)	43.39 (38.05 to 49.03)	83.14 (73.71 to 93.85)	118.26 (104.82 to 133.94)	47.63 (41.86 to 54.15)	14.1 (9.4 to 19.3)	14.5 (8.2 to 21.3)	9.8 (4.4 to 15.3)
	Deaths	0.07 (0.04 to 0.17)	0.11 (0.04 to 0.32)	0.03 (0.02 to 0.05)	0.1 (0.06 to 0.13)	0.15 (0.07 to 0.22)	0.05 (0.03 to 0.07)	40.3 (-52.5 to 211.3)	36.3 (-66.2 to 319.2)	51.5 (-13.8 to 180.2)
	DALYs	11.33 (7.88 to 15.49)	16.15 (11.12 to 22.65)	6.72 (4.6 to 9.18)	13.42 (9.67 to 17.81)	19.18 (13.79 to 25.88)	7.61 (5.36 to 10.12)	18.5 (-6.5 to 41.2)	18.8 (-13.2 to 50.1)	13.2 (-3.3 to 33.3)
	YLLs	1.48 (0.81 to 3.68)	2.4 (0.99 to 6.83)	0.66 (0.39 to 1)	2.26 (1.22 to 2.98)	3.5 (1.51 to 4.79)	1.01 (0.72 to 1.47)	52.2 (-50.2 to 217.3)	45.8 (-65.3 to 327.9)	52.4 (-11.1 to 184.2)
	YLDs	9.84 (6.56 to 13.81)	13.75 (9.07 to 19.38)	6.06 (4.01 to 8.46)	11.16 (7.52 to 15.5)	15.68 (10.45 to 22.1)	6.6 (4.45 to 9.12)	13.4 (-1.4 to 30.1)	14.1 (-5.2 to 37.8)	9 (-7.4 to 28.8)

Province	Measure	Age-standardized rate (per 100,000)						% Change (1990 to 2019)		
		1990			2019					
		Both	Female	Male	Both	Female	Male	Both	Female	Male
Qazvin	Incidence	3.68 (3.28 to 4.17)	5.09 (4.52 to 5.78)	2.31 (2.04 to 2.64)	4.11 (3.64 to 4.67)	5.77 (5.12 to 6.53)	2.54 (2.23 to 2.9)	11.5 (7 to 16.2)	13.3 (7.1 to 20)	9.6 (4.2 to 15.2)
	Prevalence	74.38 (65.67 to 83.82)	105.51 (92.97 to 119.35)	44.57 (39.03 to 50.66)	83.74 (74.58 to 94.3)	119.68 (106.67 to 134.92)	48.95 (42.99 to 55.45)	12.6 (7.9 to 17.9)	13.4 (7.3 to 20.6)	9.8 (4.5 to 15.4)
	Deaths	0.06 (0.03 to 0.15)	0.1 (0.04 to 0.26)	0.02 (0.01 to 0.03)	0.07 (0.04 to 0.09)	0.09 (0.04 to 0.13)	0.04 (0.03 to 0.06)	14.4 (-61.6 to 165.9)	-4.5 (-76.9 to 205)	99.3 (22.8 to 276.2)
	DALYs	11.23 (7.68 to 15.08)	15.96 (10.88 to 22.2)	6.69 (4.52 to 9.35)	12.75 (8.94 to 17.28)	17.92 (12.28 to 24.72)	7.71 (5.36 to 10.46)	13.5 (-6.2 to 31.9)	12.3 (-13.6 to 38.4)	15.2 (-3.2 to 36.3)
	YLLs	1.18 (0.6 to 2.82)	1.92 (0.78 to 5.3)	0.47 (0.28 to 0.68)	1.43 (0.83 to 1.87)	1.93 (0.79 to 2.74)	0.92 (0.69 to 1.25)	21 (-58.1 to 163.5)	0.1 (-74.9 to 195.5)	97.2 (24.5 to 259.4)
	YLDs	10.05 (6.7 to 13.76)	14.03 (9.37 to 19.29)	6.23 (4.06 to 8.89)	11.32 (7.57 to 15.84)	16 (10.65 to 22.63)	6.79 (4.51 to 9.54)	12.6 (-1.6 to 28.8)	14 (-5 to 37.6)	9.1 (-8.8 to 30.4)
Qom	Incidence	3.68 (3.28 to 4.15)	5.17 (4.59 to 5.83)	2.3 (2.02 to 2.61)	4.15 (3.67 to 4.66)	5.83 (5.18 to 6.55)	2.52 (2.22 to 2.88)	12.7 (8.2 to 17.2)	12.9 (6.6 to 19.5)	9.6 (4.3 to 15.7)
	Prevalence	74.39 (66.02 to 83.46)	106.92 (94.85 to 119.85)	44.42 (39.03 to 50.42)	83.89 (74.53 to 94.13)	120.79 (107.33 to 135.36)	48.78 (42.85 to 55.69)	12.8 (8.2 to 17.7)	13 (6.8 to 20)	9.8 (4.3 to 16.2)
	Deaths	0.07 (0.04 to 0.14)	0.11 (0.05 to 0.26)	0.03 (0.02 to 0.04)	0.08 (0.05 to 0.1)	0.11 (0.05 to 0.16)	0.05 (0.03 to 0.06)	3.9 (-56.2 to 110.6)	-1.4 (-69.1 to 154.8)	43.8 (-7.6 to 140.6)
	DALYs	11.46 (8.02 to 15.42)	16.43 (11.54 to 22.72)	6.84 (4.78 to 9.31)	12.89 (9.13 to 17.17)	18.28 (12.78 to 24.58)	7.76 (5.41 to 10.43)	12.4 (-6.3 to 31.2)	11.3 (-12.4 to 36.2)	13.4 (-2.8 to 33.4)
	YLLs	1.46 (0.81 to 3)	2.26 (1.01 to 5.37)	0.66 (0.38 to 0.96)	1.58 (0.96 to 2.02)	2.22 (0.99 to 3.11)	0.99 (0.69 to 1.31)	8.6 (-53.6 to 110.7)	-1.8 (-68.3 to 143.2)	49.6 (-4.1 to 160.7)
	YLDs	10.01 (6.83 to 13.83)	14.17 (9.44 to 19.66)	6.18 (4.03 to 8.72)	11.3 (7.6 to 15.59)	16.06 (10.76 to 22.27)	6.76 (4.47 to 9.52)	12.9 (-0.5 to 28.2)	13.4 (-4.7 to 35.6)	9.5 (-7.7 to 30.1)

Province	Measure	Age-standardized rate (per 100,000)						% Change (1990 to 2019)		
		1990			2019					
		Both	Female	Male	Both	Female	Male	Both	Female	Male
Semnan	Incidence	3.75 (3.31 to 4.23)	5.19 (4.59 to 5.88)	2.37 (2.07 to 2.68)	4.2 (3.73 to 4.72)	5.88 (5.2 to 6.64)	2.6 (2.3 to 2.95)	12 (7.7 to 16.4)	13.1 (7.5 to 19.6)	9.7 (4.7 to 15.3)
	Prevalence	76.32 (67.58 to 86.46)	107.67 (95.38 to 122.02)	45.63 (40.21 to 51.7)	85.61 (75.94 to 96.55)	122.07 (107.93 to 138.18)	50.04 (44.26 to 56.92)	12.2 (7.8 to 16.9)	13.4 (7.1 to 20.2)	9.7 (4 to 15.2)
	Deaths	0.07 (0.03 to 0.21)	0.1 (0.04 to 0.38)	0.02 (0.02 to 0.04)	0.09 (0.06 to 0.12)	0.12 (0.06 to 0.16)	0.06 (0.04 to 0.08)	31.6 (-61.3 to 227.3)	11.9 (-73.5 to 266.8)	135.5 (46 to 303.8)
	DALYs	11.62 (8.05 to 16.04)	16.44 (11.18 to 23.35)	6.86 (4.7 to 9.44)	13.34 (9.63 to 17.78)	18.63 (13.13 to 24.92)	8.15 (5.85 to 11.1)	14.8 (-11 to 35.1)	13.4 (-20.1 to 40.6)	18.9 (1.6 to 40.5)
	YLLs	1.33 (0.61 to 4.31)	2.11 (0.73 to 7.94)	0.51 (0.33 to 0.76)	1.85 (1.23 to 2.41)	2.42 (1.31 to 3.46)	1.26 (0.94 to 1.66)	39.1 (-57.6 to 235.2)	14.9 (-71.5 to 273.5)	144.3 (52.3 to 318.4)
	YLDs	10.29 (6.98 to 14.4)	14.33 (9.78 to 20.22)	6.35 (4.18 to 8.93)	11.49 (7.78 to 15.84)	16.21 (10.82 to 22.37)	6.9 (4.6 to 9.78)	11.7 (-2.1 to 27.9)	13.1 (-5.5 to 35.5)	8.7 (-8.6 to 28.4)
Sistan and Baluchistan	Incidence	3.44 (3.06 to 3.86)	4.73 (4.21 to 5.34)	2.18 (1.92 to 2.5)	3.71 (3.3 to 4.16)	5.12 (4.54 to 5.77)	2.32 (2.05 to 2.62)	7.9 (3.5 to 12.2)	8.3 (2.9 to 14.6)	6.1 (1.1 to 11.4)
	Prevalence	67.54 (60.2 to 76.04)	97.06 (86.1 to 109.15)	41.72 (36.6 to 47.28)	74.31 (65.96 to 83.73)	104.65 (92.49 to 118.26)	44.11 (38.86 to 49.75)	10 (5.7 to 15.2)	7.8 (2 to 14.4)	5.7 (0.6 to 11)
	Deaths	0.06 (0.03 to 0.16)	0.1 (0.04 to 0.32)	0.03 (0.02 to 0.05)	0.12 (0.08 to 0.15)	0.13 (0.06 to 0.19)	0.11 (0.07 to 0.14)	86.5 (-36.3 to 295.4)	29.1 (-69.4 to 315.2)	227.3 (94.3 to 525.4)
	DALYs	10.45 (7.23 to 14.54)	15.02 (10.19 to 21.52)	6.49 (4.47 to 8.88)	12.97 (9.56 to 16.9)	17.06 (12.3 to 22.87)	8.9 (6.65 to 11.59)	24.1 (-2.5 to 48.6)	13.6 (-20.1 to 45.1)	37.1 (13.8 to 67.1)
	YLLs	1.29 (0.68 to 3.36)	2.07 (0.83 to 6.63)	0.68 (0.39 to 1.03)	2.91 (1.88 to 3.79)	3.1 (1.29 to 4.49)	2.72 (1.93 to 3.68)	125 (-24.7 to 381.2)	49.6 (-67.9 to 372.3)	301.4 (137.3 to 674.7)
	YLDs	9.15 (6.14 to 12.81)	12.94 (8.63 to 18.34)	5.81 (3.86 to 8.23)	10.06 (6.85 to 13.72)	13.96 (9.4 to 19.17)	6.17 (4.17 to 8.65)	9.9 (-5.9 to 26.7)	7.9 (-11.6 to 31)	6.2 (-10.3 to 24.4)

Province	Measure	Age-standardized rate (per 100,000)						% Change (1990 to 2019)		
		1990			2019					
		Both	Female	Male	Both	Female	Male	Both	Female	Male
South Khorasan	Incidence	3.72 (3.29 to 4.19)	5.1 (4.5 to 5.74)	2.33 (2.04 to 2.64)	4.04 (3.59 to 4.55)	5.66 (5.02 to 6.38)	2.5 (2.21 to 2.84)	8.7 (4 to 13.6)	11 (4.4 to 17.6)	7.6 (1.9 to 13.6)
	Prevalence	74.39 (65.77 to 83.23)	105.35 (92.83 to 118.13)	44.77 (39.35 to 50.8)	82.7 (73.37 to 93.21)	116.81 (103.61 to 131.63)	48.14 (42.31 to 54.37)	11.2 (5.9 to 16.6)	10.9 (4.2 to 18.1)	7.5 (2.5 to 13.7)
	Deaths	0.07 (0.05 to 0.14)	0.11 (0.05 to 0.25)	0.04 (0.03 to 0.06)	0.09 (0.05 to 0.12)	0.12 (0.04 to 0.17)	0.06 (0.05 to 0.08)	20.8 (-47.5 to 129.4)	4.4 (-68.4 to 184.1)	58.8 (4.1 to 162.8)
	DALYs	11.54 (8.07 to 15.57)	16.24 (11.26 to 22.38)	7.07 (4.9 to 9.67)	13 (9.29 to 17.44)	17.87 (12.49 to 24.28)	8.06 (5.79 to 10.81)	12.7 (-5.5 to 30.1)	10 (-12.7 to 34)	14 (-2.4 to 35.2)
	YLLs	1.54 (0.95 to 2.94)	2.31 (1.06 to 5.26)	0.84 (0.55 to 1.2)	1.91 (1.14 to 2.44)	2.41 (0.97 to 3.37)	1.38 (1.03 to 1.81)	23.9 (-44.9 to 124.8)	4 (-67.2 to 162.8)	63.8 (7.3 to 168.3)
	YLDs	10 (6.71 to 13.99)	13.93 (9.32 to 19.36)	6.23 (4.04 to 8.84)	11.09 (7.39 to 15.46)	15.46 (10.22 to 21.48)	6.68 (4.42 to 9.49)	10.9 (-3.3 to 27.6)	11 (-8 to 33.3)	7.2 (-9.3 to 28.8)
Tehran	Incidence	9.36 (8.35 to 10.45)	16.46 (14.65 to 18.4)	2.69 (2.35 to 3.08)	9.45 (8.35 to 10.56)	16.23 (14.36 to 18.2)	2.7 (2.34 to 3.07)	1 (-4.5 to 6.4)	-1.4 (-7.9 to 4.9)	0.3 (-4.9 to 6.4)
	Prevalence	219.79 (196.5 to 243.59)	400.56 (356.44 to 444.04)	51.34 (44.95 to 58.64)	219.66 (195.09 to 243.71)	388.98 (344.41 to 432.86)	51.28 (44.47 to 58.64)	-0.1 (-5.4 to 5.9)	-2.9 (-8.8 to 3.7)	-0.1 (-5.5 to 5.8)
	Deaths	0.36 (0.08 to 0.67)	0.55 (0.05 to 1.14)	0.14 (0.09 to 0.21)	0.28 (0.06 to 0.42)	0.46 (0.03 to 0.76)	0.1 (0.06 to 0.13)	-23.2 (-54.9 to 32.6)	-16.1 (-68 to 68.2)	-32.7 (-59.1 to 19.7)
	DALYs	37.42 (25.44 to 51.07)	65.58 (43.36 to 90.07)	10.78 (7.69 to 14.22)	35.71 (24.71 to 47.28)	62.11 (41.7 to 83.62)	9.51 (6.89 to 12.44)	-4.6 (-19.7 to 9.5)	-5.3 (-22.2 to 11.3)	-11.8 (-28.5 to 10.2)
	YLLs	8.35 (2 to 15.79)	12.8 (0.96 to 27.41)	3.78 (2.15 to 5.64)	6.61 (1.37 to 10.19)	10.82 (0.6 to 18)	2.5 (1.61 to 3.4)	-20.8 (-54.1 to 32.5)	-15.5 (-65.4 to 62.9)	-33.9 (-60.5 to 20.4)
	YLDs	29.07 (19.48 to 39.9)	52.78 (35.36 to 72.42)	7.01 (4.58 to 9.9)	29.09 (19.57 to 39.63)	51.29 (34.25 to 70.45)	7.01 (4.57 to 9.83)	0.1 (-9.9 to 12.9)	-2.8 (-13.7 to 10.6)	0.1 (-17.7 to 21.7)

Province	Measure	Age-standardized rate (per 100,000)						% Change (1990 to 2019)		
		1990			2019					
		Both	Female	Male	Both	Female	Male	Both	Female	Male
West Azarbayejan	Incidence	3.68 (3.24 to 4.14)	5.08 (4.49 to 5.71)	2.31 (2.01 to 2.61)	4.01 (3.55 to 4.54)	5.61 (4.96 to 6.36)	2.47 (2.17 to 2.81)	9 (4.5 to 13.5)	10.6 (4.5 to 16.6)	7 (1.8 to 11.9)
	Prevalence	73.91 (65.65 to 83.13)	105.05 (93.23 to 118.5)	44.38 (38.84 to 50.58)	81.44 (72.16 to 91.99)	115.86 (102.36 to 131.03)	47.47 (41.71 to 53.87)	10.2 (5.5 to 15.3)	10.3 (3.9 to 16.9)	7 (1.2 to 12.5)
	Deaths	0.06 (0.03 to 0.17)	0.1 (0.04 to 0.3)	0.03 (0.02 to 0.04)	0.09 (0.05 to 0.11)	0.12 (0.05 to 0.17)	0.05 (0.04 to 0.08)	34.4 (-57 to 195.8)	14.1 (-73.3 to 233.7)	97.2 (27.4 to 261.1)
	DALYs	11.27 (7.89 to 15.29)	16.05 (11.02 to 22.63)	6.75 (4.73 to 9.27)	12.71 (8.9 to 16.99)	17.71 (12.36 to 24)	7.73 (5.42 to 10.45)	12.8 (-9.3 to 32.2)	10.3 (-18.3 to 36.7)	14.5 (-2.7 to 35.6)
	YLLs	1.3 (0.71 to 3.32)	2.1 (0.89 to 6.32)	0.56 (0.37 to 0.79)	1.75 (1.07 to 2.24)	2.34 (1.05 to 3.26)	1.12 (0.83 to 1.54)	35 (-55.2 to 182.8)	11.2 (-72.5 to 216.8)	101.4 (30.8 to 257.7)
	YLDs	9.97 (6.72 to 13.75)	13.94 (9.12 to 19.48)	6.2 (4.19 to 8.7)	10.96 (7.24 to 15.3)	15.37 (10.24 to 21.7)	6.61 (4.26 to 9.32)	9.9 (-4.3 to 26.6)	10.2 (-9.3 to 33.8)	6.7 (-9.7 to 26.2)
Yazd	Incidence	3.79 (3.37 to 4.26)	5.32 (4.72 to 6.01)	2.41 (2.12 to 2.73)	4.26 (3.77 to 4.8)	6 (5.29 to 6.78)	2.63 (2.31 to 2.97)	12.4 (7.8 to 17.1)	12.8 (6.2 to 19.5)	9.2 (3.8 to 15.2)
	Prevalence	77.59 (69.33 to 87.37)	110.42 (97.72 to 124.55)	46.65 (40.99 to 53)	86.5 (76.44 to 96.77)	124.72 (110.42 to 139.99)	51 (44.77 to 57.73)	11.5 (6.7 to 16.4)	13 (6.1 to 19.7)	9.3 (3.9 to 15.2)
	Deaths	0.06 (0.03 to 0.14)	0.09 (0.04 to 0.24)	0.03 (0.02 to 0.04)	0.09 (0.04 to 0.12)	0.11 (0.03 to 0.17)	0.06 (0.04 to 0.08)	38.3 (-64.7 to 234.8)	26.1 (-81.6 to 309.5)	120.5 (32.4 to 319.8)
	DALYs	11.66 (7.96 to 15.83)	16.48 (11.14 to 22.85)	7.03 (4.74 to 9.65)	13.42 (9.54 to 17.88)	18.97 (13.26 to 25.57)	8.23 (5.71 to 11.1)	15.1 (-5.7 to 35.6)	15.2 (-13.5 to 42)	17.2 (0 to 39.1)
	YLLs	1.21 (0.61 to 2.78)	1.82 (0.74 to 4.83)	0.53 (0.34 to 0.75)	1.78 (0.85 to 2.4)	2.39 (0.6 to 3.55)	1.18 (0.82 to 1.66)	47.1 (-61.9 to 236.1)	31.4 (-80.4 to 311.7)	124.5 (34.3 to 317.3)
	YLDs	10.45 (6.97 to 14.53)	14.66 (9.7 to 20.87)	6.5 (4.21 to 9.12)	11.64 (7.89 to 16.02)	16.59 (11.2 to 23.16)	7.05 (4.6 to 9.82)	11.4 (-2.5 to 29.2)	13.1 (-5.9 to 37.9)	8.5 (-8.5 to 27.8)

Province	Measure	Age-standardized rate (per 100,000)						% Change (1990 to 2019)		
		1990			2019					
		Both	Female	Male	Both	Female	Male	Both	Female	Male
Zanjan	Incidence	3.71 (3.3 to 4.18)	5.09 (4.54 to 5.75)	2.33 (2.04 to 2.65)	4.07 (3.6 to 4.6)	5.69 (5.03 to 6.45)	2.51 (2.21 to 2.84)	9.6 (4.7 to 13.9)	11.8 (5.2 to 18.4)	7.8 (2.3 to 13.6)
	Prevalence	74.69 (66.34 to 83.78)	105.42 (93.41 to 118.61)	44.76 (39.33 to 50.63)	82.97 (73.7 to 93.4)	117.8 (104.35 to 132.8)	48.38 (42.72 to 54.64)	11.1 (6 to 16.3)	11.7 (5.2 to 18.8)	8.1 (2.2 to 14.1)
	Deaths	0.05 (0.02 to 0.17)	0.08 (0.03 to 0.3)	0.02 (0.01 to 0.03)	0.08 (0.05 to 0.1)	0.11 (0.05 to 0.15)	0.05 (0.04 to 0.07)	57.8 (-57.6 to 303.7)	32.8 (-70.7 to 361.3)	171.5 (67.1 to 399.8)
	DALYs	11.09 (7.55 to 15.32)	15.71 (10.63 to 22.07)	6.61 (4.49 to 9.13)	12.83 (9.15 to 17.02)	17.93 (12.52 to 23.99)	7.72 (5.35 to 10.52)	15.7 (-7.7 to 35.4)	14.1 (-16.4 to 39.8)	16.8 (-1.7 to 38.5)
	YLLs	1.02 (0.46 to 3.23)	1.7 (0.59 to 6.14)	0.38 (0.26 to 0.57)	1.66 (1.02 to 2.13)	2.25 (0.93 to 3.08)	1.03 (0.76 to 1.48)	62.4 (-54 to 295)	31.8 (-70 to 344.9)	168.8 (66.7 to 394.5)
	YLDs	10.06 (6.75 to 14.07)	14.01 (9.27 to 19.57)	6.22 (4.1 to 8.8)	11.17 (7.48 to 15.28)	15.69 (10.45 to 21.62)	6.69 (4.34 to 9.47)	11 (-3 to 26.8)	12 (-7 to 33.5)	7.5 (-11.2 to 27.8)

Data in parentheses are 95% Uncertainty Intervals (95% UIs)
