# Supplementary material for: National and subnational burden and attributable risk factors of osteoarthritis, rheumatoid arthritis, and low back pain in Iran: 1990–2019 findings of the Global Burden of Disease (GBD) Study
Source: PLoS One. 2026 Jul 6;21(7):e0344038. doi: 10.1371/journal.pone.0344038 (PMC13336167; doi:10.1371/journal.pone.0344038)
Supplement: S3 Table — (PDF) [file pone.0344038.s003.pdf]

S3 Table. All ages number and age-standardized rate of incidence, prevalence, and YLDs of OA in 1990 and 2019

Province	Measure	Age (metric)	Year						% Change (1990 to 2019)		
			1990			2019					
			Both	Female	Male	Both	Female	Male	Both	Female	Male
Alborz	Incidence	All ages (number)	3045 (2677 to 3446)	1579 (1380 to 1794)	1466 (1284 to 1657)	14192 (12482 to 16088)	7844 (6902 to 8910)	6349 (5607 to 7192)	366.1 (350.5 to 383.9)	396.9 (375.7 to 419.3)	333.1 (312 to 357.3)
		Age-standardized (rate per 100,000)	415.5 (368.7 to 468)	466.8 (411.1 to 529.9)	371.8 (328.8 to 415.9)	442.2 (392.5 to 499.4)	497.5 (439.9 to 564)	389.3 (343.1 to 439.7)	6.4 (3.3 to 10.2)	6.6 (2.3 to 11.5)	4.7 (0 to 10)
	Prevalence	All ages (number)	31446 (27913 to 35306)	16415 (14435 to 18528)	15032 (13274 to 16952)	150415 (134220 to 168595)	83414 (73811 to 93833)	67001 (59939 to 75281)	378.3 (363.3 to 395.4)	408.2 (389.1 to 430)	345.7 (324.5 to 368.3)
		Age-standardized (rate per 100,000)	5221.2 (4680.9 to 5806.8)	5884.2 (5234.5 to 6625.8)	4632 (4132.8 to 5148)	5512.9 (4946.8 to 6138.5)	6235.4 (5566.8 to 6977.6)	4826.2 (4313.8 to 5382.7)	5.6 (2.6 to 9)	6 (2.4 to 10.1)	4.2 (-0.1 to 9)
	YLDs	All ages (number)	1092 (548 to 2138)	573 (289 to 1120)	519 (259 to 1018)	5246 (2646 to 10411)	2927 (1476 to 5827)	2318 (1174 to 4529)	380.2 (362.3 to 400.8)	410.9 (387.4 to 438.7)	346.4 (322.4 to 373)
		Age-standardized (rate per 100,000)	181.5 (92 to 355.5)	205.9 (103.6 to 401.7)	159.7 (79.9 to 317.2)	192.6 (97.2 to 377.2)	219.5 (111.5 to 431)	167 (84 to 329.2)	6.1 (2.7 to 10.2)	6.6 (2.3 to 11.7)	4.5 (-0.5 to 9.9)
Ardebil	Incidence	All ages (number)	2408 (2106 to 2726)	1282 (1121 to 1456)	1127 (988 to 1276)	5918 (5221 to 6706)	3327 (2938 to 3783)	2591 (2277 to 2927)	145.7 (135.4 to 155.9)	159.6 (147.8 to 172.1)	130 (115.8 to 144)
		Age-standardized (rate per 100,000)	403 (357.3 to 453.5)	452.1 (398.2 to 514)	358.2 (319.4 to 403.3)	436 (387.3 to 492.2)	489.9 (433.2 to 555.1)	382.3 (338.8 to 431.2)	8.2 (5.2 to 11.5)	8.4 (4.1 to 13.3)	6.7 (2.1 to 10.9)
	Prevalence	All ages (number)	26438 (23496 to 29776)	13791 (12162 to 15609)	12647 (11205 to 14279)	65366 (58428 to 72910)	37463 (33331 to 42191)	27902 (24869 to 31088)	147.2 (138.9 to 155.3)	171.7 (159.6 to 183.3)	120.6 (109.4 to 131)
		Age-standardized (rate per 100,000)	5036.8 (4527.9 to 5624.7)	5714.3 (5073.7 to 6424.6)	4451.2 (3996.8 to 4965.2)	5455.1 (4891.2 to 6068)	6144 (5489.3 to 6899.8)	4735.6 (4237.4 to 5247.5)	8.3 (5.4 to 11.1)	7.5 (3.5 to 11.6)	6.4 (1.8 to 10.4)
	YLDs	All ages (number)	914 (462 to 1782)	479 (239 to 929)	435 (216 to 865)	2276 (1144 to 4474)	1313 (663 to 2546)	963 (481 to 1911)	149.1 (139.1 to 158.3)	174.2 (159.6 to 188.2)	121.4 (108.2 to 134.4)
		Age-standardized (rate per 100,000)	173.8 (87.9 to 338.3)	198.6 (99.8 to 385.3)	152.5 (76.1 to 300.4)	190.4 (95.8 to 376.7)	216 (109.2 to 419.3)	163.6 (82.3 to 325.1)	9.6 (6 to 13.1)	8.7 (3.7 to 13.6)	7.3 (1.6 to 12.3)

Province	Measure	Age (metric)	Year						% Change (1990 to 2019)		
			1990			2019					
			Both	Female	Male	Both	Female	Male	Both	Female	Male
Bushehr	Incidence	All ages (number)	1374 (1214 to 1545)	745 (657 to 850)	629 (554 to 708)	4831 (4261 to 5500)	2612 (2297 to 2984)	2219 (1951 to 2507)	251.6 (237.4 to 265.6)	250.6 (235.2 to 268.2)	252.8 (231.7 to 273.9)
		Age-standardized (rate per 100,000)	409 (364 to 459.9)	457.2 (404.6 to 520.8)	363.3 (322.4 to 407.9)	437.3 (387.6 to 495.6)	493.5 (435.6 to 563.3)	385.6 (341.5 to 433.9)	6.9 (3.5 to 9.9)	7.9 (3.5 to 12.5)	6.1 (1 to 10.8)
	Prevalence	All ages (number)	14669 (12971 to 16457)	7944 (6971 to 9031)	6725 (5904 to 7560)	50246 (45079 to 56327)	27868 (24770 to 31444)	22378 (19984 to 25233)	242.5 (232.1 to 252.8)	250.8 (237.4 to 265.2)	232.8 (216 to 248.8)
		Age-standardized (rate per 100,000)	5129.6 (4585 to 5725.6)	5771.3 (5119.7 to 6518.5)	4518.4 (4030.8 to 5021.8)	5474.3 (4931.1 to 6104.7)	6185.8 (5516.7 to 6928.6)	4777.3 (4262 to 5321.4)	6.7 (3.8 to 9.7)	7.2 (3.4 to 11.3)	5.7 (1.2 to 10.4)
	YLDs	All ages (number)	509 (256 to 996)	277 (141 to 542)	232 (115 to 461)	1750 (879 to 3432)	976 (493 to 1927)	774 (386 to 1522)	243.8 (231.4 to 257)	252.3 (235.3 to 270)	233.6 (214.4 to 253.2)
		Age-standardized (rate per 100,000)	177.7 (89.3 to 348.3)	201.3 (101.8 to 394.9)	155.2 (77.6 to 306)	191.1 (96.8 to 376.7)	217.4 (110.2 to 428.8)	165.2 (83.2 to 326.3)	7.6 (4.1 to 10.9)	8 (3.2 to 12.8)	6.5 (1.3 to 11.9)
Chahar Mahaal and Bakhtiari	Incidence	All ages (number)	1412 (1250 to 1604)	749 (660 to 857)	663 (585 to 752)	4177 (3690 to 4688)	2334 (2054 to 2658)	1842 (1621 to 2074)	195.9 (185.5 to 206.9)	211.7 (197.1 to 226.7)	178 (163.2 to 193.5)
		Age-standardized (rate per 100,000)	403.2 (358.8 to 456.2)	452.2 (402 to 514.2)	358.4 (319.6 to 405)	432.4 (385 to 487)	486 (429.4 to 551.3)	379.6 (337.8 to 424.9)	7.2 (4.2 to 10.8)	7.5 (3.1 to 12.2)	5.9 (1.1 to 10.9)
	Prevalence	All ages (number)	15663 (13919 to 17641)	8214 (7258 to 9291)	7450 (6562 to 8358)	46165 (41425 to 51671)	26263 (23373 to 29643)	19902 (17782 to 22270)	194.7 (186.1 to 204)	219.8 (207.7 to 233.2)	167.1 (153.9 to 180.4)
		Age-standardized (rate per 100,000)	5051 (4525.4 to 5644.7)	5713.3 (5087.1 to 6433.1)	4454.8 (3964.7 to 4967.5)	5413 (4859.2 to 6032.1)	6099.5 (5444.1 to 6851.8)	4706.2 (4206 to 5230)	7.2 (4.4 to 10.2)	6.8 (3 to 10.9)	5.6 (1.2 to 10.5)
	YLDs	All ages (number)	543 (272 to 1067)	287 (144 to 562)	256 (129 to 515)	1614 (808 to 3171)	924 (465 to 1809)	689 (339 to 1379)	197.1 (186.3 to 209.1)	222.6 (207.3 to 239.5)	168.7 (153.7 to 183.8)
		Age-standardized (rate per 100,000)	174.7 (88.5 to 341.5)	199.3 (101.3 to 390.4)	152.6 (76.9 to 304.2)	189.6 (94.8 to 369.4)	215.3 (108.5 to 419.3)	163 (80.4 to 326.5)	8.5 (5.1 to 12.4)	8 (3.3 to 13.1)	6.8 (1.6 to 12.3)

Province	Measure	Age (metric)	Year						% Change (1990 to 2019)		
			1990			2019					
			Both	Female	Male	Both	Female	Male	Both	Female	Male
East Azarbayejan	Incidence	All ages (number)	8168 (7182 to 9241)	4385 (3828 to 5014)	3783 (3313 to 4264)	19847 (17587 to 22434)	11126 (9791 to 12568)	8721 (7726 to 9853)	143 (134.5 to 152.3)	153.7 (141.5 to 166.9)	130.6 (118.2 to 142.7)
		Age-standardized (rate per 100,000)	405.4 (360 to 455.6)	454 (401.9 to 517.2)	360.3 (319.5 to 403.2)	435.6 (388 to 491.1)	489.5 (432.9 to 555.2)	382.3 (339.9 to 430.8)	7.5 (4.2 to 11)	7.8 (3.2 to 12.7)	6.1 (1.9 to 10.6)
	Prevalence	All ages (number)	89513 (79272 to 100960)	47491 (41849 to 53908)	42022 (37384 to 47113)	221875 (197961 to 248010)	125777 (111871 to 141408)	96098 (85071 to 107851)	147.9 (139.9 to 156.1)	164.8 (152.8 to 175.8)	128.7 (119.1 to 138.8)
		Age-standardized (rate per 100,000)	5073.7 (4539.4 to 5652.1)	5735.7 (5095.8 to 6452.8)	4477.6 (4018.2 to 4942.8)	5441.8 (4879.7 to 6054.4)	6140.1 (5481.4 to 6869.4)	4735.3 (4220.3 to 5272.2)	7.3 (4.3 to 10.4)	7.1 (3 to 10.9)	5.8 (1.7 to 10.2)
	YLDs	All ages (number)	3108 (1580 to 6269)	1658 (844 to 3261)	1450 (732 to 2903)	7740 (3894 to 15325)	4420 (2243 to 8629)	3319 (1639 to 6569)	149 (139.7 to 158.3)	166.6 (153.5 to 180.1)	128.9 (117.8 to 141.1)
		Age-standardized (rate per 100,000)	175.8 (89.5 to 349.6)	200.3 (101.9 to 397.2)	153.9 (77.4 to 308)	190 (95.7 to 375.5)	216.1 (109.8 to 425.4)	163.5 (81.5 to 325.4)	8 (4.6 to 11.7)	7.9 (3.3 to 12.9)	6.2 (1.1 to 11.5)
Fars	Incidence	All ages (number)	7736 (6835 to 8734)	4165 (3649 to 4747)	3571 (3151 to 4029)	23324 (20629 to 26306)	13050 (11421 to 14851)	10274 (9094 to 11526)	201.5 (190.7 to 213.2)	213.3 (201 to 228.2)	187.7 (172.7 to 202)
		Age-standardized (rate per 100,000)	409.1 (364.1 to 461)	458 (404.5 to 521.8)	363.3 (323.4 to 408.3)	439.6 (390.1 to 496.5)	494.1 (435.3 to 563.3)	385.9 (344.4 to 432.4)	7.4 (4.4 to 11)	7.9 (3.9 to 12.9)	6.2 (1.6 to 10.6)
	Prevalence	All ages (number)	85389 (76298 to 95516)	45966 (40869 to 52013)	39424 (35072 to 44206)	255938 (228942 to 285399)	144263 (128114 to 162396)	111675 (99754 to 124396)	199.7 (190.4 to 209.3)	213.8 (201.9 to 226.5)	183.3 (170.7 to 195.9)
		Age-standardized (rate per 100,000)	5138 (4626.2 to 5703.7)	5780.8 (5181 to 6524.3)	4521.1 (4058.5 to 5009.1)	5487.4 (4921.6 to 6094.4)	6192.8 (5511.6 to 6944.2)	4784.3 (4282.5 to 5290.5)	6.8 (4 to 9.6)	7.1 (3.4 to 11.1)	5.8 (1.8 to 9.7)
	YLDs	All ages (number)	2955 (1500 to 5782)	1601 (811 to 3176)	1354 (680 to 2649)	8916 (4484 to 17590)	5062 (2541 to 9930)	3855 (1924 to 7529)	201.8 (191.4 to 212.9)	216.2 (202.4 to 231.5)	184.7 (169.1 to 200.2)
		Age-standardized (rate per 100,000)	177.2 (89.6 to 347.4)	201 (102.1 to 398.9)	154.4 (77.3 to 305.5)	191.5 (96.7 to 374.4)	217.8 (109.7 to 427.7)	165.2 (83.2 to 323.7)	8.1 (4.6 to 11.4)	8.4 (3.8 to 13)	7 (1.7 to 11.9)

Province	Measure	Age (metric)	Year						% Change (1990 to 2019)		
			1990			2019					
			Both	Female	Male	Both	Female	Male	Both	Female	Male
Gilan	Incidence	All ages (number)	6068 (5312 to 6919)	3344 (2926 to 3813)	2724 (2394 to 3116)	15105 (13303 to 17029)	8562 (7502 to 9763)	6543 (5747 to 7383)	148.9 (140.8 to 157.3)	156.1 (145.6 to 168)	140.2 (127.9 to 151.7)
		Age-standardized (rate per 100,000)	411.8 (365.1 to 466)	458.6 (404.7 to 520.8)	365.4 (323.7 to 413.9)	440.9 (390.4 to 496.4)	494.4 (437.4 to 561.2)	386.4 (341.7 to 434)	7.1 (4.1 to 10.3)	7.8 (3.6 to 12.4)	5.7 (0.9 to 10.5)
	Prevalence	All ages (number)	68158 (60655 to 76624)	37987 (33657 to 43289)	30171 (26621 to 33920)	177821 (159785 to 198324)	101496 (90710 to 114344)	76326 (68261 to 85193)	160.9 (153.2 to 169.5)	167.2 (157.1 to 178.2)	153 (141.6 to 165.1)
		Age-standardized (rate per 100,000)	5182.8 (4648.6 to 5778.8)	5785 (5163.1 to 6521.9)	4544.4 (4057.5 to 5053)	5501.6 (4960.1 to 6109.1)	6196.2 (5535 to 6960.3)	4788.8 (4294.1 to 5322.6)	6.2 (3.3 to 9.2)	7.1 (3.3 to 11.2)	5.4 (1.3 to 9.6)
	YLDs	All ages (number)	2367 (1185 to 4654)	1327 (674 to 2628)	1040 (517 to 2064)	6196 (3122 to 12204)	3563 (1804 to 7016)	2633 (1318 to 5245)	161.7 (152.4 to 171.7)	168.4 (156.3 to 181.6)	153.2 (140.1 to 167.3)
		Age-standardized (rate per 100,000)	179.5 (90.8 to 351.5)	201.8 (102.3 to 398.4)	155.8 (77.7 to 307.9)	191.8 (96.5 to 376.9)	217.7 (109.9 to 428.3)	165.2 (82.7 to 331)	6.8 (3.4 to 10.5)	7.9 (3.4 to 12.9)	6 (1.2 to 11.5)
Golestan	Incidence	All ages (number)	2713 (2386 to 3079)	1467 (1279 to 1678)	1246 (1100 to 1408)	8244 (7277 to 9352)	4702 (4134 to 5392)	3542 (3142 to 4010)	203.9 (193.5 to 214.6)	220.6 (206.8 to 235.5)	184.2 (169.9 to 198.9)
		Age-standardized (rate per 100,000)	408 (362.9 to 460.9)	456.4 (400.9 to 521.3)	362.4 (323.2 to 406.7)	438.3 (389.5 to 496)	491.2 (434.2 to 559.9)	383.9 (340.8 to 433.4)	7.4 (4 to 10.8)	7.6 (3.3 to 12.2)	5.9 (1.5 to 10.8)
	Prevalence	All ages (number)	29230 (26034 to 33049)	15691 (13834 to 17899)	13539 (11979 to 15192)	87597 (77857 to 98486)	50451 (44782 to 56929)	37147 (33014 to 41664)	199.7 (190.3 to 209.3)	221.5 (208.6 to 235)	174.4 (162.1 to 186.6)
		Age-standardized (rate per 100,000)	5119.5 (4598 to 5737.6)	5766.8 (5122.9 to 6523.7)	4506.3 (4035.5 to 5016.7)	5474.8 (4894.6 to 6096)	6156.6 (5517.6 to 6919.9)	4756.5 (4261 to 5279.1)	6.9 (3.9 to 9.9)	6.8 (3 to 10.7)	5.6 (1.3 to 10)
	YLDs	All ages (number)	1014 (514 to 1991)	547 (276 to 1080)	467 (236 to 933)	3048 (1539 to 5954)	1767 (892 to 3476)	1281 (644 to 2533)	200.5 (189.9 to 212.2)	223 (207.3 to 239.2)	174.2 (159.9 to 189.7)
		Age-standardized (rate per 100,000)	177.4 (90.7 to 349.6)	201.3 (102 to 397.9)	154.9 (78.8 to 309.8)	190.9 (96.4 to 373.5)	216.3 (109 to 422.7)	164 (82.6 to 322)	7.6 (4.1 to 11.4)	7.5 (2.7 to 12.4)	5.8 (1 to 11.4)

Province	Measure	Age (metric)	Year						% Change (1990 to 2019)		
			1990			2019					
			Both	Female	Male	Both	Female	Male	Both	Female	Male
Hamadan	Incidence	All ages (number)	3818 (3355 to 4319)	2068 (1814 to 2360)	1749 (1542 to 1981)	8544 (7585 to 9636)	4801 (4243 to 5447)	3742 (3316 to 4223)	123.8 (116.3 to 131.3)	132.1 (123.3 to 142.4)	113.9 (102.8 to 124.7)
		Age-standardized (rate per 100,000)	407.1 (360.4 to 459)	455.4 (403.1 to 518.8)	361.4 (321.4 to 407)	437.3 (389.1 to 493)	491.4 (433.3 to 557)	383.5 (340.8 to 431.8)	7.4 (4.3 to 10.8)	7.9 (3.9 to 12.3)	6.1 (1.3 to 10.9)
	Prevalence	All ages (number)	42903 (38192 to 48246)	22985 (20450 to 25997)	19919 (17570 to 22216)	98838 (88566 to 109912)	56031 (50120 to 62885)	42807 (38317 to 47790)	130.4 (123.8 to 137.5)	143.8 (135.3 to 154.2)	114.9 (104.9 to 124.3)
		Age-standardized (rate per 100,000)	5096.9 (4563.6 to 5700)	5749.4 (5157.1 to 6487.2)	4493.6 (4000.8 to 4981.9)	5465.9 (4903.2 to 6082)	6161.5 (5511.5 to 6912.9)	4759.7 (4264.4 to 5301)	7.2 (4.5 to 10.3)	7.2 (3.6 to 11.3)	5.9 (1.4 to 10.3)
	YLDs	All ages (number)	1487 (749 to 2907)	801 (404 to 1566)	685 (342 to 1378)	3453 (1741 to 6756)	1973 (995 to 3840)	1481 (747 to 2915)	132.3 (124.9 to 140.4)	146.2 (135.9 to 158.5)	116.1 (105 to 127.1)
		Age-standardized (rate per 100,000)	176.1 (89.1 to 346.4)	200.2 (100.8 to 394.8)	153.8 (77.1 to 309.9)	191.3 (96.3 to 374.1)	217.3 (109.6 to 424)	164.8 (83 to 327.5)	8.7 (5.3 to 12.2)	8.5 (4.4 to 13.7)	7.1 (1.9 to 12.2)
Hormozgan	Incidence	All ages (number)	1782 (1576 to 2010)	920 (811 to 1043)	862 (757 to 977)	6493 (5758 to 7364)	3602 (3172 to 4111)	2891 (2553 to 3264)	264.3 (249.1 to 280.6)	291.6 (274.4 to 309.8)	235.2 (214.4 to 255.8)
		Age-standardized (rate per 100,000)	401.5 (357.5 to 453.3)	451.3 (398.5 to 511.5)	358.4 (316.8 to 403.2)	435.1 (386.3 to 491.8)	489.1 (432.7 to 557.6)	382.5 (338.9 to 431.2)	8.4 (5.6 to 11.4)	8.4 (4.2 to 12.5)	6.7 (2.2 to 11.7)
	Prevalence	All ages (number)	20107 (17943 to 22556)	10365 (9211 to 11707)	9742 (8621 to 10928)	66765 (59808 to 74441)	37583 (33487 to 42360)	29182 (26078 to 32494)	232.1 (221.4 to 243.1)	262.6 (248.1 to 278.3)	199.6 (183.5 to 216.4)
		Age-standardized (rate per 100,000)	5036 (4513.3 to 5621.6)	5701.4 (5093.2 to 6417.6)	4452.1 (3989.7 to 4965.7)	5441.8 (4898.4 to 6061.7)	6137.3 (5495.4 to 6864.7)	4740.2 (4250.4 to 5249.5)	8.1 (5.3 to 11.1)	7.6 (3.9 to 11.8)	6.5 (2.1 to 11.2)
	YLDs	All ages (number)	697 (352 to 1383)	361 (181 to 705)	336 (168 to 651)	2325 (1178 to 4583)	1318 (666 to 2619)	1007 (504 to 1984)	233.7 (221.7 to 246.7)	264.9 (248 to 282.8)	200 (181.6 to 219.7)
		Age-standardized (rate per 100,000)	174.2 (87.8 to 344.9)	198.5 (99.9 to 388.9)	152.8 (76.7 to 297.9)	190.1 (96.4 to 377.9)	216.1 (109.6 to 427.2)	163.8 (81.9 to 328.4)	9.2 (5.7 to 12.8)	8.9 (4.2 to 13.6)	7.2 (2 to 13.1)

Province	Measure	Age (metric)	Year						% Change (1990 to 2019)		
			1990			2019					
			Both	Female	Male	Both	Female	Male	Both	Female	Male
Ilam	Incidence	All ages (number)	792 (700 to 899)	399 (352 to 458)	393 (344 to 445)	2617 (2328 to 2964)	1472 (1303 to 1674)	1145 (1015 to 1290)	230.2 (218 to 243.5)	268.6 (251.4 to 286.1)	191.2 (174 to 210.6)
		Age-standardized (rate per 100,000)	403.2 (358.1 to 455.5)	454.4 (402.7 to 521.2)	361.4 (320.8 to 405.5)	438.9 (389.8 to 492.7)	493 (436 to 558.5)	384.8 (343.3 to 433.1)	8.9 (5.9 to 12.2)	8.5 (4 to 13.1)	6.5 (1.8 to 11.3)
	Prevalence	All ages (number)	8571 (7638 to 9612)	4182 (3693 to 4773)	4389 (3901 to 4937)	27359 (24598 to 30672)	15410 (13750 to 17294)	11949 (10701 to 13299)	219.2 (209.7 to 229.5)	268.5 (254.1 to 283.4)	172.3 (159.2 to 186.2)
		Age-standardized (rate per 100,000)	5033.7 (4521.5 to 5621.6)	5742.6 (5118.6 to 6505.2)	4495.5 (4035.4 to 5018.5)	5475.2 (4930.3 to 6087.5)	6180.7 (5549.1 to 6932.4)	4772.5 (4275.2 to 5276.9)	8.8 (5.8 to 11.8)	7.6 (3.7 to 11.6)	6.2 (1.7 to 10.6)
	YLDs	All ages (number)	297 (150 to 590)	146 (73 to 286)	152 (76 to 302)	954 (481 to 1893)	540 (272 to 1063)	414 (207 to 813)	220.9 (209.1 to 232.7)	271 (254.2 to 289.5)	172.7 (157.8 to 187.9)
		Age-standardized (rate per 100,000)	174.5 (88.2 to 344.2)	200.6 (101 to 392.8)	154.9 (78.2 to 309.3)	191.5 (96.8 to 377.3)	217.6 (109.8 to 427.7)	165.4 (82.8 to 324)	9.7 (6.2 to 13.4)	8.5 (3.9 to 13.5)	6.8 (1.5 to 11.9)
Isfahan	Incidence	All ages (number)	8868 (7824 to 10083)	4776 (4192 to 5463)	4092 (3606 to 4628)	26495 (23368 to 29802)	14692 (12914 to 16640)	11803 (10479 to 13219)	198.8 (188.7 to 208.9)	207.6 (194.7 to 220.9)	188.5 (174.4 to 202.6)
		Age-standardized (rate per 100,000)	411.3 (365.6 to 465.8)	459.8 (405 to 523.3)	365.9 (324.4 to 412.4)	438.5 (388.1 to 491.9)	493.6 (436 to 557.6)	385.2 (342.6 to 430.5)	6.6 (3.1 to 9.9)	7.4 (2.8 to 11.8)	5.3 (0.7 to 9.8)
	Prevalence	All ages (number)	98828 (88388 to 110324)	53905 (47839 to 61061)	44923 (39895 to 50127)	298375 (267635 to 332629)	166160 (147775 to 186141)	132215 (118981 to 146318)	201.9 (192 to 211.3)	208.2 (195.6 to 221.5)	194.3 (181 to 208.8)
		Age-standardized (rate per 100,000)	5175.2 (4663.4 to 5752)	5803.4 (5179.6 to 6530.4)	4554.7 (4079.8 to 5037.8)	5469.6 (4922 to 6045.9)	6186.1 (5523.6 to 6910.4)	4774.8 (4300.9 to 5270.6)	5.7 (2.5 to 8.7)	6.6 (2.6 to 10.8)	4.8 (0.7 to 9.6)
	YLDs	All ages (number)	3437 (1726 to 6691)	1886 (940 to 3698)	1551 (778 to 3038)	10405 (5265 to 20461)	5835 (2965 to 11371)	4570 (2272 to 9126)	202.7 (191.7 to 214)	209.4 (195.5 to 225.1)	194.6 (179.7 to 212)
		Age-standardized (rate per 100,000)	179.8 (90.4 to 349.9)	202.9 (101.6 to 399.1)	156.8 (79 to 308.8)	190.9 (96.4 to 374.7)	217.6 (110.8 to 425.4)	165 (82.3 to 328)	6.2 (2.6 to 9.9)	7.2 (2.6 to 12.1)	5.3 (0.6 to 11)

Province	Measure	Age (metric)	Year						% Change (1990 to 2019)		
			1990			2019					
			Both	Female	Male	Both	Female	Male	Both	Female	Male
Kerman	Incidence	All ages (number)	3970 (3493 to 4498)	2129 (1869 to 2424)	1841 (1609 to 2085)	13129 (11669 to 14809)	7280 (6426 to 8285)	5849 (5188 to 6578)	230.7 (217.8 to 243.9)	241.9 (227 to 259.1)	217.8 (199.7 to 234.7)
		Age-standardized (rate per 100,000)	410.3 (364.6 to 462.7)	459.3 (405.8 to 522)	364.7 (323.4 to 409.3)	435.3 (386.3 to 490.7)	490.1 (433.3 to 557.7)	382.3 (339.6 to 429)	6.1 (2.9 to 9.6)	6.7 (2.1 to 11.9)	4.8 (0.5 to 9.2)
	Prevalence	All ages (number)	44614 (39715 to 49943)	23855 (21077 to 27097)	20759 (18426 to 23251)	141708 (127210 to 158396)	79425 (70698 to 89399)	62283 (55539 to 69440)	217.6 (208.2 to 228.3)	232.9 (219.7 to 248.2)	200 (185.5 to 214.1)
		Age-standardized (rate per 100,000)	5143.9 (4605.1 to 5748.9)	5791.7 (5148.7 to 6538.8)	4532.9 (4047.5 to 5022.1)	5435.7 (4895.9 to 6036.6)	6140.8 (5481.4 to 6941.2)	4737.6 (4232.5 to 5260.9)	5.7 (2.9 to 8.6)	6 (2.1 to 10.6)	4.5 (0.2 to 8.7)
	YLDs	All ages (number)	1543 (777 to 3065)	830 (417 to 1633)	712 (354 to 1405)	4907 (2476 to 9734)	2770 (1399 to 5498)	2136 (1070 to 4247)	218.1 (206.4 to 229.8)	233.7 (217.7 to 250.8)	199.9 (182.9 to 216.8)
		Age-standardized (rate per 100,000)	177.2 (88.8 to 348.7)	201.2 (101.1 to 395.2)	154.7 (77.4 to 305.5)	188.6 (95.6 to 370.6)	214.8 (109 to 424.3)	162.5 (81.7 to 322.9)	6.4 (3 to 9.9)	6.8 (2.1 to 12)	5.1 (-0.2 to 10.1)
Kermanshah	Incidence	All ages (number)	3543 (3114 to 4008)	1856 (1625 to 2123)	1687 (1485 to 1902)	9503 (8406 to 10739)	5408 (4757 to 6157)	4095 (3622 to 4619)	168.2 (159.9 to 177.5)	191.3 (180 to 204.7)	142.7 (129.6 to 155.8)
		Age-standardized (rate per 100,000)	404.2 (360.4 to 454.3)	454.6 (403 to 519.3)	359.8 (320 to 403)	436.4 (388.1 to 492.7)	489.1 (431.6 to 557.6)	382.2 (339.1 to 430.3)	8 (5 to 11)	7.6 (3.7 to 11.9)	6.2 (1.2 to 11.5)
	Prevalence	All ages (number)	38827 (34515 to 43426)	19813 (17581 to 22370)	19014 (16852 to 21306)	106346 (95511 to 119058)	60804 (54105 to 68312)	45542 (40820 to 50830)	173.9 (166.3 to 182.5)	206.9 (195.9 to 219.6)	139.5 (127.4 to 151.2)
		Age-standardized (rate per 100,000)	5046.5 (4529.5 to 5615.4)	5736 (5119.7 to 6456)	4473.9 (4006.5 to 4984.6)	5443.5 (4896.2 to 6062.6)	6132 (5482.4 to 6904)	4734.7 (4249.8 to 5252.7)	7.9 (5.3 to 10.8)	6.9 (3.5 to 11)	5.8 (1 to 10.5)
	YLDs	All ages (number)	1345 (680 to 2644)	690 (349 to 1360)	655 (327 to 1295)	3707 (1869 to 7222)	2134 (1072 to 4192)	1573 (788 to 3070)	175.6 (166.2 to 185.2)	209.2 (196.4 to 224)	140.1 (126.8 to 153.9)
		Age-standardized (rate per 100,000)	174.6 (88.6 to 341.9)	200.1 (101.3 to 394.8)	153.6 (77.3 to 304.3)	190 (95.8 to 369.8)	215.6 (108.6 to 421.7)	163.6 (82.2 to 322)	8.8 (5.6 to 12.2)	7.8 (3.7 to 12.6)	6.5 (1 to 11.9)

Province	Measure	Age (metric)	Year						% Change (1990 to 2019)		
			1990			2019					
			Both	Female	Male	Both	Female	Male	Both	Female	Male
Khorasan-e-Razavi	Incidence	All ages (number)	10507 (9273 to 11880)	5653 (4947 to 6460)	4854 (4298 to 5481)	28107 (24930 to 31761)	15876 (13957 to 17996)	12230 (10845 to 13786)	167.5 (157.1 to 178)	180.8 (169.2 to 194.8)	152 (137.6 to 167.1)
		Age-standardized (rate per 100,000)	406.3 (361.8 to 456.1)	454.6 (401.2 to 517.2)	361 (322 to 405.2)	435.5 (386.7 to 490.9)	488.9 (431.5 to 554.8)	381.6 (339 to 428.6)	7.2 (4.2 to 10.4)	7.5 (3.3 to 12.6)	5.7 (1 to 10.7)
	Prevalence	All ages (number)	116823 (104454 to 131205)	62269 (54996 to 70715)	54553 (48301 to 61294)	306647 (274142 to 342174)	175133 (156439 to 196773)	131514 (117106 to 146693)	162.5 (153.8 to 171)	181.3 (170.9 to 193)	141.1 (129.8 to 153.1)
		Age-standardized (rate per 100,000)	5090.1 (4568.6 to 5679.7)	5743.5 (5127.7 to 6462)	4488.2 (4016.4 to 4996.2)	5437.7 (4883.9 to 6043.3)	6126.8 (5492.2 to 6860.2)	4726.8 (4223.8 to 5238.2)	6.8 (3.9 to 9.8)	6.7 (3.2 to 10.7)	5.3 (1.3 to 10)
	YLDs	All ages (number)	4035 (2046 to 8028)	2163 (1084 to 4240)	1871 (946 to 3752)	10604 (5322 to 21234)	6099 (3066 to 11982)	4505 (2247 to 9017)	162.8 (152.7 to 172.2)	182 (169 to 195.7)	140.7 (127.9 to 153.9)
		Age-standardized (rate per 100,000)	175.3 (88.5 to 346.7)	199.3 (101.4 to 391)	153.2 (78.3 to 309.1)	188.2 (95 to 373.5)	213.8 (108.1 to 421.1)	161.8 (80.9 to 323)	7.4 (3.9 to 10.8)	7.3 (2.8 to 12.1)	5.6 (0.5 to 10.5)
Khuzestan	Incidence	All ages (number)	5982 (5261 to 6760)	3219 (2840 to 3676)	2763 (2434 to 3129)	19153 (17016 to 21548)	10686 (9416 to 12088)	8466 (7470 to 9502)	220.2 (209 to 230.9)	232 (218.7 to 245.3)	206.4 (192.2 to 221.8)
		Age-standardized (rate per 100,000)	406 (360.2 to 457)	454.5 (400.5 to 517.2)	361.1 (320.3 to 407)	436.1 (387.9 to 490.8)	490.3 (434.7 to 556.2)	383.1 (338.5 to 430.3)	7.4 (4.1 to 10.5)	7.9 (3.6 to 12)	6.1 (1.5 to 10.7)
	Prevalence	All ages (number)	65713 (58444 to 73709)	35620 (31681 to 40396)	30093 (26501 to 33749)	204693 (183367 to 229333)	115376 (102794 to 129566)	89316 (79942 to 100057)	211.5 (201.5 to 221.7)	223.9 (211.6 to 236.8)	196.8 (183.4 to 212.5)
		Age-standardized (rate per 100,000)	5098.4 (4568.4 to 5709.4)	5736 (5126.7 to 6484.4)	4485.9 (3994.6 to 4996.2)	5450.4 (4916.5 to 6066.4)	6150.6 (5509.2 to 6888.9)	4747.4 (4236 to 5252.9)	6.9 (3.9 to 10)	7.2 (3.3 to 10.9)	5.8 (1.4 to 10.6)
	YLDs	All ages (number)	2266 (1151 to 4444)	1235 (627 to 2427)	1031 (516 to 2047)	7099 (3587 to 14055)	4027 (2046 to 7905)	3071 (1530 to 6032)	213.3 (201.7 to 225.5)	226 (210.8 to 240.5)	198 (181.8 to 215.4)
		Age-standardized (rate per 100,000)	175.4 (88.6 to 344.8)	198.8 (100.8 to 389.2)	152.9 (77.3 to 305.1)	189.4 (95.3 to 369.8)	215.3 (109.7 to 422.5)	163.2 (82.1 to 320.7)	8 (4.2 to 11.5)	8.3 (3.6 to 13)	6.8 (1.6 to 12.2)

Province	Measure	Age (metric)	Year						% Change (1990 to 2019)		
			1990			2019					
			Both	Female	Male	Both	Female	Male	Both	Female	Male
Kohgiluyeh and Boyer-Ahmad	Incidence	All ages (number)	831 (730 to 944)	425 (373 to 485)	406 (352 to 461)	2781 (2455 to 3142)	1541 (1350 to 1753)	1240 (1102 to 1400)	234.5 (219.6 to 249.7)	262.2 (243.8 to 279.9)	205.5 (187.5 to 224.1)
		Age-standardized (rate per 100,000)	400.3 (356.4 to 451.3)	450.9 (398.6 to 513.7)	358 (317.8 to 402)	435.6 (386.6 to 491.8)	490.2 (432.9 to 556.4)	382.9 (340.8 to 430.6)	8.8 (5.9 to 12.1)	8.7 (4.2 to 13.2)	7 (2.7 to 11.5)
	Prevalence	All ages (number)	8832 (7843 to 9919)	4551 (4040 to 5164)	4281 (3752 to 4807)	28586 (25611 to 32059)	15686 (13883 to 17724)	12900 (11470 to 14441)	223.7 (213.2 to 235.1)	244.7 (229.7 to 260.3)	201.3 (186.8 to 217.3)
		Age-standardized (rate per 100,000)	5035.4 (4512.2 to 5622.5)	5697.6 (5091.9 to 6430)	4449.2 (3974.7 to 4947.3)	5417 (4879.6 to 6025.7)	6144.2 (5487.9 to 6925.8)	4742.5 (4236.7 to 5266.6)	7.6 (4.9 to 10.6)	7.8 (3.9 to 12.1)	6.6 (2.3 to 10.7)
	YLDs	All ages (number)	305 (153 to 599)	158 (80 to 314)	147 (73 to 293)	996 (498 to 1952)	550 (276 to 1075)	446 (223 to 888)	226 (214 to 239.7)	247.4 (230.3 to 266.6)	202.9 (186.3 to 221.2)
		Age-standardized (rate per 100,000)	173.5 (87.4 to 340.7)	197.8 (100.3 to 393.7)	151.9 (76.4 to 305.8)	189.4 (95 to 369.8)	216.7 (109.1 to 423.7)	164.1 (81.5 to 328)	9.1 (6 to 13)	9.5 (4.7 to 14.8)	8 (3 to 13.1)
Kurdistan	Incidence	All ages (number)	2611 (2302 to 2973)	1379 (1208 to 1582)	1231 (1081 to 1400)	7343 (6526 to 8344)	4130 (3652 to 4716)	3213 (2850 to 3631)	181.3 (171 to 192.5)	199.4 (186.2 to 212.8)	160.9 (147.4 to 176.3)
		Age-standardized (rate per 100,000)	399.5 (354.1 to 452)	449.6 (396.4 to 512.8)	355 (314.4 to 401)	432.5 (385 to 488.7)	485.8 (429.7 to 553.3)	379.1 (337.6 to 426.4)	8.3 (4.8 to 11.8)	8.1 (3.6 to 12.7)	6.8 (2.2 to 11.8)
	Prevalence	All ages (number)	28720 (25451 to 32345)	14848 (13077 to 16976)	13871 (12322 to 15511)	80775 (72298 to 90089)	45725 (40644 to 51554)	35050 (31308 to 38984)	181.3 (171.8 to 190.9)	208 (195.1 to 219.9)	152.7 (140.8 to 164.7)
		Age-standardized (rate per 100,000)	4986.4 (4454.9 to 5580.1)	5678.1 (5042.2 to 6442.9)	4408.7 (3934.9 to 4891)	5398.8 (4848.2 to 6021.3)	6098.9 (5449.1 to 6851)	4694.7 (4200.2 to 5202.2)	8.3 (5 to 11.7)	7.4 (3.6 to 11.2)	6.5 (1.8 to 11.3)
	YLDs	All ages (number)	994 (501 to 1943)	517 (258 to 1007)	477 (239 to 936)	2816 (1408 to 5656)	1606 (810 to 3194)	1210 (599 to 2447)	183.2 (172.4 to 194.8)	210.7 (194.7 to 225)	153.5 (139 to 167.5)
		Age-standardized (rate per 100,000)	172.5 (86.6 to 338)	198.1 (100.3 to 386.6)	151.2 (75.6 to 296.1)	188.6 (95.5 to 378.1)	214.9 (108.8 to 427.3)	162.2 (80.8 to 325.2)	9.4 (5.5 to 13.3)	8.5 (3.4 to 13.1)	7.2 (1.5 to 12.6)

Province	Measure	Age (metric)	Year						% Change (1990 to 2019)		
			1990			2019					
			Both	Female	Male	Both	Female	Male	Both	Female	Male
Lorestan	Incidence	All ages (number)	2999 (2639 to 3397)	1561 (1371 to 1784)	1437 (1265 to 1625)	7835 (6938 to 8817)	4456 (3945 to 5049)	3380 (3011 to 3822)	161.3 (151.4 to 172)	185.4 (173.6 to 197.7)	135.2 (121.6 to 149.5)
		Age-standardized (rate per 100,000)	403.6 (359 to 455.3)	453.8 (402.4 to 517.4)	360 (320.1 to 404.6)	437.2 (389.4 to 492.9)	489.9 (433.7 to 554.5)	383.1 (341.4 to 431)	8.3 (5.2 to 11.6)	8 (3.6 to 12.4)	6.4 (1.9 to 11.5)
	Prevalence	All ages (number)	32874 (29247 to 37047)	16822 (14805 to 19021)	16052 (14281 to 17997)	85971 (77348 to 95852)	49479 (44113 to 55844)	36492 (32639 to 40642)	161.5 (153.9 to 170.4)	194.1 (182.6 to 205.9)	127.3 (116.9 to 138.9)
		Age-standardized (rate per 100,000)	5051.3 (4517.8 to 5635.8)	5729 (5100 to 6458.6)	4477.9 (4011 to 4983.7)	5463.1 (4907.5 to 6064.7)	6147.9 (5507.4 to 6907.6)	4743.9 (4255 to 5266.5)	8.2 (5.4 to 11.2)	7.3 (3.4 to 11.4)	5.9 (1.8 to 10.7)
	YLDs	All ages (number)	1137 (571 to 2225)	585 (293 to 1153)	552 (277 to 1076)	3001 (1494 to 5967)	1740 (866 to 3432)	1261 (632 to 2547)	163.9 (154.3 to 174.2)	197.3 (184.3 to 211.6)	128.5 (116.4 to 142.6)
		Age-standardized (rate per 100,000)	174.3 (87.7 to 338.3)	199.3 (100.3 to 390.9)	153.2 (76.7 to 300.3)	191.2 (95.8 to 379.2)	216.8 (108.6 to 432.3)	164.1 (82.4 to 331.2)	9.7 (6.3 to 13.4)	8.8 (4.1 to 13.7)	7.1 (1.9 to 12.9)
Markazi	Incidence	All ages (number)	2894 (2550 to 3284)	1606 (1405 to 1835)	1289 (1137 to 1458)	7173 (6385 to 8048)	4012 (3551 to 4541)	3161 (2812 to 3538)	147.8 (139 to 156.9)	149.9 (138.8 to 161.1)	145.3 (132.6 to 158.8)
		Age-standardized (rate per 100,000)	408.9 (364 to 461.3)	455.8 (404 to 518.3)	361.7 (321 to 407.4)	436.2 (388.6 to 490.9)	490.4 (434.8 to 555.7)	382.7 (339.9 to 428.3)	6.7 (3.9 to 9.7)	7.6 (3.3 to 12.2)	5.8 (1.4 to 10.2)
	Prevalence	All ages (number)	33024 (29289 to 37102)	18124 (16081 to 20612)	14900 (13165 to 16737)	83282 (75135 to 92610)	47165 (42193 to 53035)	36117 (32292 to 40065)	152.2 (144.9 to 159.8)	160.2 (150.2 to 170.7)	142.4 (131.7 to 153.9)
		Age-standardized (rate per 100,000)	5118.9 (4584.9 to 5711.6)	5756.3 (5141.9 to 6482.9)	4498.4 (4011.4 to 5018.9)	5450.5 (4902.9 to 6053.1)	6147.4 (5497.7 to 6904.5)	4741.4 (4242 to 5258.1)	6.5 (3.9 to 9.4)	6.8 (3.3 to 10.8)	5.4 (1.3 to 9.7)
	YLDs	All ages (number)	1142 (577 to 2237)	631 (319 to 1224)	511 (257 to 1002)	2901 (1468 to 5723)	1655 (838 to 3276)	1245 (624 to 2454)	154.1 (145.2 to 163.4)	162.5 (150.6 to 175.5)	143.7 (131 to 157.9)
		Age-standardized (rate per 100,000)	176.3 (89.1 to 344)	199.9 (101.9 to 388.3)	153.5 (77.2 to 299.3)	190.2 (96.4 to 375.8)	216.2 (109.4 to 427.8)	163.7 (81.9 to 324.1)	7.9 (4.8 to 11.5)	8.2 (3.8 to 13.2)	6.7 (1.7 to 12.1)

Province	Measure	Age (metric)	Year						% Change (1990 to 2019)		
			1990			2019					
			Both	Female	Male	Both	Female	Male	Both	Female	Male
Mazandaran	Incidence	All ages (number)	6005 (5316 to 6785)	3300 (2907 to 3750)	2705 (2391 to 3052)	18650 (16428 to 21066)	10521 (9214 to 11965)	8129 (7182 to 9202)	210.6 (201.3 to 220.8)	218.9 (205.7 to 233.3)	200.5 (186.2 to 215.2)
		Age-standardized (rate per 100,000)	412.2 (367.5 to 463.8)	459.7 (407.4 to 521.1)	365.9 (325.9 to 409.9)	443.3 (393.7 to 500.2)	496.4 (437.2 to 563.2)	389.6 (345.6 to 437.9)	7.5 (4.3 to 10.8)	8 (3.8 to 12.6)	6.5 (1.6 to 11.2)
	Prevalence	All ages (number)	65398 (58260 to 73507)	35908 (31761 to 40541)	29489 (26140 to 33056)	213069 (190347 to 238784)	121487 (107943 to 136864)	91582 (81907 to 102239)	225.8 (216.9 to 235.6)	238.3 (226.2 to 251.7)	210.6 (196.5 to 225.6)
		Age-standardized (rate per 100,000)	5179.1 (4654 to 5782.3)	5802.3 (5167 to 6502.9)	4555.2 (4066 to 5078.6)	5536.6 (4974.1 to 6165.6)	6222 (5555.1 to 6977.9)	4830.2 (4333.6 to 5358.8)	6.9 (4.2 to 9.8)	7.2 (3.6 to 11.1)	6 (1.7 to 10.7)
	YLDs	All ages (number)	2253 (1137 to 4411)	1238 (625 to 2428)	1015 (510 to 2058)	7391 (3737 to 14446)	4224 (2137 to 8279)	3167 (1593 to 6314)	228 (217.1 to 240.4)	241.2 (227 to 258.2)	212 (195.3 to 229.6)
		Age-standardized (rate per 100,000)	177.9 (90.1 to 348.9)	199.8 (101.3 to 390.2)	156 (78.4 to 317.1)	192.2 (96.8 to 378)	216.6 (110.5 to 426)	167 (84 to 330.9)	8 (4.8 to 11.7)	8.4 (4.1 to 13.4)	7.1 (1.7 to 12.4)
North Khorasan	Incidence	All ages (number)	1371 (1211 to 1548)	740 (650 to 838)	632 (559 to 713)	3658 (3248 to 4139)	2082 (1828 to 2373)	1576 (1400 to 1778)	166.8 (156.7 to 177.3)	181.5 (169.3 to 195.6)	149.5 (135.7 to 164)
		Age-standardized (rate per 100,000)	401.7 (358.8 to 451.5)	450.5 (399.7 to 509.5)	356.1 (319.9 to 400)	436.7 (388.7 to 493.5)	489.3 (432.7 to 557.4)	382.6 (340.4 to 431.4)	8.7 (5.5 to 12.6)	8.6 (3.9 to 13.6)	7.4 (2.5 to 12.7)
	Prevalence	All ages (number)	14753 (13104 to 16570)	7814 (6839 to 8770)	6940 (6166 to 7792)	40676 (36556 to 45573)	23176 (20611 to 26230)	17500 (15635 to 19627)	175.7 (167.5 to 184.4)	196.6 (183.9 to 209.8)	152.2 (139.3 to 164.5)
		Age-standardized (rate per 100,000)	5025.9 (4510.7 to 5601.8)	5692.8 (5047.1 to 6360.3)	4424 (3966.9 to 4906.6)	5444.3 (4899.4 to 6072)	6136.5 (5492.7 to 6950.3)	4737.9 (4250.3 to 5288.8)	8.3 (5.3 to 11.6)	7.8 (3.5 to 11.9)	7.1 (2.4 to 12.3)
	YLDs	All ages (number)	509 (256 to 993)	271 (136 to 539)	238 (120 to 468)	1412 (714 to 2810)	810 (412 to 1591)	602 (297 to 1196)	177.6 (167.7 to 188.4)	199.2 (184.6 to 214.2)	153 (138.8 to 167.5)
		Age-standardized (rate per 100,000)	173 (87.7 to 340.1)	197.4 (99.7 to 394.2)	151.1 (76.2 to 298.4)	189.3 (95.5 to 374.7)	214.9 (109.3 to 420.9)	163.2 (81.1 to 324.7)	9.4 (5.8 to 13.2)	8.9 (4.3 to 13.9)	8 (2.5 to 14.1)

Province	Measure	Age (metric)	Year						% Change (1990 to 2019)		
			1990			2019					
			Both	Female	Male	Both	Female	Male	Both	Female	Male
Qazvin	Incidence	All ages (number)	2018 (1777 to 2287)	1091 (957 to 1251)	927 (819 to 1051)	6109 (5393 to 6895)	3400 (2982 to 3872)	2709 (2395 to 3074)	202.7 (191.8 to 215.3)	211.5 (197.5 to 227.3)	192.4 (177.2 to 208.7)
		Age-standardized (rate per 100,000)	408.3 (362.6 to 460.9)	456.7 (402.8 to 522.4)	362.3 (322.7 to 409.3)	436.7 (387.4 to 492.6)	491 (434.8 to 555.5)	383.7 (340.2 to 434.5)	7 (3.9 to 10.3)	7.5 (3 to 12.4)	5.9 (1.5 to 10.8)
	Prevalence	All ages (number)	22668 (20174 to 25526)	12215 (10771 to 13883)	10453 (9256 to 11838)	65855 (58873 to 73768)	37469 (33285 to 42292)	28386 (25365 to 31878)	190.5 (181.3 to 200.3)	206.7 (194.4 to 221)	171.6 (158.6 to 184.4)
		Age-standardized (rate per 100,000)	5127.5 (4589.5 to 5744)	5765 (5122.6 to 6514)	4513.5 (4039.3 to 5062.8)	5469 (4927.9 to 6096)	6159.6 (5503.5 to 6915.3)	4755.3 (4263.9 to 5297.3)	6.7 (3.7 to 9.8)	6.8 (2.9 to 11.5)	5.4 (1 to 9.8)
	YLDs	All ages (number)	786 (392 to 1560)	426 (214 to 844)	360 (179 to 716)	2297 (1151 to 4511)	1315 (661 to 2580)	981 (486 to 1990)	192.4 (182.2 to 203.1)	209.1 (194.6 to 225.1)	172.6 (158.7 to 187.1)
		Age-standardized (rate per 100,000)	177.4 (89.6 to 351.8)	200.9 (101.4 to 397.9)	154.9 (77.6 to 309.3)	191.2 (95.6 to 375.5)	217 (109.2 to 425.9)	164.5 (81.5 to 329.4)	7.8 (4.4 to 11.3)	8 (3.4 to 13.3)	6.2 (1.2 to 11.2)
Qom	Incidence	All ages (number)	1488 (1311 to 1679)	797 (699 to 907)	691 (611 to 777)	5712 (5057 to 6414)	3130 (2753 to 3553)	2582 (2283 to 2901)	283.9 (270 to 297.5)	292.7 (276.2 to 310.1)	273.7 (254.9 to 293.3)
		Age-standardized (rate per 100,000)	406.3 (360 to 458.5)	455.2 (402.5 to 517.4)	361.4 (320.5 to 405.2)	434.8 (386.9 to 489.5)	490.4 (434.5 to 556.6)	382.7 (340.5 to 428.9)	7 (3.8 to 10.2)	7.8 (3.5 to 12.2)	5.9 (1.1 to 10.6)
	Prevalence	All ages (number)	16273 (14496 to 18207)	8795 (7795 to 9929)	7478 (6616 to 8358)	60569 (54111 to 67947)	33175 (29513 to 37515)	27395 (24467 to 30497)	272.2 (260.2 to 283.8)	277.2 (263.7 to 292.3)	266.4 (249.9 to 283.7)
		Age-standardized (rate per 100,000)	5105.4 (4589 to 5658.8)	5748.5 (5135.1 to 6466.3)	4495.1 (4013.4 to 4978)	5422.7 (4885.3 to 6021.4)	6152.9 (5504.2 to 6926)	4744.1 (4257.8 to 5244.4)	6.2 (3.3 to 9.3)	7 (3.6 to 11.1)	5.5 (1.4 to 10)
	YLDs	All ages (number)	561 (283 to 1101)	305 (154 to 596)	256 (127 to 505)	2105 (1056 to 4151)	1160 (574 to 2284)	945 (470 to 1870)	275.5 (261.4 to 289.1)	280.4 (263 to 299.1)	269.6 (248.3 to 289.4)
		Age-standardized (rate per 100,000)	175.3 (88.6 to 341.6)	198.9 (100.4 to 389.7)	152.8 (76.6 to 300.7)	189 (94.8 to 371.1)	216 (107.6 to 423.2)	163.8 (82 to 322.5)	7.8 (4.3 to 11.5)	8.6 (4 to 13.7)	7.2 (1.6 to 12.3)

Province	Measure	Age (metric)	Year						% Change (1990 to 2019)		
			1990			2019					
			Both	Female	Male	Both	Female	Male	Both	Female	Male
Semnan	Incidence	All ages (number)	1231 (1089 to 1395)	679 (597 to 773)	552 (487 to 627)	3415 (3034 to 3860)	1909 (1693 to 2178)	1506 (1333 to 1693)	177.4 (167.2 to 187.5)	181.1 (168.3 to 194.6)	172.8 (158.9 to 186.6)
		Age-standardized (rate per 100,000)	409.7 (364.6 to 463.2)	456.5 (404.6 to 519.9)	363.1 (322 to 408.8)	438.6 (389.8 to 494.9)	493.2 (437.1 to 564.4)	385 (341.8 to 430.6)	7 (3.9 to 9.8)	8 (3.8 to 12.5)	6 (0.9 to 10.3)
	Prevalence	All ages (number)	14345 (12757 to 16028)	7962 (7051 to 8992)	6382 (5631 to 7148)	38458 (34573 to 43001)	21886 (19548 to 24751)	16572 (14787 to 18445)	168.1 (160 to 175.7)	174.9 (164 to 185.9)	159.7 (148.1 to 170.5)
		Age-standardized (rate per 100,000)	5152.2 (4604.8 to 5748.8)	5766.4 (5123.5 to 6491.4)	4515.3 (4028.2 to 5025.2)	5484.1 (4934.6 to 6106.4)	6180 (5531.2 to 6970.1)	4770.1 (4261.6 to 5287.7)	6.4 (3.6 to 9.1)	7.2 (3.3 to 11.2)	5.6 (1.3 to 9.7)
	YLDs	All ages (number)	497 (250 to 972)	278 (141 to 550)	219 (109 to 432)	1341 (681 to 2651)	768 (390 to 1518)	573 (284 to 1143)	170 (159.8 to 179.1)	176.7 (164 to 190.4)	161.5 (147.9 to 174.6)
		Age-standardized (rate per 100,000)	177.5 (89.6 to 346.8)	200.4 (101.8 to 396.6)	153.8 (76.8 to 305.4)	191.4 (96.9 to 379.8)	217.3 (110.2 to 429.2)	164.8 (82 to 329.7)	7.8 (4.2 to 11.2)	8.5 (3.8 to 13.7)	7.1 (1.9 to 12.2)
Sistan and Baluchistan	Incidence	All ages (number)	2604 (2303 to 2949)	1321 (1161 to 1503)	1283 (1128 to 1456)	7493 (6633 to 8439)	4233 (3729 to 4800)	3260 (2863 to 3676)	187.7 (175.4 to 199.2)	220.4 (206.5 to 235.3)	154.2 (139.4 to 169)
		Age-standardized (rate per 100,000)	392.7 (349.9 to 442.8)	444.3 (395.1 to 504.6)	350.5 (310.1 to 394.1)	425.9 (377.8 to 479.3)	478.6 (423.4 to 542.4)	373 (330.5 to 418.3)	8.5 (5.1 to 11.5)	7.7 (4 to 12.3)	6.4 (1.8 to 11.1)
	Prevalence	All ages (number)	28148 (25008 to 31686)	14024 (12409 to 15958)	14124 (12514 to 15882)	76103 (67926 to 85125)	43047 (38156 to 48575)	33056 (29296 to 36917)	170.4 (161 to 178.4)	207 (195.3 to 220.4)	134 (122.9 to 144.9)
		Age-standardized (rate per 100,000)	4914.1 (4412 to 5487.7)	5620.9 (5018.8 to 6355.2)	4352 (3891.8 to 4843.7)	5312.9 (4762.9 to 5921.9)	6014 (5389.2 to 6735.2)	4615.5 (4110.3 to 5131.6)	8.1 (5.1 to 11)	7 (3.5 to 11.2)	6.1 (1.5 to 10.3)
	YLDs	All ages (number)	972 (493 to 1921)	487 (247 to 965)	485 (242 to 952)	2641 (1333 to 5199)	1502 (758 to 2939)	1139 (565 to 2256)	171.7 (161.4 to 181.7)	208.7 (195.2 to 223.9)	134.6 (122 to 147.1)
		Age-standardized (rate per 100,000)	169.4 (85.9 to 334.1)	195.2 (99.5 to 384.8)	149 (74.9 to 291.8)	185 (93.6 to 365.4)	211.1 (106.9 to 411.6)	159.1 (79.3 to 323)	9.2 (5.7 to 12.7)	8.1 (4.1 to 13.1)	6.8 (1.7 to 11.8)

Province	Measure	Age (metric)	Year						% Change (1990 to 2019)		
			1990			2019					
			Both	Female	Male	Both	Female	Male	Both	Female	Male
South Khorasan	Incidence	All ages (number)	1621 (1424 to 1832)	864 (759 to 981)	757 (663 to 855)	3363 (2975 to 3779)	1935 (1709 to 2203)	1428 (1271 to 1597)	107.4 (99.5 to 116.3)	123.8 (113.8 to 134.5)	88.7 (78.4 to 100.1)
		Age-standardized (rate per 100,000)	401.3 (356.2 to 452)	450 (399.2 to 511.5)	356.5 (316 to 400.8)	435.5 (384.4 to 490.7)	487.4 (429.8 to 557.3)	380.5 (336.7 to 427)	8.5 (5.2 to 12.1)	8.3 (4.2 to 13)	6.7 (2.3 to 11.6)
	Prevalence	All ages (number)	18729 (16672 to 21096)	9920 (8754 to 11306)	8809 (7781 to 9862)	38979 (35020 to 43389)	22625 (20247 to 25304)	16355 (14659 to 18235)	108.1 (101.8 to 114.9)	128.1 (118.7 to 137.9)	85.7 (77.5 to 93.9)
		Age-standardized (rate per 100,000)	5024.3 (4511.3 to 5611.7)	5687.7 (5077.8 to 6418.1)	4425.7 (3940.9 to 4916.6)	5440.7 (4884.8 to 6044.5)	6115.9 (5475.8 to 6844.9)	4716 (4228.4 to 5253.2)	8.3 (5.3 to 11.3)	7.5 (3.6 to 11.7)	6.6 (2.3 to 10.6)
	YLDs	All ages (number)	647 (325 to 1272)	344 (172 to 672)	302 (151 to 605)	1350 (676 to 2661)	788 (396 to 1558)	562 (282 to 1106)	108.9 (102.2 to 117.3)	129 (118.5 to 140.6)	85.9 (76.8 to 95.9)
		Age-standardized (rate per 100,000)	173.1 (86.7 to 340.9)	197.2 (98.8 to 386.5)	151.3 (75.7 to 301)	188.8 (95 to 373.5)	213.6 (107.9 to 422.4)	162.1 (81.5 to 317.8)	9.1 (5.6 to 13)	8.3 (3.7 to 13.2)	7.1 (2.5 to 12.2)
Tehran	Incidence	All ages (number)	23306 (20608 to 26401)	12771 (11198 to 14586)	10535 (9294 to 11847)	78418 (69496 to 88628)	44888 (39467 to 51313)	33530 (29697 to 37922)	236.5 (226.8 to 247.7)	251.5 (238.5 to 265.6)	218.3 (203.9 to 234.9)
		Age-standardized (rate per 100,000)	453.3 (402.4 to 511.2)	519.9 (455.3 to 593.1)	393 (349.8 to 438.8)	480.7 (426 to 543.7)	552 (487.2 to 630.5)	410.5 (364 to 462.6)	6 (3.1 to 9.4)	6.2 (2.3 to 10.4)	4.5 (0.3 to 9.3)
	Prevalence	All ages (number)	252937 (225925 to 285667)	141188 (124311 to 160842)	111750 (99849 to 125363)	897019 (803676 to 1006313)	515966 (458664 to 584470)	381053 (341726 to 425404)	254.6 (245.5 to 266.9)	265.4 (252.5 to 279.2)	241 (226.9 to 257.7)
		Age-standardized (rate per 100,000)	5865.5 (5269.7 to 6575.1)	6765.8 (5986.1 to 7670.7)	4994.3 (4486.9 to 5545.3)	6144.9 (5526.5 to 6862.1)	7115.9 (6340.3 to 8022.5)	5188.8 (4666.5 to 5758.9)	4.8 (2.2 to 8)	5.2 (1.9 to 8.8)	3.9 (-0.1 to 8.3)
	YLDs	All ages (number)	8864 (4513 to 17500)	4990 (2551 to 10032)	3875 (1961 to 7709)	31592 (16038 to 62282)	18353 (9327 to 36640)	13239 (6652 to 25903)	256.4 (244.7 to 270.7)	267.8 (252.4 to 284.1)	241.7 (225.5 to 261.3)
		Age-standardized (rate per 100,000)	206 (105.4 to 404.8)	239.9 (122.8 to 477.7)	173 (87.7 to 340.9)	217 (110.8 to 427.8)	254 (128.7 to 504.9)	180.5 (90.4 to 354.3)	5.3 (2.2 to 9.1)	5.9 (1.6 to 10.3)	4.3 (-0.2 to 9.6)

Province	Measure	Age (metric)	Year						% Change (1990 to 2019)		
			1990			2019					
			Both	Female	Male	Both	Female	Male	Both	Female	Male
West Azarbayejan	Incidence	All ages (number)	4993 (4385 to 5656)	2688 (2343 to 3080)	2306 (2036 to 2610)	14510 (12798 to 16431)	8184 (7149 to 9345)	6326 (5602 to 7136)	190.6 (180.3 to 201.2)	204.5 (189.7 to 218.7)	174.4 (159 to 188.4)
		Age-standardized (rate per 100,000)	402 (357.9 to 453.8)	450.3 (398.7 to 513.1)	357 (318.2 to 400)	431.4 (383.5 to 487.6)	485.7 (426.7 to 553.1)	377.3 (336.5 to 422.4)	7.3 (4.3 to 10.7)	7.9 (3.6 to 12.6)	5.7 (0.8 to 10.6)
	Prevalence	All ages (number)	53652 (47777 to 60832)	28561 (25223 to 32591)	25091 (22201 to 28336)	157156 (140982 to 175677)	90144 (79847 to 101549)	67011 (59785 to 75047)	192.9 (183.3 to 202.4)	215.6 (203.2 to 228.8)	167.1 (154.1 to 180.3)
		Age-standardized (rate per 100,000)	5030.2 (4519.5 to 5620.2)	5689.2 (5068.1 to 6426.1)	4430.2 (3973.2 to 4944.7)	5399.2 (4852.9 to 6004.9)	6093.2 (5415.4 to 6874.8)	4676.3 (4199.5 to 5190.8)	7.3 (4.4 to 10.5)	7.1 (3.3 to 10.9)	5.6 (1 to 10.1)
	YLD s	All ages (number)	1857 (929 to 3671)	992 (499 to 1973)	864 (429 to 1707)	5465 (2756 to 10626)	3152 (1575 to 6179)	2313 (1182 to 4609)	194.3 (183.2 to 205.8)	217.6 (202.2 to 233.2)	167.7 (152.4 to 183)
		Age-standardized (rate per 100,000)	173.7 (87.4 to 343)	197.7 (99.6 to 389.5)	151.8 (75.6 to 300.9)	188 (95.2 to 367.8)	213.5 (107.8 to 422.5)	161.3 (81.7 to 323.5)	8.3 (4.8 to 12.2)	8 (3.5 to 13)	6.3 (0.8 to 11.9)
Yazd	Incidence	All ages (number)	1623 (1429 to 1842)	891 (788 to 1017)	733 (643 to 827)	4897 (4339 to 5527)	2656 (2339 to 3024)	2241 (1978 to 2520)	201.6 (189.4 to 213.8)	198.2 (184.3 to 212.6)	205.9 (188.6 to 222.2)
		Age-standardized (rate per 100,000)	411.2 (365.5 to 464.1)	458.6 (407.1 to 521.2)	364.8 (321.5 to 409.8)	437.6 (387.4 to 494.7)	493.5 (435.2 to 560.2)	386.2 (341.5 to 433.6)	6.4 (3.3 to 9.5)	7.6 (3.4 to 12.2)	5.9 (1 to 10)
	Prevalence	All ages (number)	18696 (16601 to 20905)	10496 (9271 to 11885)	8200 (7264 to 9223)	53824 (48244 to 60153)	29647 (26474 to 33499)	24177 (21556 to 27034)	187.9 (179 to 197.7)	182.5 (170.8 to 193.8)	194.8 (180.4 to 208.7)
		Age-standardized (rate per 100,000)	5184.7 (4641.5 to 5757.7)	5790 (5155.5 to 6525.4)	4537.9 (4062.6 to 5053.6)	5476.4 (4910.3 to 6095.5)	6192.1 (5541.5 to 7003.1)	4790.6 (4273.6 to 5328.9)	5.6 (2.7 to 8.5)	6.9 (3.1 to 11)	5.6 (1 to 9.7)
	YLD s	All ages (number)	647 (327 to 1294)	366 (185 to 708)	281 (141 to 560)	1872 (945 to 3671)	1039 (525 to 2060)	833 (418 to 1652)	189 (178.6 to 199.8)	183.8 (170.1 to 197.3)	195.9 (179.5 to 212.6)
		Age-standardized (rate per 100,000)	178.9 (90.7 to 357)	201.4 (101.9 to 390.3)	154.7 (77.7 to 310.2)	190.8 (95.9 to 375.9)	217.6 (110.1 to 427.3)	164.9 (82.9 to 331.9)	6.7 (3.2 to 10)	8.1 (3.3 to 13.1)	6.6 (1.5 to 11.8)

Province	Measure	Age (metric)	Year						% Change (1990 to 2019)		
			1990			2019					
			Both	Female	Male	Both	Female	Male	Both	Female	Male
Zanjan	Incidence	All ages (number)	1936 (1696 to 2197)	1046 (917 to 1195)	890 (783 to 1008)	4947 (4389 to 5591)	2801 (2480 to 3184)	2145 (1897 to 2419)	155.5 (145.4 to 166.3)	167.8 (156.1 to 180.6)	141 (126.7 to 156.5)
		Age-standardized (rate per 100,000)	404.8 (360 to 457.7)	453.2 (400.6 to 515.1)	358.3 (320.2 to 403.1)	434.8 (387.7 to 490.1)	488.2 (432 to 557)	380.7 (338.5 to 427.6)	7.4 (4.4 to 10.6)	7.7 (3.6 to 12.8)	6.2 (1.7 to 11.1)
	Prevalence	All ages (number)	21766 (19259 to 24502)	11598 (10248 to 13111)	10168 (9014 to 11409)	55616 (49976 to 62106)	32121 (28633 to 36158)	23495 (21064 to 26082)	155.5 (147.4 to 164)	177 (166.3 to 188.9)	131.1 (119.4 to 142.3)
		Age-standardized (rate per 100,000)	5072.4 (4542.7 to 5654.4)	5721.7 (5094 to 6439)	4457 (3983.9 to 4959.9)	5440.7 (4892.4 to 6052.3)	6123.8 (5472 to 6884.2)	4715.7 (4226.2 to 5215.1)	7.3 (4.5 to 10.1)	7 (3.4 to 11.2)	5.8 (1.5 to 10.1)
	YLDs	All ages (number)	752 (379 to 1469)	403 (202 to 795)	349 (174 to 686)	1936 (975 to 3827)	1126 (569 to 2233)	810 (404 to 1610)	157.4 (148.2 to 167.7)	179.4 (166.4 to 192.8)	131.9 (119.4 to 146.1)
		Age-standardized (rate per 100,000)	175 (88.1 to 341.8)	198.9 (99.5 to 391.3)	152.4 (76.1 to 299.7)	189.8 (95.7 to 373.7)	215.2 (109.2 to 422.3)	162.7 (81.3 to 322.4)	8.5 (5.1 to 11.9)	8.2 (3.8 to 13)	6.8 (2 to 12.1)

Data in parenthesis are 95% uncertainty intervals, YLDs: Years Lived with Disability
