# Supplementary material for: National and subnational burden and attributable risk factors of osteoarthritis, rheumatoid arthritis, and low back pain in Iran: 1990–2019 findings of the Global Burden of Disease (GBD) Study
Source: PLoS One. 2026 Jul 6;21(7):e0344038. doi: 10.1371/journal.pone.0344038 (PMC13336167; doi:10.1371/journal.pone.0344038)
Supplement: S4 Table — (PDF) [file pone.0344038.s004.pdf]

S4 Table. All ages number and age-standardized rate of incidence, prevalence, and YLDs of LBP in 1990 and 2019

Province	Measure	Age-standardized rate (per 100,000)						% Change (1990 to 2019)		
		1990			2019					
		Both	Female	Male	Both	Female	Male	Both	Female	Male
Alborz	Incidence	3744.9 (3316.1 to 4247.8)	3544.6 (3122.8 to 4031.3)	3918.1 (3466.6 to 4469.3)	3407.3 (3008.1 to 3849.3)	3359.2 (2975 to 3804)	3460.7 (3044.2 to 3943.4)	-9 (-11.7 to -6.3)	-5.2 (-9.1 to -1.3)	-11.7 (-15.2 to -7.8)
	Prevalence	9010.2 (7940.3 to 10207)	8640.7 (7596.4 to 9852.4)	9326.3 (8169.9 to 10604.2)	8080.7 (7108.7 to 9140.5)	8103.3 (7117.3 to 9237.8)	8076.9 (7092.2 to 9130.3)	-10.3 (-13.8 to -6.6)	-6.2 (-11.5 to -0.6)	-13.4 (-17.7 to -8.6)
	YLDs	1013.8 (714.2 to 1368.6)	960.7 (673.9 to 1294.2)	1059.7 (746.3 to 1433.5)	907.9 (637.4 to 1212.6)	900.6 (632.3 to 1201.5)	916.7 (644.3 to 1230.4)	-10.4 (-14.4 to -6.3)	-6.3 (-12 to -0.2)	-13.5 (-18.3 to -8)
Ardebil	Incidence	3794 (3338.4 to 4302.1)	3589.9 (3155.4 to 4073.6)	3984.7 (3507.5 to 4528.5)	3469.6 (3065.5 to 3927.8)	3411.4 (3006.6 to 3862.6)	3523.5 (3106.6 to 4004.4)	-8.5 (-11 to -5.8)	-5 (-8.7 to -1)	-11.6 (-15.1 to -7.8)
	Prevalence	9220.4 (8128.9 to 10399.8)	8869.2 (7791.7 to 10063.3)	9547.8 (8387.5 to 10813.4)	8292.5 (7255.4 to 9349.8)	8298.2 (7278.4 to 9370.6)	8279.4 (7207.5 to 9367.4)	-10.1 (-13.4 to -6.2)	-6.4 (-11.4 to -1.2)	-13.3 (-17.9 to -7.9)
	YLDs	1034.2 (728.6 to 1382.3)	983.7 (693.1 to 1320.8)	1082.1 (760.3 to 1445.6)	931.4 (658.3 to 1241.4)	922.4 (648.2 to 1232.3)	939.2 (656.6 to 1261.6)	-9.9 (-13.9 to -5.9)	-6.2 (-11.7 to -0.4)	-13.2 (-18.5 to -7.5)
Bushehr	Incidence	3710.2 (3278.9 to 4203.8)	3515.5 (3103.5 to 3981.6)	3894.3 (3420.7 to 4445.8)	3391.9 (3002.6 to 3853)	3334 (2943.3 to 3794.2)	3438.4 (3031.2 to 3910)	-8.6 (-11.2 to -5.8)	-5.2 (-9.3 to -1.3)	-11.7 (-15.3 to -8)
	Prevalence	8914.1 (7845.7 to 10099.6)	8551.7 (7482 to 9678.5)	9251.3 (8108.2 to 10484.4)	8016.7 (7025 to 9042.7)	8029.2 (7077.3 to 9065.2)	7990.8 (6984.4 to 9067.7)	-10.1 (-13.5 to -6.2)	-6.1 (-11.2 to -0.4)	-13.6 (-18.6 to -8.4)
	YLDs	1001.7 (705.7 to 1349)	950.6 (660.8 to 1279.9)	1050 (743.3 to 1426.1)	901.1 (634.9 to 1202.4)	892.3 (627.1 to 1193.3)	907.3 (634.6 to 1225.2)	-10 (-13.6 to -5.9)	-6.1 (-11.9 to 0.2)	-13.6 (-18.7 to -8.2)
Chahar Mahaal and Bakhtiari	Incidence	3749.5 (3308 to 4243.2)	3549.8 (3135.5 to 4005.6)	3928.7 (3446.8 to 4454.1)	3417.5 (3008.5 to 3877.8)	3363.1 (2951 to 3826)	3468.6 (3052.8 to 3958.8)	-8.9 (-11.6 to -6)	-5.3 (-9.4 to -1)	-11.7 (-15.1 to -8.1)
	Prevalence	9050.9 (8001.1 to 10241.3)	8689.9 (7676.4 to 9856.8)	9365.3 (8174.6 to 10631.6)	8113.1 (7167.3 to 9239.2)	8115.3 (7135 to 9304.9)	8103.3 (7111.6 to 9177.6)	-10.4 (-14 to -7)	-6.6 (-11.5 to -1.2)	-13.5 (-18.4 to -8.7)
	YLDs	1017.8 (713.2 to 1365.1)	967.7 (681.7 to 1301.1)	1062.6 (751.7 to 1423.6)	915.1 (645.3 to 1226.4)	906.2 (633.8 to 1221.7)	922.9 (650.4 to 1241.3)	-10.1 (-14 to -6.5)	-6.4 (-11.8 to -0.2)	-13.1 (-18.5 to -7.9)

Province	Measure	Age-standardized rate (per 100,000)						% Change (1990 to 2019)		
		1990			2019					
		Both	Female	Male	Both	Female	Male	Both	Female	Male
**East Azarbayejan**	**Incidence**	3432.2 (2978.3 to 3944.3)	3136.7 (2742.1 to 3585.9)	3708.6 (3191.6 to 4305.2)	3007.7 (2622.3 to 3442.2)	2939.7 (2571.3 to 3332.1)	3073.7 (2628.8 to 3585.2)	-12.4 (-15.7 to -9.1)	-6.3 (-11 to -1.6)	-17.1 (-22.1 to -13)
	**Prevalence**	8045.1 (6834.8 to 9319.7)	7333.9 (6312.6 to 8445.2)	8708 (7238.8 to 10303.6)	6877.6 (5921.1 to 7910.2)	6786.5 (5952.2 to 7710.8)	6968.1 (5806.4 to 8183.9)	-14.5 (-19.5 to -9.7)	-7.5 (-13.8 to -0.8)	-20 (-26.3 to -14.1)
	**YLDs**	905.7 (637.2 to 1249.8)	817 (576.5 to 1106.2)	988.9 (695.2 to 1382.7)	773.1 (543.3 to 1045.1)	755.5 (532.3 to 1009.8)	790.4 (551.1 to 1089.9)	-14.6 (-19.8 to -9.3)	-7.5 (-14.2 to -0.5)	-20.1 (-26.6 to -13.8)
**Fars**	**Incidence**	3733.9 (3290.9 to 4227.6)	3537.9 (3118.5 to 4013.2)	3911.7 (3441.7 to 4438.1)	3405.4 (3006.1 to 3868.1)	3354.6 (2977.5 to 3802.9)	3455.9 (3027 to 3936.6)	-8.8 (-11.6 to -5.8)	-5.2 (-9.1 to -1)	-11.7 (-15.2 to -7.4)
	**Prevalence**	9003.2 (7956 to 10228.9)	8657.3 (7596.3 to 9890.4)	9308.6 (8174.6 to 10588.4)	8074.3 (7136.6 to 9160.4)	8087.9 (7131.2 to 9205)	8064.5 (7086.2 to 9189.5)	-10.3 (-13.7 to -6.8)	-6.6 (-11.7 to -1.4)	-13.4 (-18 to -8.5)
	**YLDs**	1007.5 (710.5 to 1346.1)	959.7 (683.2 to 1290.7)	1050.7 (736.7 to 1416.5)	905.8 (644.4 to 1211.6)	898.6 (634.6 to 1203.9)	913 (643.6 to 1227.7)	-10.1 (-13.8 to -6.3)	-6.4 (-11.8 to -0.9)	-13.1 (-18 to -7.6)
**Gilan**	**Incidence**	3774.4 (3321.7 to 4280.6)	3579.9 (3151.8 to 4038.3)	3958.5 (3481.8 to 4516.8)	3435.1 (3028.8 to 3920.2)	3385.7 (2998 to 3840.7)	3484.8 (3054.5 to 3969.8)	-9 (-11.9 to -6.3)	-5.4 (-9.1 to -1.4)	-12 (-16 to -8)
	**Prevalence**	9131.2 (8034.7 to 10340.2)	8778.2 (7684.5 to 9953.3)	9453 (8325.8 to 10753.5)	8190.2 (7213.1 to 9264.1)	8211.5 (7228.4 to 9307.4)	8170.9 (7168.8 to 9251.6)	-10.3 (-13.8 to -6.6)	-6.5 (-11.4 to -0.9)	-13.6 (-18.1 to -8.6)
	**YLDs**	1024.6 (719.4 to 1368.3)	975.3 (687 to 1310.6)	1070.9 (748.4 to 1432.4)	918.7 (647 to 1235.2)	911.3 (642.6 to 1219.2)	926.2 (646.2 to 1248.2)	-10.3 (-14.3 to -6.2)	-6.6 (-11.9 to -0.3)	-13.5 (-18.8 to -8.4)
**Golestan**	**Incidence**	3776.1 (3345.1 to 4288.7)	3579.2 (3165.2 to 4082.6)	3959.8 (3509 to 4487.1)	3450.5 (3045.1 to 3911.5)	3397 (2987.4 to 3846.4)	3505.8 (3076.5 to 3980.5)	-8.6 (-11.1 to -6)	-5.1 (-8.7 to -1.1)	-11.5 (-15 to -7.8)
	**Prevalence**	9146.7 (8014.6 to 10375.1)	8793.8 (7739.5 to 10036.4)	9464.9 (8237.3 to 10799.5)	8228.6 (7231.4 to 9325.9)	8241.8 (7251.9 to 9293.6)	8218.4 (7159.4 to 9345.7)	-10 (-13.4 to -6.4)	-6.3 (-11.4 to -1.3)	-13.2 (-18 to -7.9)
	**YLDs**	1028 (711.7 to 1382.6)	977.9 (682.9 to 1314.4)	1074.6 (743.2 to 1438.8)	924.2 (653.9 to 1233.3)	916.5 (643.4 to 1222.8)	932.4 (660.6 to 1247.7)	-10.1 (-13.9 to -6.1)	-6.3 (-11.8 to -0.7)	-13.2 (-18.5 to -7.6)

Province	Measure	Age-standardized rate (per 100,000)						% Change (1990 to 2019)		
		1990			2019					
		Both	Female	Male	Both	Female	Male	Both	Female	Male
Hamadan	Incidence	3770.6 (3338.4 to 4264.2)	3568.7 (3151.6 to 4031.6)	3960 (3503.1 to 4481.4)	3446 (3034.4 to 3906.7)	3394.3 (2995.3 to 3842)	3499 (3085.9 to 3964.4)	-8.6 (-11.6 to -5.6)	-4.9 (-8.9 to -0.7)	-11.6 (-15.5 to -7.5)
	Prevalence	9121.6 (7985.3 to 10292.4)	8768 (7653.8 to 9946.6)	9453.1 (8291.4 to 10661.1)	8199.2 (7238 to 9277.7)	8210.1 (7194.8 to 9361.8)	8195 (7177.2 to 9323.4)	-10.1 (-13.7 to -6.4)	-6.4 (-11.4 to -0.8)	-13.3 (-17.8 to -8.1)
	YLDs	1024.2 (723.7 to 1372.5)	973.5 (690.3 to 1297.5)	1072.2 (755.1 to 1445.9)	922.9 (643.3 to 1251.2)	914.8 (637.9 to 1232.7)	931.4 (648.9 to 1256.1)	-9.9 (-13.9 to -5.9)	-6 (-11.7 to -0.2)	-13.1 (-18.3 to -7.5)
Hormozgan	Incidence	3756.2 (3315.2 to 4264.1)	3549 (3124.1 to 4034.1)	3936.4 (3462 to 4466.4)	3421.2 (3025 to 3882.3)	3371.9 (2980.9 to 3821.3)	3472 (3057.7 to 3969.2)	-8.9 (-11.6 to -6.2)	-5 (-8.6 to -1.2)	-11.8 (-15.7 to -7.9)
	Prevalence	9054.6 (7966.4 to 10311.7)	8681 (7614.1 to 9905.8)	9369.1 (8186.1 to 10690.8)	8142.2 (7145.3 to 9218.3)	8152 (7130.1 to 9242.8)	8139.5 (7115.1 to 9239.9)	-10.1 (-13.4 to -6.3)	-6.1 (-11.8 to -0.5)	-13.1 (-17.8 to -7.9)
	YLDs	1016 (713.1 to 1368.2)	963.2 (679.9 to 1294.6)	1061.5 (736.1 to 1438.8)	915.6 (644.5 to 1223.3)	907.7 (640.5 to 1211.6)	923.6 (639.3 to 1236.8)	-9.9 (-13.7 to -5.6)	-5.8 (-11.6 to 0.3)	-13 (-18.1 to -7.1)
Ilam	Incidence	3778.5 (3330.2 to 4268.8)	3577.3 (3149.2 to 4045.8)	3954 (3467.2 to 4478.1)	3433.1 (3023.6 to 3879.7)	3384.1 (2985.8 to 3825.8)	3486.9 (3074.5 to 3966.5)	-9.1 (-11.7 to -6.6)	-5.4 (-9.3 to -1.2)	-11.8 (-15.2 to -7.9)
	Prevalence	9153.3 (8052.6 to 10309.7)	8789.2 (7748.3 to 9985.4)	9470.7 (8302.3 to 10727.7)	8171.1 (7190.7 to 9300.5)	8191 (7179.5 to 9293.1)	8167.6 (7144.5 to 9333.3)	-10.7 (-14.5 to -6.8)	-6.8 (-12 to -1.4)	-13.8 (-18.9 to -8.7)
	YLDs	1030.6 (721.7 to 1391.4)	977.7 (683.3 to 1319.2)	1077.1 (756 to 1447.9)	919 (646.9 to 1231.3)	910.9 (644.1 to 1224)	928.5 (648.4 to 1243.5)	-10.8 (-14.6 to -6.5)	-6.8 (-12.3 to -0.9)	-13.8 (-19 to -8.4)
Isfahan	Incidence	3708.9 (3277.3 to 4207.1)	3507.7 (3099.2 to 3952.9)	3886.1 (3425.3 to 4416.4)	3391.4 (2986 to 3845.5)	3343.8 (2961.6 to 3785.8)	3440.9 (3013.4 to 3927.4)	-8.6 (-11.2 to -5.8)	-4.7 (-8.7 to -0.7)	-11.5 (-15.4 to -7.4)
	Prevalence	8914.6 (7801.2 to 10132.8)	8554.1 (7457.7 to 9705.2)	9224.2 (8060.7 to 10524)	8028.2 (7038.4 to 9090.2)	8050 (7075.4 to 9123.7)	8015.4 (6999.7 to 9091.9)	-9.9 (-13.4 to -6.2)	-5.9 (-10.8 to -0.7)	-13.1 (-18 to -8.2)
	YLDs	1003.1 (704.2 to 1350.9)	951.9 (670.6 to 1280.7)	1048.2 (734.2 to 1414.5)	902.3 (636.5 to 1215.1)	895 (628 to 1197.9)	910.2 (639.5 to 1231.5)	-10.1 (-13.7 to -6.1)	-6 (-11.6 to -0.2)	-13.2 (-18.5 to -7.8)

Province	Measure	Age-standardized rate (per 100,000)						% Change (1990 to 2019)		
		1990			2019					
		Both	Female	Male	Both	Female	Male	Both	Female	Male
Kerman	Incidence	3773 (3339.3 to 4289.5)	3575.2 (3143 to 4057.3)	3954.6 (3482.9 to 4489.5)	3438.6 (3040.2 to 3874.1)	3387.2 (2999.6 to 3808.2)	3486.2 (3072.7 to 3984.6)	-8.9 (-12 to -5.7)	-5.3 (-9.4 to -0.6)	-11.8 (-16 to -7.7)
	Prevalence	9127.4 (7982.8 to 10352.9)	8771.6 (7668 to 9932.9)	9445.2 (8225.6 to 10714.6)	8195.2 (7208.5 to 9265.2)	8207.4 (7202.5 to 9294.9)	8180.3 (7167.4 to 9302.3)	-10.2 (-13.9 to -6.5)	-6.4 (-11.4 to -1.3)	-13.4 (-18.1 to -8.2)
	YLDs	1021 (720.3 to 1365.1)	970.8 (678.7 to 1302.9)	1067 (744.1 to 1435.7)	915.5 (646.4 to 1214.6)	907.5 (643.4 to 1214.8)	922.6 (644.8 to 1235.9)	-10.3 (-14.2 to -6.3)	-6.5 (-12.2 to -0.7)	-13.5 (-19 to -7.9)
Kermanshah	Incidence	3772.5 (3338 to 4262.7)	3571.8 (3155.5 to 4033.1)	3956.1 (3480.6 to 4505.2)	3424.9 (3024.4 to 3884.6)	3382.9 (2978.2 to 3831.3)	3472.2 (3059.6 to 3956.1)	-9.2 (-12.3 to -6.6)	-5.3 (-9.8 to -0.9)	-12.2 (-15.7 to -8.5)
	Prevalence	9125.2 (7997.3 to 10326.6)	8773 (7662.9 to 9910.4)	9447.4 (8288.9 to 10783)	8167.9 (7186.6 to 9258.5)	8192.6 (7170.2 to 9304.9)	8158.7 (7148.9 to 9266.9)	-10.5 (-13.8 to -7)	-6.6 (-11.5 to -1.2)	-13.6 (-18.2 to -8.7)
	YLDs	1025 (715.8 to 1372.1)	974.7 (680.6 to 1316.3)	1071.5 (745.7 to 1444)	917.3 (647.7 to 1246.2)	911.2 (637.9 to 1228.8)	925 (644.1 to 1248.8)	-10.5 (-14.2 to -6.9)	-6.5 (-11.7 to -0.7)	-13.7 (-18.7 to -8.4)
Khorasan-e-Razavi	Incidence	3754.5 (3312 to 4262.7)	3555.7 (3140.6 to 4048)	3942 (3457.8 to 4463)	3422.5 (3017.2 to 3870.8)	3375.6 (2985.3 to 3831.9)	3470 (3048 to 3917.9)	-8.8 (-11.6 to -5.8)	-5.1 (-9.3 to -0.9)	-12 (-15.2 to -8)
	Prevalence	9079.1 (7981.9 to 10312.3)	8731.8 (7667.9 to 9977.4)	9400.7 (8236.5 to 10626.6)	8128.9 (7179.7 to 9169.2)	8139.7 (7239.7 to 9196)	8118.8 (7118.3 to 9183.5)	-10.5 (-14 to -6.8)	-6.8 (-12 to -1.2)	-13.6 (-18.4 to -9)
	YLDs	1017.1 (712.8 to 1367.4)	967.6 (685.6 to 1297.2)	1063.7 (736.7 to 1429.5)	909.2 (638.3 to 1219.8)	901.5 (643.6 to 1207.3)	916.9 (640.3 to 1227.2)	-10.6 (-14.2 to -6.7)	-6.8 (-12.3 to -1.2)	-13.8 (-18.8 to -8.6)
Khuzestan	Incidence	3735.4 (3294 to 4207.2)	3537.7 (3125.6 to 3989)	3914.9 (3447.6 to 4426.1)	3407 (3003.7 to 3881.3)	3359.5 (2959.7 to 3813.9)	3455.8 (3032.4 to 3943.8)	-8.8 (-11.4 to -6)	-5 (-9.2 to -1.1)	-11.7 (-15.3 to -8.1)
	Prevalence	9001.3 (7949.3 to 10245.9)	8636.6 (7591.7 to 9783)	9325.6 (8180.8 to 10636.7)	8077.7 (7121.6 to 9150.7)	8090.1 (7108 to 9195.5)	8071.2 (7064.7 to 9152.4)	-10.3 (-13.7 to -6.5)	-6.3 (-11.6 to -0.7)	-13.5 (-17.8 to -8.1)
	YLDs	1006.6 (706.2 to 1348.3)	955.7 (677 to 1280.7)	1052.9 (733.1 to 1416.2)	904.6 (640.1 to 1196.5)	896.8 (633.4 to 1198.3)	912.8 (641.5 to 1219.4)	-10.1 (-13.6 to -6.1)	-6.2 (-11.7 to -0.2)	-13.3 (-18.1 to -8)

Province	Measure	Age-standardized rate (per 100,000)						% Change (1990 to 2019)		
		1990			2019					
		Both	Female	Male	Both	Female	Male	Both	Female	Male
Kohgiluyeh and Boyer-Ahmad	Incidence	3757.4 (3324.5 to 4250.8)	3562.1 (3132.2 to 4037.9)	3928.5 (3474 to 4462.6)	3406.2 (3003.4 to 3877.8)	3362.9 (2945.6 to 3834.5)	3454.5 (3040.8 to 3946)	-9.3 (-11.9 to -6.8)	-5.6 (-9.2 to -1.4)	-12.1 (-15.7 to -8.6)
	Prevalence	9067.1 (7946.4 to 10232.3)	8703.9 (7605 to 9929.6)	9377.2 (8213.3 to 10693.5)	8090.5 (7098 to 9179.7)	8110.7 (7141.9 to 9230)	8089.3 (7097.9 to 9197.7)	-10.8 (-14.1 to -7.1)	-6.8 (-11.7 to -1.5)	-13.7 (-18.3 to -8.5)
	YLDs	1016 (708.2 to 1360.7)	964.9 (675.6 to 1293.5)	1060.5 (734 to 1424.6)	911 (638.2 to 1221.3)	903.7 (636.6 to 1214.7)	919.6 (645.8 to 1235.3)	-10.3 (-13.9 to -6.6)	-6.3 (-11.7 to -0.6)	-13.3 (-17.8 to -7.8)
Kurdistan	Incidence	3787.3 (3349.5 to 4304.5)	3587.4 (3163.6 to 4081.6)	3973.1 (3504.6 to 4532.4)	3447.1 (3032.6 to 3891.9)	3392.7 (2997.5 to 3833.3)	3501.6 (3066.3 to 3978.3)	-9 (-11.7 to -6.1)	-5.4 (-9.5 to -1.1)	-11.9 (-15.5 to -8.1)
	Prevalence	9165.5 (8042.7 to 10413.9)	8807.2 (7732.3 to 9936)	9496.1 (8259.4 to 10879.3)	8224.7 (7197.4 to 9271.6)	8236 (7223.5 to 9339.1)	8217.1 (7175.9 to 9281.2)	-10.3 (-13.8 to -6.8)	-6.5 (-11.8 to -1.6)	-13.5 (-18 to -8.2)
	YLDs	1029.4 (718.2 to 1392.1)	979.3 (688.1 to 1329.9)	1076.1 (745 to 1459.6)	926 (647.7 to 1244.3)	918.2 (636.7 to 1241.5)	933.8 (652.1 to 1260.2)	-10 (-13.7 to -6.1)	-6.2 (-11.9 to -0.9)	-13.2 (-18.3 to -7.4)
Lorestan	Incidence	3781 (3341.6 to 4275.1)	3583.7 (3173.3 to 4065.3)	3960.4 (3483.6 to 4489.3)	3442.1 (3035.6 to 3902.2)	3393.9 (2995.5 to 3845.5)	3490.8 (3079.1 to 3964.1)	-9 (-11.6 to -6.4)	-5.3 (-9.4 to -1.6)	-11.9 (-15.5 to -8.2)
	Prevalence	9152.8 (8039.1 to 10364.3)	8797.4 (7721.9 to 9970.4)	9473.9 (8322 to 10730.5)	8214.9 (7222.2 to 9298.3)	8222 (7203.4 to 9312.5)	8209.3 (7208 to 9397)	-10.2 (-13.5 to -6.8)	-6.5 (-11.6 to -1.2)	-13.3 (-17.8 to -8.2)
	YLDs	1027.5 (720.8 to 1378.3)	977.3 (684.3 to 1310)	1073.5 (756 to 1452.4)	925.3 (651.8 to 1237)	917 (646.8 to 1234)	933.6 (652.7 to 1259.8)	-9.9 (-13.5 to -6.1)	-6.2 (-11.9 to -0.5)	-13 (-17.8 to -7.5)
Markazi	Incidence	3744.9 (3319.4 to 4230.9)	3550.8 (3147.6 to 4010.5)	3937.1 (3473.1 to 4471.5)	3415.9 (3018.7 to 3877.8)	3364 (2974.1 to 3830.3)	3467.6 (3058.7 to 3940.5)	-8.8 (-11.4 to -6.1)	-5.3 (-9.2 to -1.6)	-11.9 (-15.4 to -8.2)
	Prevalence	9042.6 (7949.6 to 10276.4)	8692.3 (7564 to 9940)	9388.2 (8283.9 to 10671.6)	8116.2 (7173.4 to 9173.9)	8129.1 (7125.8 to 9170.6)	8105.9 (7083.3 to 9232.5)	-10.2 (-13.5 to -6.7)	-6.5 (-11.3 to -1)	-13.7 (-18.1 to -8.9)
	YLDs	1012.1 (717.8 to 1358.3)	962.8 (681.2 to 1293.5)	1061.3 (742.7 to 1419)	911.8 (641.4 to 1224)	904.1 (637.4 to 1215.3)	919.5 (642.1 to 1241)	-9.9 (-13.8 to -6)	-6.1 (-11.5 to -0.1)	-13.4 (-18.4 to -8)

Province	Measure	Age-standardized rate (per 100,000)						% Change (1990 to 2019)		
		1990			2019					
		Both	Female	Male	Both	Female	Male	Both	Female	Male
**Mazandaran**	**Incidence**	4673.2 (4154.2 to 5247.1)	5404.8 (4767.3 to 6060.7)	3935 (3477.4 to 4500.1)	4349.6 (3861.7 to 4869.3)	5238.5 (4661.1 to 5871.7)	3469.4 (3058.4 to 3949.1)	-6.9 (-9.6 to -4)	-3.1 (-6.7 to 1.2)	-11.8 (-15.4 to -8)
	**Prevalence**	14417 (12880.7 to 16189)	19391.3 (17314 to 21800.3)	9396.4 (8253.8 to 10664.6)	13259.6 (11812 to 14841.5)	18416.3 (16436.1 to 20627.7)	8120.9 (7085.3 to 9243.5)	-8 (-11.4 to -4.3)	-5 (-9.5 to -0.1)	-13.6 (-17.9 to -9)
	**YLDs**	1602.6 (1131.3 to 2146.6)	2137.7 (1515.3 to 2866.4)	1064.5 (742.1 to 1435)	1476.8 (1042.6 to 1972.2)	2034.3 (1435.4 to 2714.2)	921.6 (644.6 to 1232.1)	-7.9 (-11.6 to -4.1)	-4.8 (-9.7 to 0.4)	-13.4 (-18.2 to -8.3)
**North Khorasan**	**Incidence**	3792.8 (3351.6 to 4301.8)	3593.4 (3154.1 to 4077.9)	3982.9 (3518.7 to 4538.9)	3453.5 (3067.3 to 3912.1)	3399.3 (2995.7 to 3854.4)	3513 (3107.7 to 3977.9)	-8.9 (-11.7 to -6.1)	-5.4 (-9.5 to -1.4)	-11.8 (-15.4 to -8.2)
	**Prevalence**	9201.6 (8074.7 to 10461.7)	8845.7 (7721.6 to 10050.4)	9539 (8321.6 to 10855.3)	8244 (7230.7 to 9287.7)	8263.6 (7221.4 to 9370.5)	8238.2 (7198.2 to 9309.5)	-10.4 (-13.8 to -6.7)	-6.6 (-11.8 to -1.4)	-13.6 (-18.2 to -8.7)
	**YLDs**	1030.2 (722.6 to 1376.2)	979.5 (685.7 to 1317.2)	1079 (756.9 to 1455.3)	924 (650.6 to 1235.5)	916.2 (643.8 to 1226.9)	933.2 (657.1 to 1258.7)	-10.3 (-14.1 to -6.2)	-6.5 (-12.1 to -0.6)	-13.5 (-18.5 to -8.4)
**Qazvin**	**Incidence**	3760.1 (3323.9 to 4264.3)	3564.4 (3160.8 to 4028.3)	3939.8 (3469.3 to 4481.5)	3419 (3019.1 to 3887.7)	3367 (2968 to 3837.4)	3466.8 (3050.5 to 3947)	-9.1 (-12 to -6.5)	-5.5 (-9.5 to -1.7)	-12 (-15.5 to -8.2)
	**Prevalence**	9081.4 (8011.3 to 10321.9)	8727.4 (7652.9 to 9946.8)	9397.2 (8260.1 to 10648.6)	8121.2 (7100.2 to 9183.2)	8133.4 (7062.2 to 9178.1)	8099.7 (7062.8 to 9182.5)	-10.6 (-13.7 to -7.2)	-6.8 (-11.6 to -1.2)	-13.8 (-18.2 to -9)
	**YLDs**	1020.1 (720.2 to 1365.4)	969.6 (679.3 to 1299.9)	1066.5 (752.1 to 1423.7)	913.7 (641.7 to 1224.1)	905.9 (639.8 to 1224.3)	920.3 (641.3 to 1242.5)	-10.4 (-14.1 to -6.5)	-6.6 (-11.8 to -0.6)	-13.7 (-18.6 to -8.6)
**Qom**	**Incidence**	3683.5 (3255 to 4182.5)	3483.8 (3077.6 to 3961.4)	3862.4 (3404.4 to 4396.1)	3366.8 (2975.7 to 3789)	3322.4 (2929.9 to 3745.1)	3414.5 (3006.8 to 3863.8)	-8.6 (-11.3 to -5.9)	-4.6 (-8.5 to -0.5)	-11.6 (-15.2 to -7.9)
	**Prevalence**	8818 (7748.8 to 9964.3)	8463.1 (7399.3 to 9577.4)	9129 (8013.6 to 10343)	7941.9 (7002.7 to 8924.1)	7960.4 (7005.3 to 8959)	7938.3 (6949.2 to 9013.9)	-9.9 (-13.6 to -5.9)	-5.9 (-10.9 to -0.4)	-13 (-17.8 to -7.7)
	**YLDs**	984.8 (700 to 1321.7)	935.3 (661.6 to 1260.7)	1028.8 (729.2 to 1376.1)	892 (627.3 to 1188.4)	884.5 (627 to 1186)	900.4 (630 to 1210.9)	-9.4 (-13.2 to -5.1)	-5.4 (-11.1 to 0.7)	-12.5 (-17.6 to -6.8)

Province	Measure	Age-standardized rate (per 100,000)						% Change (1990 to 2019)		
		1990			2019					
		Both	Female	Male	Both	Female	Male	Both	Female	Male
Semnan	Incidence	3723 (3295 to 4228.5)	3527.8 (3126.8 to 3992)	3902.1 (3433 to 4456.2)	3399 (2985 to 3840.7)	3348.9 (2942.5 to 3793.4)	3444.9 (3028.6 to 3907.1)	-8.7 (-11.5 to -5.7)	-5.1 (-9 to -0.8)	-11.7 (-15.2 to -7.5)
	Prevalence	8953.1 (7901.4 to 10202.7)	8606 (7541.6 to 9821.7)	9260.9 (8172.7 to 10566.8)	8047.8 (7096.7 to 9078.1)	8053.2 (7084.5 to 9115.3)	8033.5 (7063.3 to 9116.7)	-10.1 (-13.7 to -6.5)	-6.4 (-11.6 to -1.2)	-13.3 (-18 to -8.5)
	YLDs	1002 (703.4 to 1337.9)	954.4 (671.5 to 1286.6)	1045.7 (732.6 to 1398.3)	904.3 (633 to 1209.3)	895.9 (632.4 to 1195.7)	911.8 (634.2 to 1225.8)	-9.7 (-13.6 to -5.8)	-6.1 (-11.7 to -0.6)	-12.8 (-18.2 to -7.6)
Sistan and Baluchistan	Incidence	3729.7 (3299.9 to 4213.4)	3523.7 (3110.2 to 3976)	3908.4 (3455.5 to 4429.1)	3401.7 (3009.8 to 3849.2)	3354.3 (2959.7 to 3788.9)	3450.3 (3047.4 to 3930.8)	-8.8 (-11.5 to -5.9)	-4.8 (-8.6 to -0.7)	-11.7 (-15.4 to -8)
	Prevalence	8969 (7877.9 to 10167.9)	8592.4 (7542.7 to 9765.2)	9286.4 (8097.3 to 10581.2)	8070.1 (7088 to 9108.8)	8087.7 (7088.8 to 9161.1)	8056.7 (7077.1 to 9168.9)	-10 (-13.4 to -6.7)	-5.9 (-10.8 to -1)	-13.2 (-18 to -8.5)
	YLDs	1005.9 (711.6 to 1354.1)	953.1 (669.8 to 1272)	1051.3 (746.5 to 1417.6)	906.7 (634.5 to 1221.2)	899.6 (632.9 to 1208.7)	914.2 (635.8 to 1225.7)	-9.9 (-13.7 to -6.3)	-5.6 (-11.1 to 0)	-13 (-18.2 to -7.8)
South Khorasan	Incidence	3774 (3323.7 to 4273)	3577 (3164.5 to 4039.8)	3964 (3463.8 to 4516.9)	3443.8 (3049.9 to 3910.1)	3393.9 (2983.2 to 3836.9)	3492.8 (3094.2 to 3977)	-8.7 (-11.6 to -5.9)	-5.1 (-9.4 to -0.9)	-11.9 (-15.5 to -8.2)
	Prevalence	9137.3 (7989.6 to 10352.7)	8789.2 (7638.2 to 9965.1)	9470.2 (8304.5 to 10732.1)	8216.3 (7246.1 to 9258.9)	8225.8 (7218.7 to 9314.3)	8203.7 (7148.1 to 9268.7)	-10.1 (-13.6 to -6.4)	-6.4 (-11.5 to -1)	-13.4 (-18.1 to -8.4)
	YLDs	1023.5 (722 to 1363.6)	973.3 (684.4 to 1298.1)	1072 (752.9 to 1440.4)	919.7 (648.8 to 1228)	910.7 (642.6 to 1219)	928.5 (653.7 to 1245.6)	-10.1 (-13.9 to -6.1)	-6.4 (-11.9 to -0.3)	-13.4 (-18.7 to -8)
Tehran	Incidence	3972.1 (3538.7 to 4461.8)	3480 (3074.6 to 3935.9)	4429.2 (3921.3 to 4999.8)	3718.6 (3298.9 to 4177.5)	3321.4 (2930.6 to 3769.6)	4117.6 (3669.3 to 4636)	-6.4 (-9.3 to -3.1)	-4.6 (-8.3 to -0.4)	-7 (-11.1 to -2.4)
	Prevalence	9850.3 (8757.4 to 11046.5)	8439.6 (7375.4 to 9568.4)	11174.2 (10035.6 to 12501.9)	9174.2 (8209.6 to 10278.3)	7956.9 (7050.1 to 8992.4)	10393.5 (9288.8 to 11624.7)	-6.9 (-10.3 to -3.2)	-5.7 (-10.7 to -0.7)	-7 (-11.8 to -1.9)
	YLDs	1105.8 (775.6 to 1492.8)	934.3 (660 to 1250.1)	1266.5 (887.9 to 1716.5)	1029.4 (728 to 1384.9)	881.2 (625.9 to 1181.9)	1177.7 (834.2 to 1583.8)	-6.9 (-10.6 to -2.9)	-5.7 (-10.7 to 0.2)	-7 (-12.2 to -1.5)

Province	Measure	Age-standardized rate (per 100,000)						% Change (1990 to 2019)		
		1990			2019					
		Both	Female	Male	Both	Female	Male	Both	Female	Male
West Azarbayejan	Incidence	3775.5 (3327.7 to 4276.4)	3571.4 (3140.5 to 4056.2)	3963.5 (3483 to 4515.4)	3448.9 (3045.7 to 3906.5)	3396.3 (2997.5 to 3843.8)	3499.4 (3078.7 to 3980.3)	-8.7 (-11.3 to -5.8)	-4.9 (-8.8 to -0.9)	-11.7 (-15.3 to -7.4)
	Prevalence	9144.2 (8051.7 to 10310)	8790.1 (7686.5 to 9974.3)	9465 (8286.6 to 10754.9)	8237.4 (7215.2 to 9323.1)	8250.3 (7206 to 9403.1)	8221.1 (7156.9 to 9332.2)	-9.9 (-13.3 to -6.5)	-6.1 (-10.9 to -0.8)	-13.1 (-18 to -8.1)
	YLDs	1026.1 (720 to 1364.1)	974.2 (688.1 to 1289.3)	1074.1 (748.2 to 1438.8)	925.1 (651.5 to 1239.6)	915.4 (648 to 1226)	934.4 (647.6 to 1254.8)	-9.8 (-13.2 to -6.1)	-6 (-11.3 to -0.2)	-13 (-18.1 to -7.7)
Yazd	Incidence	3728.9 (3287.9 to 4224.6)	3533.9 (3108.3 to 3997.3)	3894.6 (3419.7 to 4432.7)	3402.9 (3001.2 to 3847.8)	3349.3 (2965.7 to 3797.2)	3449 (3025.9 to 3912.8)	-8.7 (-11.5 to -6)	-5.2 (-9.1 to -1.2)	-11.4 (-15.1 to -7.8)
	Prevalence	8983 (7910.2 to 10209.2)	8614.7 (7596.4 to 9828.7)	9281.7 (8110.8 to 10564.5)	8055 (7073 to 9082.3)	8052.4 (7092.3 to 9086.3)	8045.1 (7017.1 to 9106.1)	-10.3 (-13.8 to -6.4)	-6.5 (-11.3 to -1.4)	-13.3 (-18.4 to -8.2)
	YLDs	1006.6 (706.5 to 1353)	956.1 (677 to 1284.7)	1049.1 (727.4 to 1406.4)	904.3 (634.8 to 1206.6)	895.4 (633.7 to 1195.6)	911.4 (638.4 to 1221.6)	-10.2 (-14.3 to -6.1)	-6.4 (-12.1 to -1)	-13.1 (-18.6 to -7.4)
Zanjan	Incidence	3777.4 (3347.3 to 4290.7)	3579.2 (3165.2 to 4082.6)	3959.8 (3509 to 4487.1)	3456.2 (3034.3 to 3928.8)	3403 (2989.1 to 3868.5)	3507.1 (3082.8 to 3978.2)	-8.5 (-11.3 to -5.9)	-4.9 (-8.9 to -1.1)	-11.4 (-15.3 to -8)
	Prevalence	9149.5 (8019.4 to 10375.8)	8793.8 (7739.5 to 10036.4)	9464.9 (8237.3 to 10799.5)	8241.6 (7244.5 to 9338.3)	8247.5 (7235.5 to 9360.1)	8228.6 (7186.8 to 9304.6)	-9.9 (-13.2 to -6.4)	-6.2 (-11 to -1.2)	-13.1 (-17.4 to -8.4)
	YLDs	1024.2 (716.4 to 1364.9)	974.6 (687.8 to 1312.5)	1070 (740.8 to 1439.3)	925.4 (649.6 to 1248.7)	916.7 (643 to 1235.3)	933.2 (655.1 to 1265.5)	-9.6 (-13.4 to -5.7)	-5.9 (-11.6 to -0.5)	-12.8 (-17.6 to -7.4)

Data in parenthesis are 95% uncertainty intervals (UI), YLDs= Years Lived with Disability
